# Phylogenetic Patterns in the Microbial Response to Resource Availability: Amino Acid Incorporation in San Francisco Bay

**DOI:** 10.1371/journal.pone.0095842

**Published:** 2014-04-21

**Authors:** Xavier Mayali, Peter K. Weber, Shalini Mabery, Jennifer Pett-Ridge

**Affiliations:** Physical and Life Science Directorate, Lawrence Livermore National Laboratory, Livermore California, United States of America; Uppsala University, Sweden

## Abstract

Aquatic microorganisms are typically identified as either oligotrophic or copiotrophic, representing trophic strategies adapted to low or high nutrient concentrations, respectively. Here, we sought to take steps towards identifying these and additional adaptations to nutrient availability with a quantitative analysis of microbial resource use in mixed communities. We incubated an estuarine microbial community with stable isotope labeled amino acids (AAs) at concentrations spanning three orders of magnitude, followed by taxon-specific quantitation of isotopic incorporation using NanoSIMS analysis of high-density microarrays. The resulting data revealed that trophic response to AA availability falls along a continuum between copiotrophy and oligotrophy, and high and low activity. To illustrate strategies along this continuum more simply, we statistically categorized microbial taxa among three trophic types, based on their incorporation responses to increasing resource concentration. The data indicated that taxa with copiotrophic-like resource use were not necessarily the most active, and taxa with oligotrophic-like resource use were not always the least active. Two of the trophic strategies were not randomly distributed throughout a 16S rDNA phylogeny, suggesting they are under selective pressure in this ecosystem and that a link exists between evolutionary relatedness and substrate affinity. The diversity of strategies to adapt to differences in resource availability highlights the need to expand our understanding of microbial interactions with organic matter in order to better predict microbial responses to a changing environment.

## Introduction

Microbes dominate the biomass and biogeochemical activity of aquatic environments on a global scale [1]. Niche differentiation for resource acquisition, among other factors, allows the co-existence of a high diversity of aquatic microbes in the same volume of water, as different organisms are adapted to utilize the same resources but at different concentrations [2]. For example, ammonium and phosphate uptake at micromolar concentrations is dominated by larger organisms (mostly eukaryotes), whereas at nanomolar concentrations, organisms smaller than 1 micron (bacteria and archaea) dominate uptake [3]. A similar phenomenon has been described in marine ammonia oxidizing communities, where ammonia-oxidizing bacteria dominate activity at higher ammonium concentrations and ammonia-oxidizing archaea dominate activity at lower concentrations [4]. Niche differentiation likely also occurs at lower taxonomic levels; for example some microbial phyla appear to respond differently to varying concentrations of leucine, in particular during different stages of an algal bloom [5].

Niche differentiation may occur because most aquatic microbes acquire nutrients from their environment one molecule at a time through substrate-specific transporters often coupled with cell-bound extracellular enzymes that interact directly with dissolved substrates on a molecular level [6]. Different transporters have substrate-binding efficiencies adapted to different concentrations [7], allowing microorganisms to respond to the resource patchiness of seawater [8]. In the context of both inorganic and organic nutrient acquisition, microbes have been classified into two main guilds with respect to their adaptation to substrate concentrations: oligotrophs and copiotrophs [9], the latter also referred to as opportunitrophs [2]. Oligotrophs are generally small cells with small genomes, are characterized by slow, steady growth, and numerically dominate in low nutrient environments such as sub-tropical oceanic gyres [10]. Copiotrophs, with larger cells and more extensive genomes [11], subsist on a feast-or-famine approach (fast growth interspersed with inactivity) and numerically dominate in high nutrient coasts and estuaries. While the use of these two terms has been quite beneficial for our conceptual understanding of microbial biogeochemical cycling, a more nuanced theoretical framework is needed to reflect the complexity of resource heterogeneity in nature [6]. For example, we might consider that trophic strategy is distributed on a continuum between oligotrophy and copiotrophy, with several intermediate states. An improved understanding of trophic strategies is important to reliably predict microbial responses to changes in resource availability likely to be caused by anthropogenic forces such as eutrophication and pollution.

Here, we expand on the classification scheme of oligotroph vs. copiotroph to more fully categorize the range of microbial substrate utilization strategies that enable microbes to co-exist through trophic niche differentiation. To develop these categories, we tested how microbial taxa responded in their incorporation of amino acids (AAs), organic substrates commonly utilized by aquatic microorganisms [12], [13]. A number of previous studies (using bulk methods) have shown that AAs provide a large fraction of C and N requirements to aquatic bacterial communities [14], [15]. Subsequent studies, with more modern techniques, have shown evidence that dominant marine taxa (such as SAR11) have the genetic capability to uptake AAs [16], [17] and express them [13], [18], and incubation experiments have validated these hypotheses with cell-specific methods [19], [20]. To quantify AA incorporation at three different concentrations, we used Chip-SIP, a high-throughput method of quantifying taxon-specific incorporation of stable isotope labeled substrates [21]. Our results revealed a relatively high degree of complexity in the microbial community response to varying AA concentrations, and suggest that substrate affinity may be an evolutionarily conserved trait.

## Materials and Methods

### Incubation of Field Samples

Surface water was collected at the public pier in Berkeley, CA USA (37°51′46.67″N, 122°19′3.23″W) on 03/17/2011 and brought back to the laboratory within one hour in a cooler. No specific permissions were required for collection of seawater at this location and our studies did not involve endangered or protected species. Glass bottles (500 ml) were filled without air space and dark incubated at 14°C. Samples were incubated in triplicate bottles with 5 µM (High), 500 nM (Medium), and 50 nM (Low) mixed amino acids (99 atm % ^15^N labeled; Omicron Biochemicals Inc., South Bend, IN, USA), collected by filtration after 12 hrs and frozen at −80°C. RNA extracts from triplicate incubations were combined and hybridized to a system-specific high-density 16S microarray (see description below) and subsequently analyzed by isotopic imaging with a nano secondary ion mass spectrometer (NanoSIMS 50, Cameca, France). RNA extracts from all three treatments (H = High, M = Medium, and L = Low) were combined for fluorescent labeling (see below) in order to compare the three concentration treatments to one another.

### RNA Extraction and Labeling

The RNA samples were separately analyzed for fluorescence (with a microarray scanner) and isotopic enrichment (with the NanoSIMS), because fluorescence labeling causes dilution of the isotopic signal. RNA from frozen filters was extracted with the RNEasy kit according to manufacturer’s instructions (Qiagen, Hilden, Germany). Alexafluor 532 labeling was done with the Ulysis kit (Life Technologies, Carlsbad, CA, USA) on the samples for fluorescent labeling for 10 min at 90°C (2 µL RNA, 10 µL labeling buffer, 2 µL Alexafluor reagent), followed by fragmentation. All RNA (fluorescently labeled or not) was fragmented using 1X fragmentation buffer (Affymetrix, Santa Clara, CA, USA) for 10 min at 90°C and concentrated by isopropanol precipitation to a final concentration of 500 ng µL^−1^.

### Microarray Hybridization and NanoSIMS

A phylogenetic microarray designed to target San Francisco Bay microbial communities (Figure S3, Table S1) included probes specific to ribosomal RNA operational taxonomic units (OTUs) as well as more general probes targeting the three domains of life (Bacteria, Archaea, Eukarya), two abundant marine bacterial orders (*Alteromonadales* and *Rhodobacterales*) and the genus *Polaribacter* [21]. Due to the variability in 16S diversity in different parts of the 16S phylogeny, there was no standard % similarity or taxonomic classification (genus, species, strain, etc.) that we could use to describe the lowest phylogenetic level targeted by the array. In general, taxa were targeted at the lowest possible phylogenetic level, subordinate to the genus level. To synthesize the microarrays, glass slides coated with indium-tin oxide (ITO; Sigma-Aldrich, St. Louis, MO, USA) were coated with silane Super Epoxy 2 (Arrayit Corporation, Sunnyvale, CA, USA) to provide a starting matrix for DNA synthesis. Custom-designed microarrays (spot size = 17 µm) were synthesized using a photolabile deprotection strategy [22] on the LLNL Maskless Array Synthesizer (Roche Nimblegen, Madison, WI, USA). Reagents for synthesis were delivered through an Expedite system (PerSeptive Biosystems, Framingham, MA, USA). For array hybridization, RNA samples (1 µg) in 1X Hybridization buffer (Roche Nimblegen, Madison, WI, USA) were placed in Nimblegen X4 mixer slides and incubated inside a Maui hybridization system (BioMicro Systems, Salt Lake City, UT, USA) for 18 hrs at 42°C and subsequently washed according to the manufacturer’s instructions (Roche Nimblegen, Madison, WI, USA). Arrays with fluorescently labeled RNA were imaged with a Genepix 4000B fluorescence scanner (Molecular Devices, Sunnyvale, CA, USA) at pmt = 650 units. Secondary ion mass spectrometry (SIMS) analysis of microarrays hybridized with ^15^N rRNA was performed at LLNL with a Cameca NanoSIMS 50 (Cameca, Gennevilliers, France). A Cs+ primary ion beam was used to enhance the generation of negative secondary ions. Nitrogen isotopic ratios were determined by electrostatic peak switching on electron multipliers in pulse counting mode, measuring ^12^C^14^N^−^ and ^12^C^15^N^−^ simultaneously. More details of the instrument parameters are provided elsewhere [21]. Ion images were stitched together and processed to generate isotopic ratios with custom software (LIMAGE, L. Nittler, Carnegie Institution of Washington). Isotopic ratios were converted to delta (permil) values using δ = [(R_meas_/R_standard_) –1]×1000, where R_meas_ is the measured ratio and R_standard_ is the ratio measured in unhybridized locations of the sample. All fluorescence and NanoSIMS data have been deposited to NCBI’s Gene Expression Omnibus archive under record number GSE56119.

### Data Analyses

For each phylotype, isotopic enrichment of individual probe spots was plotted versus probe fluorescence and a linear regression slope was calculated. This slope (permil/fluorescence), which we refer to as the hybridization-corrected enrichment (HCE), is a metric that can be used to compare the relative incorporation of a given substrate by different taxa, or the relative incorporation by one taxon across different treatments [21]. Two procedures were carried out to assign phylotypes to guilds according to AA incorporation patterns. First, taxa were assigned to groups based on activity level, defined by HCE values for a single concentration. We note that this assignment does not take into consideration the response to different substrate concentrations. The HCE values at a single concentration followed a lognormal distribution with a small number of high values and mostly low to intermediate values (Fig. S1a). While the distribution of HCEs represents a continuum of activity, for simplicity we aimed to separate taxa into two groups: the first with high activity and the second with low activity. We set the cutoff response to distinguish highly active and less active taxa at a value of 50% of the maximum, which corresponded to a local high in the percent change along the distribution (Fig. S1b). The second procedure to assign phylotypes to trophic guilds involved the examination of individual taxa and their incorporation response (i.e., HCE values) under the three different concentrations (H, M, L) tested. For each taxon, we used an analysis of covariance (ANCOVA with a standard least squares model) to determine if concentration (or concentration and fluorescence together in a nested test) had a significant effect on isotopic enrichment (p<0.05), with the null hypothesis being no concentration effect (H≈M≈L). This procedure tested whether the slope of enrichment/fluorescence (HCE) or the y-intercept (isotopic enrichment) were significantly different for the different substrate concentrations. For each taxon, if the ANCOVA was not significant, it meant that the Null hypothesis (H≈M≈L) was not rejected (treatment H/M/L had no effect on the relationship between fluorescence and enrichment). If the ANCOVA was significant, then it means that at least 1 of the 3 treatments had a significant effect on isotopic enrichment. In theory, all the possibilities were: H>M, H>L, M>L, L>H, L>M, and/or M>H. However, since lower substrate concentrations should not lead to higher isotope incorporation, the actual possibilities were H>M∼L, H∼M>L, or H>M>L. With a post-hoc test, we examined each ANCOVA result (for each taxon) individually to find out which treatments were significantly different. We did not find any taxa that showed the pattern H>M∼L, thus the only trophic strategies identified were the Null hypothesis (H≈M≈L), H>M>L and H≈M>L.

HCE values for each treatment were normalized to the sum of the HCE values for the three concentration treatments and were plotted on a barycentric ternary diagram (taxa not isotopically enriched were not plotted). Trophic strategies were mapped onto a maximum parsimony phylogenetic tree of representative 16S sequences targeted by the array to examine the phylogenetic distribution of these traits. To statistically test the phylogenetic distribution of trophic strategy on the tree, individual characters states were randomly reshuffled 1000 times, and for each reshuffling, a parsimony score (number of character state changes) was calculated. This was carried out with the software package Mesquite [23]. The parsimony score of the real character distribution was compared to this null distribution with an F-Test.

## Results and Discussion

### Establishment of Discrete Categories of Functional Responses to Resource Availability

**Figure 1:**
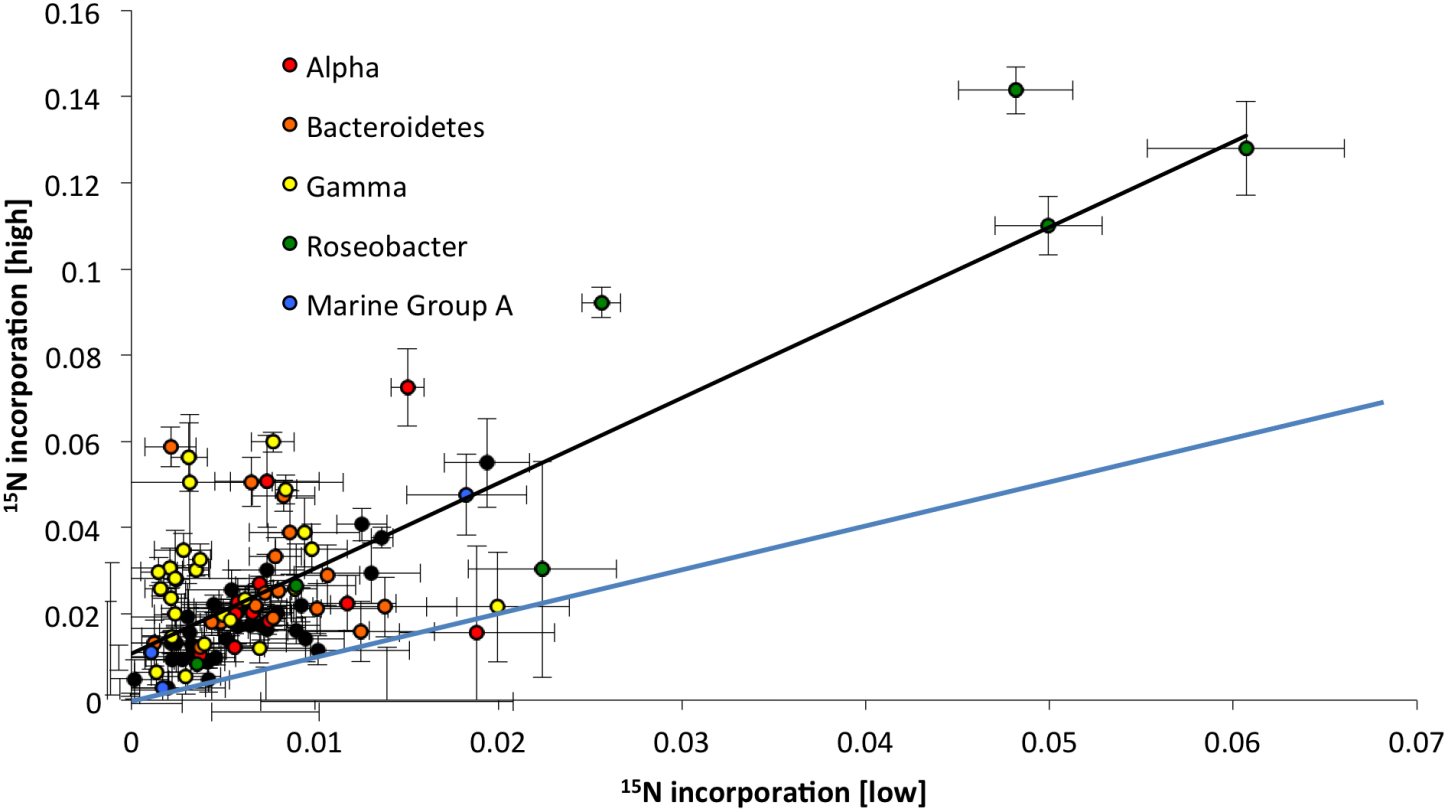
Pairwise comparisons of isotopic incorporation of ^15^N labeled AAs by 107 16S rRNA phylotypes from SF Bay at two concentrations (high, 5 micromolar and low, 50 nanomolar). Each data point represents the HCE (hybridization corrected enrichment) for a probe set (the slope of delta permil divided by fluorescence). Error bars indicate two standard errors of the slope calculation. The black line represents the linear regression and the blue the 1 to l line.

In our initial analysis, we restricted our examination of the data to pairwise comparisons of two concentrations to start developing a classification scheme for microbial response to varying substrate concentrations (Fig. 1). Generally, incorporation at high substrate concentrations was weakly but significantly correlated to incorporation at low concentration, meaning that rRNA phylotypes with high activity at the high substrate concentration also had high activity at the low concentration (and vice-versa). This is exemplified by statistically significant linear regression analyses (e.g. R^2^ = 0.62, p<0.0001 for H vs. L; black line in Fig. 1). This initial analysis delineated two groups of organisms: the first exhibited high isotopic incorporation (i.e. high activity), and the other group lower isotopic incorporation (i.e. lower activity). We considered taxa to exhibit high activity if their measure of isotopic incorporation was more than 50% of the highest incorporation recorded for a given concentration (Fig. S1). In this set of experiments, all highly active taxa (4) were members of the *Rhodobacteriaceae*, represented by probe sets targeting 3 taxa and the family more broadly. These marine bacteria belong to a subclade of the Alpha Proteobacteria, play key biogeochemical roles in the ocean [24] and are believed to be substrate generalists [25]. We note that relatively few taxa were classified as highly active (4 out of 107) based on our 50% cutoff criterion, in agreement with the theory that only a few members of a microbial community are very active at any one time [26]. It is unlikely that this result was caused by bottle effects [27] or selection of taxa during batch culture incubations [28], as previous work has shown that marine communities incubated in bottles for extended periods become dominated by *Alteromonadales* [29], not *Rhodobacteriaceae*.

**Figure 2:**
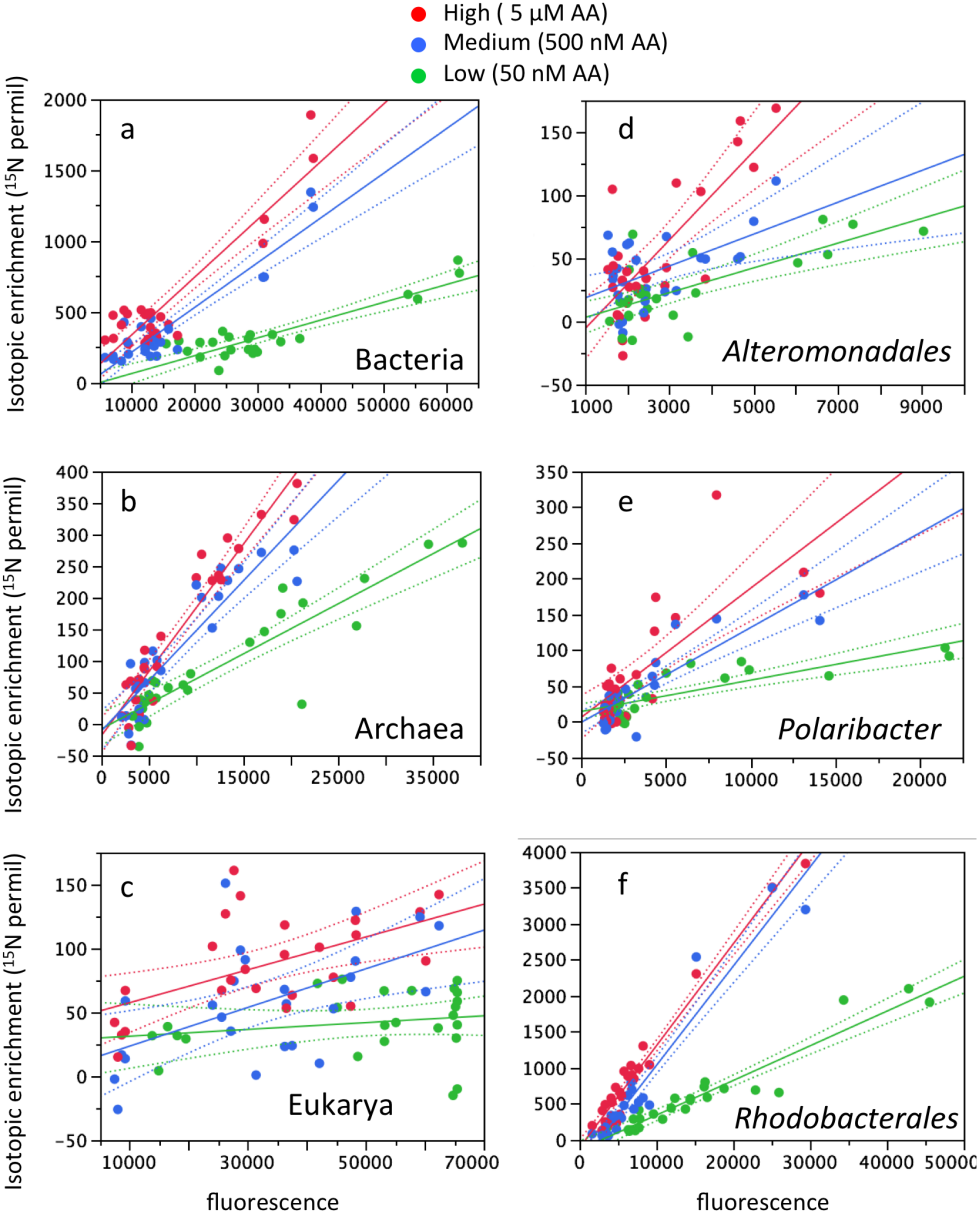
Response of taxa targeted by domain-specific (a–c) and genus or order specific (d–f) probes to increasing amino acid concentrations (red = high, blue = medium, green = low). Data points are for individual probes. Solid lines represent the linear regression and dotted lines are 95% confidence intervals.

The response of the domain-specific probes indicated that Bacteria and Archaea increased their AA incorporation with increasing concentrations (Fig. 2a, 2b). In other words, as the concentration of added labeled substrate increased, isotopic incorporation increased. Eukaryotic AA incorporation was near the detection limit at the low concentration (and not considered significantly enriched by our conservative criteria) and was positive at the medium and high concentrations (Fig. 2c). The data for the three probe sets for bacterial orders/genus (*Alteromonadales*, *Rhodobacterales*, *Polaribacters*) allowed us to begin to split the bacterial response detected at the domain level into more specific components. These data revealed that the probes for these lower phylogenetic groups did not necessarily respond similarly to the Bacteria-specific probe set. *Alteromonadales* increased their isotopic incorporation with increasing substrate concentration (Fig. 2d), as the general Bacterial domain probes did. However, *Rhodobacterales* and *Polaribacters* showed no significant increase in isotopic incorporation from the medium to the high concentration treatment (Fig. 2e, 2f).

**Figure 3:**
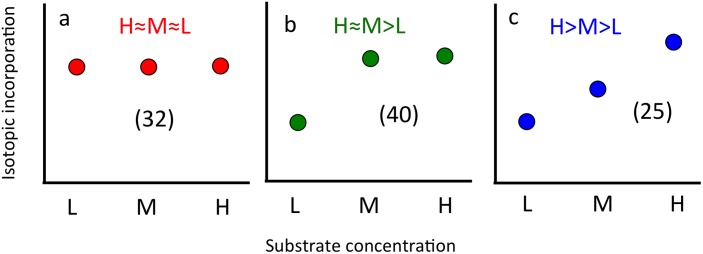
Three different types of microbial responses to resource availability identified by measuring isotopic incorporation of amino acids at High, Medium, and Low concentrations in SF Bay, with the numbers of taxa identified in parentheses.

In general, the taxon-specific isotope incorporation responses to increasing AA availability spanned a continuous range, from organisms that exhibited no increased incorporation with increasing concentrations, to those with increased incorporation as AA availability increased. For the sake of conceptual understanding, we split this continuum into discrete categories, which were identified based on statistical tests of significantly different isotopic incorporation for the three AA concentrations. The maximum number of potential categories was inherently limited by the number of substrate concentrations tested. With a greater number of AA concentrations, we likely would have identified more response categories. The statistical tests that we carried out led to the classification of three main responses to increasing substrate concentrations. First, about one third of the taxa (32/107) exhibited no difference in isotopic incorporation among the three concentrations (Fig. 3a). We classified this strategy as “H≈M≈L” (high≈medium≈low). In some of these cases, isotopic incorporation was very low (near our detection limit) due to low activity by those taxa, so our method likely could not have detected differential incorporation even if it were occurring. However, in other cases, activity was substantially above background and also did not increase with increased substrate availability. Our interpretation of this phenomenon is that the microbial populations were saturated at the lower concentration, and adding more substrate did not increase incorporation. Another non-mutually exclusive possibility is that another nutrient was limiting to their growth, although this is less likely in a nutrient-rich eutrophic ecosystem such as San Francisco Bay. We interpret this strategy (H≈M≈L) to be analogous to oligotrophy, keeping in mind that trophic strategy is a relative measure. In other words, an organism showing this response is only an oligotroph when compared to another with a different response, and this would change depending on the ecosystem, the substrate, and the concentrations tested. For example, a copiotroph in our sampled eutrophic estuary would be considered an oligotroph relative to *E. coli* growing in the laboratory, and an oligotroph in SF Bay might be considered a copiotroph compared to a very slow growing microbe in the open ocean.

The second identified trophic strategy included 40 phylotypes that exhibited increased incorporation at the medium (M) concentration compared to the low (L) but then were saturated or limited by another nutrient (H≈M>L, Figs. 3b). Physiologically, this indicates that AA incorporation was not saturated at the two lower concentrations but only at the highest concentration. We consider these taxa to be of intermediate trophy, able to respond to additions of 500 nM AAs, as compared to the relatively oligotrophic H≈M≈L strategy. The third identified group included 25 phylotypes with increased incorporation as more substrates were added (H>M>L, Figs. 3c). These organisms were not saturated at any of the concentrations tested, and we consider them to be relatively copiotrophic, able to respond to additions of 500 nM and 5 µM AAs. Since in general, additions of AAs to seawater lead to increased bacterial growth rates [30], one of our hypotheses was that highly active taxa (i.e., those with high AA incorporation) would be copiotrophs (H>M>L). We found this to be the case for two out of the three *Rhodobacteriaceae* taxa, while the other taxon and the *Rhodobacteriaceae* family exhibited intermediate trophy (H≈M>L). We originally assumed that metabolic activity and the ability to incorporate increased amounts of resources might be related, because fast-growing organisms might be expected to outgrow their competitors if they are able to incorporate resources when they are in excess. Thus we hypothesized that taxa with high AA incorporation would also be able to increase their incorporation as AA concentrations increased. This hypothesis was not fully supported by the data since not all high activity taxa were categorized as H>M>L (though many of them were).

**Figure 4:**
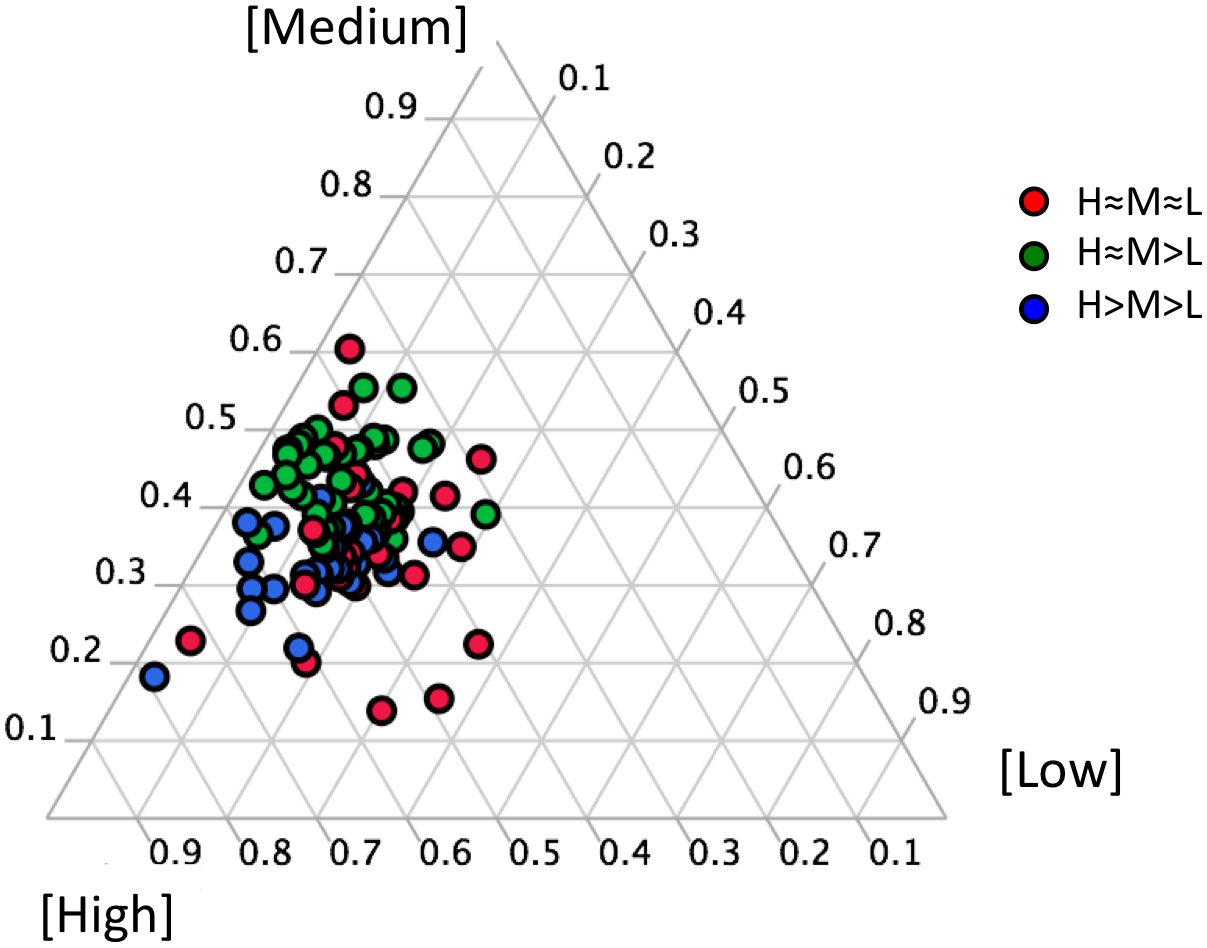
Ternary plot graphically depicting the ratios of the rRNA phylotype-specific incorporation to varying AA concentrations added to SF Bay water. Data are color-coded according to the trophic strategies identified in Fig. 3. The position of each data point in relation to the three corners represents the relative contribution of each concentration response.

After assigning taxa to a trophic strategy as outlined above, we plotted the isotopic incorporation for the three tested AA concentrations on a ternary diagram (Fig. 4). This plot graphically depicts the ratios of three variables on an equilateral triangle. In this case, the variables are the HCE values (a measure of isotopic incorporation) under 5 µM, 500 nM, and 50 nM added AAs, and for each phylotype, the denominator is the sum of the three responses. We note that activity level (i.e., active vs. less active) is not taken into account here, but only the response to substrate concentrations (H≈M>L, etc.). Data on ternary plots were color-coded according to the defined trophic strategies as described above. The ternary plots allowed the assigned categories to be visualized, and demonstrated a clear graphical partition of H≈M>L and H>M>L, while the null hypothesis H≈M≈L was less clearly distinct.

### Is Trophic Strategy Related to Phylogeny?

**Figure 5:**
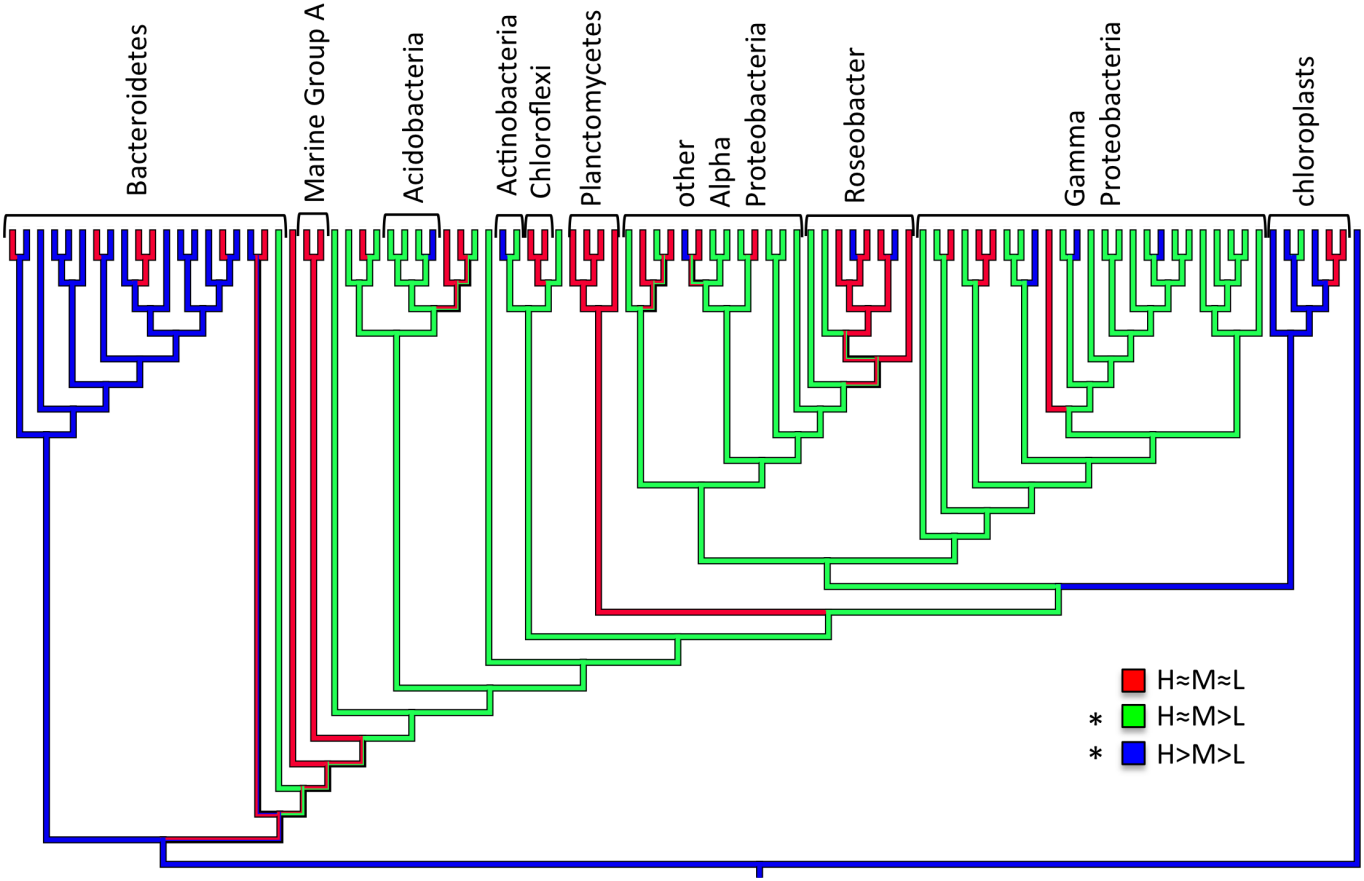
Amino acid incorporation trophic strategies mapped onto a maximum parsimony unrooted 16S rRNA gene phylogeny of taxa from a SF Bay seawater sample. Ancestral states were identified by parsimony. Asterisks indicate strategies with a statistically clustered distribution indicating a phylogenetic signal.

A common assumption in microbial ecology, although controversial [31], is that evolutionarily-related organisms share some physiological attributes with one another. To examine if such a pattern existed here, we color-coded the ternary plots according to bacterial taxonomy (not shown). No obvious taxonomic pattern was evident based on a visual examination of this plot. We also statistically tested the hypothesis that trophic strategy was related to phylogeny by mapping the identified trophic guilds onto a phylogenetic tree of the 16S rRNA gene (Fig. 5). We determined whether the three trophic strategies were statistically restricted to certain parts of the phylogeny using a reshuffling analysis. Two of the trophic strategies (H≈M>L and H>M>L) showed a statistically significant difference from the random distribution (Fig. S2), meaning that they were phylogenetically clustered. This implies that these strategies may be under positive selective pressure in this ecosystem, an environment rich in nutrients and particles [32], [33]. In terms of taxonomic information, the intermediate strategy (H≈M>L) was more frequent in the Gamma Proteobacteria while the more copiotrophic strategy (H>M>L) was more frequent within the Bacteroidetes and chloroplasts (the latter is a marker for phototrophic eukaryotes). This is consistent with previous findings that Bacteroidetes are numerically enriched during algal blooms, when organic matter is in high supply [34], [35]. The finding that eukaryotes were copiotrophic for AA incorporation was more unexpected. It is known that eukaryotes incorporate dissolved organic nitrogen [36], [37], and previous work has shown copiotrophy by larger size fractions (mostly eukaryotes) for ammonium and phosphate [3]. Taken together, these previous observations are consistent with our data. More generally, the finding of some correlation between trophic strategy and 16S phylogeny is noteworthy, as it suggests that the substrate affinities of microbial transporters (at least those for AAs) are evolutionarily conserved and results in evolutionarily related organisms having similar responses to increasing substrate concentrations.

### Increased Categorization Enhances Conceptual Interpretation of Microbial Resource Utilization

One of the outcomes of our study is an increased number of categories of microbial resource acquisition strategies. Categorizing organisms with similar functional traits into more resource acquisition strategies allows us to strengthen our conceptual understanding of the processes they mediate, such as C and N cycling. However, the number of boxes needs to be relatively low or we lose our ability to use them conceptually. For example, we can easily conceptualize microbes based on their temperature tolerance, such as thermophiles, mesophiles, and psychrophiles [38], being adapted to high, medium, and low temperature, respectively. However, it is difficult to conceptualize ten or twenty such groups (e.g. those that prefer 70–80°C, 60–70°C, 50–60°C, etc.). Our analysis resulted in three trophic strategy guilds and two activity guilds, which allows us to retain a conceptual understanding of these functional categories without the high number of categories being too detailed to be useful.

The results presented here show that in addition to spatial and resource partitioning [39], microbial niche differentiation based on substrate availability is a factor that contributes to the maintenance of diversity in aquatic environments. In a eutrophic ecosystem, with three concentrations of one type of organic substrate, we defined more complex substrate acquisition strategies than the previously identified dichotomy of oligotrophy vs. copiotrophy. We note that although AAs are incorporated by most microbial groups, previous work has shown that assimilation of individual AAs can be quite variable and some taxa do not incorporate amino acids at detectable levels [40]. Since our experiments used a mixture of AAs, it is conceivable that our guild classifications might have been different if individual AAs were tested. The classifications would also potentially be different with other substrates, at different concentrations, during different times of the year, or in other ecosystems. Hence, the conclusions presented here are not meant to represent an absolute classification of these organisms into trophic guilds but instead to demonstrate that the well-documented concepts of oligotrophy and copiotrophy are simplifications of a continuum of responses, and perhaps more importantly, implies a relationship between activity and trophic strategy that is not universal.

The guilds identified here, which were based on both activity and response to increased substrate concentration, are critical microbial adaptations to an environment with constantly changing resource availability such as an estuary. Our analysis demonstrates that the complex substrate incorporation patterns of natural mixed microbial communities can be quantified and categorized in moderately simple classification schemes, leading to an improvement in how microbial populations are assigned to functional guilds. The categorization of microbes into an increasing number of “boxes” could be particularly valuable for biogeochemical models, where the vast diversity of bacterial heterotrophic processes [41] are typically represented in only one or two boxes. Expanding the bacterial “black box” (i.e., increasing the number of boxes to more accurately reflect the diversity of microbial responses to changing resources) should increase the accuracy and usefulness of such models. Furthermore, our quantitative approach would eventually allow these boxes to be dropped for a quantitative modeling approach, no longer needing discrete categorization. In particular, knowledge about microbial response to varying nutrient concentrations would be useful, as these are expected to change under a variety of climate change scenarios [42]. Together with new approaches to chemical characterization of organic matter complexity [43], the types of data shown here offer the possibility of testing hypotheses about critical microbial biogeochemical function. Our results quantifying the phylotype-specific response of microbial AA incorporation with increasing concentrations represents only the beginning of what we hope are many future experiments. For example, do microbial taxa exhibit the same resource use strategies for different substrates? Do taxa change their resource use strategies over time, space, or under different environmental conditions? It is clear that many of these questions must be answered before we have enough functional understanding of microbial biogeochemistry to be able to predict ecosystem-level responses to changing environmental conditions driven by both natural and anthropogenic forces.

## Supporting Information

Figure S1 (a) Hybridization Corrected Enrichment (HCE) values and (b) percent change from one value to the next lowest, for 107 taxa at the high amino acid concentration, ranked from high to low. Arrow indicates the cutoff used to delineate highly active versus less active tax, which corresponds to 50% of the maximum.
(TIF)

Figure S2 Result of phylogenetic distribution of taxa assigned to the “H≈M>L” strategy (a) and the “H>M>L” strategy (b) on the 16S phylogeny, showing the frequency distribution of parsimony scores from 1000 randomly shuffled character states. The parsimony score for the actual dataset is indicated by the arrow and was significantly different from the null distribution based on an F-test, indicating a phylogenetic signal.
(TIF)

Figure S3 Ribosomal Operational Taxonomic Units (OTUs) targeted by Chip-SIP phylogenetic microarray, including Genbank accession numbers of representative sequences, taxonomy, trophic strategy identified by amino acid incorporation at 3 concentrations, and heat map measures of relative isotopic incorporation (blue = low, black = medium, and yellow = high). *denotes highly active taxa as defined in Figure 1.
(TIF)

Table S1 List of probes specific for San Francisco Bay natural community used for Chip-SIP analyses.
(DOCX)

## References

[1] Whitman WB, Coleman DC, Wiebe WJ (1998) Prokaryotes: the unseen majority. Proc Natl Acad Sci USA 95: 6578–6583. 9618454 10.1073/pnas.95.12.6578 PMC33863

[2] Polz MF, Hunt DE, Preheim SP, Weinreich DM (2006) Patterns and mechanisms of genetic and phenotypic differentiation in marine microbes. Phil Trans R Soc B 361: 2009–2021. 17062417 10.1098/rstb.2006.1928 PMC1764928

[3] Suttle C, Fuhrman JA, Capone DG (1990) Rapid ammonium cycling and concentration-dependent partitioning of ammonium and phosphate: implications for carbon transfer in planktonic communities. Limnol Oceanogr 35: 424–433.

[4] Martens-Habbena W, Berube PM, Urakawa H, de la TorreJR, Stahl DA (2009) Ammonia oxidation kinetics determine niche separation of nitrifying Archaea and Bacteria. Nature 461: 976–979. 19794413 10.1038/nature08465

[5] Alonso C, Pernthaler J (2006) Concentration-dependent patterns of leucine incorporation by coastal picoplankton. Appl Environ Microbiol 72: 2141–2147. 16517664 10.1128/AEM.72.3.2141-2147.2006 PMC1393217

[6] Azam F (1998) Microbial control of oceanic carbon flux: the plot thickens. Science 280: 694–696.

[7] Nissen H, Nissen P, Azam F (1984) Multiphasic uptake of D-glucose by an oligotrophic marine bacterium. Mar Ecol Prog Ser 16: 155–160.

[8] Stocker R, Seymour JR, Samadani A, Hunt DE, Polz MF (2008) Rapid chemotactic response enables marine bacteria to exploit ephemeral microscale nutrient patches. Proc Natl Acad Sci USA 105: 4209–4214. 18337491 10.1073/pnas.0709765105 PMC2393791

[9] Koch AL (2001) Oligotrophs versus copiotrophs. BioEssays 23: 657–661. 11462219 10.1002/bies.1091

[10] Morris RM, Rappe MS, Connon SA, Vergin KL, Siebold WA, et al (2002) SAR11 clade dominates ocean surface bacterioplankton communities. Nature 420: 806–810. 12490947 10.1038/nature01240

[11] Lauro FM, McDougald D, Thomas T, Williams TJ, Egan S, et al (2009) The genomic basis of trophic strategy in marine bacteria. Proc Natl Acad Sci USA 106: 15527–15533. 19805210 10.1073/pnas.0903507106 PMC2739866

[12] Fuhrman JA, Azam F (1980) Bacterioplankton secondary production estimates for coastal waters of British Columbia, Canada, Antarctica, and California, USA. Appl Environ Microbiol 39: 1085–1095. 16345577 10.1128/aem.39.6.1085-1095.1980 PMC291487

[13] Poretsky RS, Sun S, Mou X, Moran MA (2010) Transporter genes expressed by coastal bacterioplankton in response to dissolved organic carbon. Envir Microbiol 12: 616–627. 10.1111/j.1462-2920.2009.02102.x PMC2847192 19930445

[14] Jørgensen NOG, Kroer N, Coffin RB, Yang X-H, Lee C (1993) Dissolved free amino acids, combined amino acids, and DNA as sources of carbon and nitrogen to marine bacteria. Mar Ecol Prog Ser 98: 135–148.

[15] Coffin RB (1989) Bacterial uptake of dissolved free and combined amino acids in estuarine water. Limnol Oceanogr 34: 531–542.

[16] Giovannoni SJ, Tripp HJ, Givan S, Podar M, Vergin KL, et al (2005) Genome streamlining in a cosmopolitan oceanic bacterium. Science 309: 1242–1245. 16109880 10.1126/science.1114057

[17] Yooseph S, Nealson KH, Rusch DB, McCrow JP, Dupont CL, et al (2010) Genomic and functional adaptation in surface ocean planktonic prokaryotes. Nature 468: 60–66. 21048761 10.1038/nature09530

[18] Sowell SM, Abraham PE, Shah M, Verberkmoes NC, Smith DP, et al (2011) Environmental proteomics of microbial plankton in a highly productive coastal upwelling system. ISME J 5: 856–865. 21068774 10.1038/ismej.2010.168 PMC3105774

[19] Malmstrom RR, Kiene RP, Cottrell MT, Kirchman DL (2004) Contribution of SAR11 Bacteria to Dissolved Dimethylsulfoniopropionate and Amino Acid Uptake in the North Atlantic Ocean. Appl Environ Microbiol 70: 4129–4135. 15240292 10.1128/AEM.70.7.4129-4135.2004 PMC444831

[20] Alonso-Sáez L, Gasol JM (2007) Seasonal Variations in the Contributions of Different Bacterial Groups to the Uptake of Low-Molecular-Weight Compounds in Northwestern Mediterranean Coastal Waters. Appl Environ Microbiol 73: 3528–3535. 17400772 10.1128/AEM.02627-06 PMC1932672

[21] Mayali X, Weber PK, Brodie EL, Mabery S, Hoeprich P, et al (2012) High-throughput isotopic analysis of RNA microarrays to quantify microbial resource use ISME J. 6: 1210–1221. 10.1038/ismej.2011.175 PMC3358021 22158395

[22] Singh-Gasson S, Green RD, Yue Y, Nelson C, Blattner F, et al (1999) Maskless fabrication of light-directed oligonucleotide microarrays using a digital micromirror array. Nat Biotech 17: 974–978. 10.1038/13664 10504697

[23] Maddison WP, Madison DR (2011) Mesquite: a modular system for evolutionary analysis. 2.75 ed.

[24] Buchan A, Gonzàlez JM, Moran MA (2005) Overview of the marine Roseobacter lineage. Appl Environ Microbiol 71: 5665–5677. 16204474 10.1128/AEM.71.10.5665-5677.2005 PMC1265941

[25] Newton RJ, Griffin LE, Bowles KM, Meile C, Gifford S, et al (2010) Genome characteristics of a generalist marine bacterial lineage. ISME J 4: 784–798. 20072162 10.1038/ismej.2009.150

[26] Smith EM, del Giorgio PA (2003) Low fractions of active bacteria in natural aquatic communities? Aquat Microb Ecol 31: 203–208.

[27] Hammes F, Vital M, Egli T (2010) Critical evaluation of the volumetric “bottle effect” on microbial batch growth. Appl Environ Microbiol 76: 1278–1281. 20023110 10.1128/AEM.01914-09 PMC2820953

[28] Eilers H, Pernthaler J, Amann R (2000) Succession of Pelagic Marine Bacteria during Enrichment: a Close Look at Cultivation-Induced Shifts. Appl Environ Microbiol 66: 4634–4640. 11055904 10.1128/aem.66.11.4634-4640.2000 PMC92360

[29] Schafer H, Servais P, Muyzer G (2000) Successional changes in the genetic diversity of a marine bacterial assemblage during confinement. Arch Microbiol 173: 138–145. 10795685 10.1007/s002039900121

[30] Kirchman DL, Rich JH (1997) Regulation of Bacterial Growth Rates by Dissolved Organic Carbon and Temperature in the Equatorial Pacific Ocean. Microb Ecol 33: 11–20. 9039761 10.1007/s002489900003

[31] Doolittle WF, Zhaxybayeva O (2009) On the origin of prokaryotic species. Genome Res 19: 744–756. 19411599 10.1101/gr.086645.108

[32] Kranck K, Milligan TG (1992) Characteristics of suspended particles at an 11-hour anchor station in San Francisco Bay, California. Journal of Geophysical Research: Oceans 97: 11373–11382.

[33] Hollibaugh JT, Wong PS, Murrell MC (2000) Similarity of particle-associated and free-living bacterial communities in northern San Francisco Bay, California. Aquat Microb Ecol 21: 103–114.

[34] Kirchman DL (2002) The ecology of Cytophaga-Flavobacteria in aquatic environments. FEMS Microbiol Ecol 39: 91–100. 19709188 10.1111/j.1574-6941.2002.tb00910.x

[35] Teeling H, Fuchs BM, Becher D, Klockow C, Gardebrecht A, et al (2012) Substrate-controlled succession of marine bacterioplankton populations induced by a phytoplankton bloom. Science 336: 608–611. 22556258 10.1126/science.1218344

[36] Bronk DA, See JH, Bradley P, Killberg L (2007) DON as a source of bioavailable nitrogen for phytoplankton. Biogeosciences 4: 283–296.

[37] Mulholland MR, Lee C, Glibert PM (2003) Extracellular enzyme activity and uptake of carbon and nitrogen along an estuarine salinity and nutrient gradient. Mar Ecol Prog Ser 258: 3–17.

[38] Ratkowsky DA, Olley J, McMeekin TA, Ball A (1982) Relationship between temperature and growth rate of bacterial cultures. J Bacteriol 149: 1–5. 7054139 10.1128/jb.149.1.1-5.1982 PMC216584

[39] Hunt DE, David LA, Gevers D, Preheim SP, Alm EJ, et al (2008) Resource partitioning and sympatric differentiation among closely related bacterioplankton. Science 320: 1081–1085. 18497299 10.1126/science.1157890

[40] Salcher MM, Posch T, Pernthaler J (2013) In situ substrate preferences of abundant bacterioplankton populations in a prealpine freshwater lake. ISME J 7: 896–907. 23235289 10.1038/ismej.2012.162 PMC3635242

[41] Eichinger M, Poggiale JC, Sempéré R (2011) Toward a mechanistic approach to modeling bacterial DOC pathways: a review. In: Jiao N, Azam F, editors. Microbial carbon pump in the ocean. Washington D.C.: Science/AAAS.

[42] Harley CDG, Randall Hughes A, Hultgren KM, Miner BG, Sorte CJB, et al (2006) The impacts of climate change in coastal marine systems. Ecol Lett 9: 228–241. 16958887 10.1111/j.1461-0248.2005.00871.x

[43] Kujawinski EB (2011) The impact of microbial metabolism on marine dissolved organic matter. Ann Rev Mar Sci 3: 567–599. 10.1146/annurev-marine-120308-081003 21329217

