# Supplementary figures and images for: Phylogenetic Patterns in the Microbial Response to Resource Availability: Amino Acid Incorporation in San Francisco Bay
Source: PLoS One. 2014 Apr 21;9(4):e95842. doi: 10.1371/journal.pone.0095842 (PMC3994146; doi:10.1371/journal.pone.0095842)

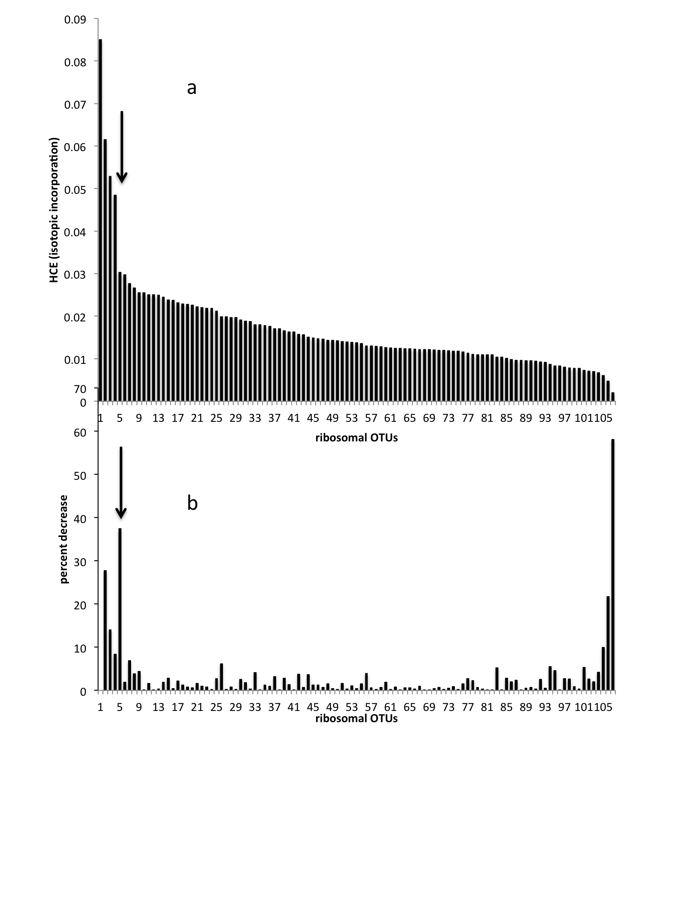

Supplement: Figure S1 — (a) Hybridization Corrected Enrichment (HCE) values and (b) percent change from one value to the next lowest, for 107 taxa at the high amino acid concentration, ranked from high to low. Arrow indicates the cutoff used to delineate highly active versus less active tax, which corresponds to 50% of the maximum. (TIF) [file pone.0095842.s001.tif]

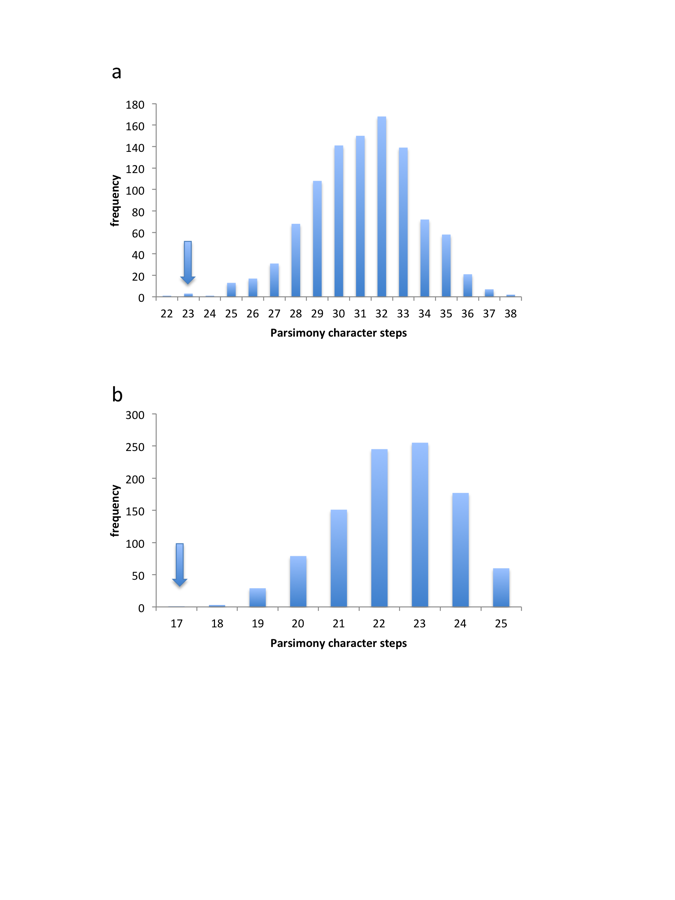

Supplement: Figure S2 — Result of phylogenetic distribution of taxa assigned to the “H≈M>L” strategy (a) and the “H>M>L” strategy (b) on the 16S phylogeny, showing the frequency distribution of parsimony scores from 1000 randomly shuffled character states. The parsimony score for the actual dataset is indicated by the arrow and was significantly different from the null distribution based on an F-test, indicating a phylogenetic signal. (TIF) [file pone.0095842.s002.tif]

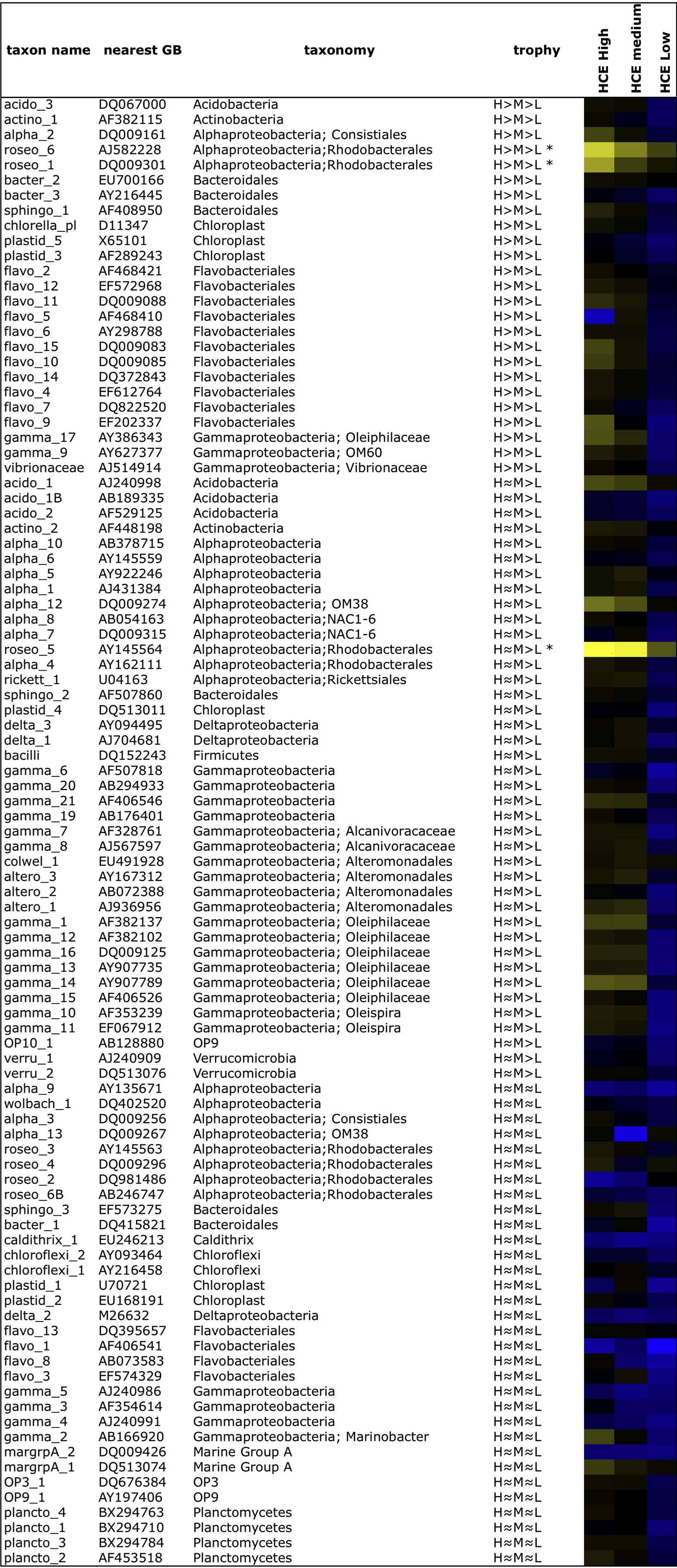

Supplement: Figure S3 — Ribosomal Operational Taxonomic Units (OTUs) targeted by Chip-SIP phylogenetic microarray, including Genbank accession numbers of representative sequences, taxonomy, trophic strategy identified by amino acid incorporation at 3 concentrations, and heat map measures of relative isotopic incorporation (blue = low, black = medium, and yellow = high). *denotes highly active taxa as defined in Figure 1. (TIF) [file pone.0095842.s003.tif]
