# Supplementary material for: Phylogenetic Patterns in the Microbial Response to Resource Availability: Amino Acid Incorporation in San Francisco Bay
Source: PLoS One. 2014 Apr 21;9(4):e95842. doi: 10.1371/journal.pone.0095842 (PMC3994146; doi:10.1371/journal.pone.0095842)
Supplement: Table S1 — List of probes specific for San Francisco Bay natural community used for Chip-SIP analyses. (DOCX) [file pone.0095842.s004.docx]

| SEQUENCE_ID | PROBE_SEQUENCE | SEQUENCE_ID | PROBE_SEQUENCE | SEQUENCE_ID | PROBE_SEQUENCE |
| --- | --- | --- | --- | --- | --- |
| eukaryotes_1 | AACTAAGAACGGCCATGCACCACCA | sphingo_1_1 | CCAGCTTGCTGCCCTCTGTACCATC | alpha_7_1 | ACATCTCTGTTTCCGCGACCGGGAT |
| eukaryotes_2 | CACCAACTAAGAACGGCCATGCACC | sphingo_1_2 | CAGCTTGCTGCCCTCTGTACCATCC | alpha_7_2 | CATCTCTGTTTCCGCGACCGGGATG |
| eukaryotes_3 | CCAACTAAGAACGGCCATGCACCAC | sphingo_1_3 | GCCAGCTTGCTGCCCTCTGTACCAT | alpha_7_3 | AAACATCTCTGTTTCCGCGACCGGG |
| eukaryotes_4 | ACCAACTAAGAACGGCCATGCACCA | sphingo_1_4 | TGCCAGCTTGCTGCCCTCTGTACCA | alpha_7_4 | GAAACATCTCTGTTTCCGCGACCGG |
| eukaryotes_5 | CCACCAACTAAGAACGGCCATGCAC | sphingo_1_5 | CAGTTTACGACCCAGAGGGCTGTCT | alpha_7_5 | AGAAACATCTCTGTTTCCGCGACCG |
| eukaryotes_6 | TCCACCAACTAAGAACGGCCATGCA | sphingo_1_6 | AGCAGTTTACGACCCAGAGGGCTGT | alpha_7_6 | AACATCTCTGTTTCCGCGACCGGGA |
| eukaryotes_7 | CAACTAAGAACGGCCATGCACCACC | sphingo_1_7 | AAGCAGTTTACGACCCAGAGGGCTG | alpha_7_7 | ATCTCTGTTTCCGCGACCGGGATGT |
| eukaryotes_8 | CTCCACCAACTAAGAACGGCCATGC | sphingo_1_8 | GCAGTTTACGACCCAGAGGGCTGTC | alpha_7_8 | CTGCCACTGTCCACCCGAGCAAGCT |
| eukaryotes_9 | TTGGAGCTGGAATTACCGCGGCTGC | sphingo_1_9 | CCGCCTACCTCTAGTGTATTCAAGC | alpha_7_9 | CCACTGTCCACCCGAGCAAGCTCGG |
| eukaryotes_10 | TCAGGCTCCCTCTCCGGAATCGAAC | sphingo_1_10 | CATTCCGCCTACCTCTAGTGTATTC | alpha_7_10 | GCCACTGTCCACCCGAGCAAGCTCG |
| eukaryotes_11 | TCTCAGGCTCCCTCTCCGGAATCGA | sphingo_1_11 | TGCTGTTGCCAGCTTGCTGCCCTCT | alpha_7_11 | AAACCTCTAGGTAGATACCCACGCG |
| eukaryotes_12 | TATTGGAGCTGGAATTACCGCGGCT | sphingo_1_12 | GCTGTTGCCAGCTTGCTGCCCTCTG | alpha_7_12 | CCAAACCTCTAGGTAGATACCCACG |
| eukaryotes_13 | ATTGGAGCTGGAATTACCGCGGCTG | sphingo_1_13 | TTGCTGTTGCCAGCTTGCTGCCCTC | alpha_7_13 | GTCTGCCACTGTCCACCCGAGCAAG |
| eukaryotes_14 | TAAGAACGGCCATGCACCACCACCC | sphingo_1_14 | CACATTCCGCCTACCTCTAGTGTAT | alpha_7_14 | CCACCCGAGCAAGCTCGGGTTTCTC |
| eukaryotes_15 | CTAAGAACGGCCATGCACCACCACC | sphingo_1_15 | GTCACATTCCGCCTACCTCTAGTGT | alpha_7_15 | TGCCACTGTCCACCCGAGCAAGCTC |
| eukaryotes_16 | ACTAAGAACGGCCATGCACCACCAC | sphingo_1_16 | TCACATTCCGCCTACCTCTAGTGTA | alpha_7_16 | CAAACCTCTAGGTAGATACCCACGC |
| eukaryotes_17 | CTCAGGCTCCCTCTCCGGAATCGAA | sphingo_1_17 | GCTTTCGCTTAGCCGCTAACTGTGT | alpha_7_17 | TCTGCCACTGTCCACCCGAGCAAGC |
| eukaryotes_18 | CTATTGGAGCTGGAATTACCGCGGC | sphingo_1_18 | CGCTTTCGCTTAGCCGCTAACTGTG | alpha_7_18 | CGTCTGCCACTGTCCACCCGAGCAA |
| eukaryotes_19 | AAGAACGGCCATGCACCACCACCCA | sphingo_1_19 | TCGCTTAGCCGCTAACTGTGTATCG | alpha_7_19 | TCCGAACCTCTAGGTAGATTCCCAC |
| eukaryotes_20 | AGGCTCCCTCTCCGGAATCGAACCC | sphingo_1_20 | TTCGCTTAGCCGCTAACTGTGTATC | alpha_7_20 | CACCCGAGCAAGCTCGGGTTTCTCG |
| eukaryotes_21 | CAGGCTCCCTCTCCGGAATCGAACC | sphingo_1_21 | CTTTCGCTTAGCCGCTAACTGTGTA | alpha_7_21 | ACCCGAGCAAGCTCGGGTTTCTCGT |
| eukaryotes_22 | GCTATTGGAGCTGGAATTACCGCGG | sphingo_1_22 | CTGTTGCCAGCTTGCTGCCCTCTGT | alpha_7_22 | CCGTCTGCCACTGTCCACCCGAGCA |
| eukaryotes_23 | TTTCTCAGGCTCCCTCTCCGGAATC | sphingo_1_23 | GTTGCCAGCTTGCTGCCCTCTGTAC | alpha_7_23 | CCGAACCTCTAGGTAGATTCCCACG |
| eukaryotes_24 | GGCTCCCTCTCCGGAATCGAACCCT | sphingo_1_24 | TGTTGCCAGCTTGCTGCCCTCTGTA | alpha_7_24 | AACCTCTAGGTAGATACCCACGCGT |
| eukaryotes_25 | CACTCCACCAACTAAGAACGGCCAT | sphingo_1_25 | CGCTTAGCCGCTAACTGTGTATCGC | alpha_7_25 | TCCACCCGAGCAAGCTCGGGTTTCT |
| archaea_1 | TTGTGGTGCTCCCCCGCCAATTCCT | sphingo_2_1 | TCACCGCTACACCCCTCGTTCCGCT | alpha_8_1 | CTGCCACTGTCCACCCGAGCAAGCT |
| archaea_2 | TGCTCCCCCGCCAATTCCTTTAAGT | sphingo_2_2 | GCTATCGGCGTTCTGAGGAATATCT | alpha_8_2 | GCCACTGTCCACCCGAGCAAGCTCG |
| archaea_3 | CGCGCCTGCTGCGCCCCGTAGGGCC | sphingo_2_3 | CGCTATCGGCGTTCTGAGGAATATC | alpha_8_3 | AAACCTCTAGGTAGATACCCACGCG |
| archaea_4 | TTTCGCGCCTGCTGCGCCCCGTAGG | sphingo_2_4 | TCGGCGTTCTGAGGAATATCTATGC | alpha_8_4 | GTCTGCCACTGTCCACCCGAGCAAG |
| archaea_5 | TCGCGCCTGCTGCGCCCCGTAGGGC | sphingo_2_5 | TTCACCGCTACACCCCTCGTTCCGC | alpha_8_5 | CCACCCGAGCAAGCTCGGGTTTCTC |
| archaea_6 | TTCGCGCCTGCTGCGCCCCGTAGGG | sphingo_2_6 | TTTCACCGCTACACCCCTCGTTCCG | alpha_8_6 | TGCCACTGTCCACCCGAGCAAGCTC |
| archaea_7 | GTGCTCCCCCGCCAATTCCTTTAAG | sphingo_2_7 | TCGCTTTCGCTTAGCCACTTACTGT | alpha_8_7 | CAAACCTCTAGGTAGATACCCACGC |
| archaea_8 | GCTCCCCCGCCAATTCCTTTAAGTT | sphingo_2_8 | CGGCGTTCTGAGGAATATCTATGCA | alpha_8_8 | TCTGCCACTGTCCACCCGAGCAAGC |
| archaea_9 | GCGCCTGCTGCGCCCCGTAGGGCCT | sphingo_2_9 | AACTAATGGGGCGCATGCCCATCCC | alpha_8_9 | ACTGTCCACCCGAGCAAGCTCGGGT |
| archaea_10 | CGCCTGCTGCGCCCCGTAGGGCCTG | sphingo_2_10 | CGCTTAGCCACTTACTGTATATCGC | alpha_8_10 | CCACTGTCCACCCGAGCAAGCTCGG |
| archaea_11 | GCCTGCTGCGCCCCGTAGGGCCTGG | sphingo_2_11 | ACTAATGGGGCGCATGCCCATCCCG | alpha_8_11 | CCAAACCTCTAGGTAGATACCCACG |
| archaea_12 | GTTTCGCGCCTGCTGCGCCCCGTAG | sphingo_2_12 | GCCATGCAGCACCTCGTATAGAGTC | alpha_8_12 | GTCCACCCGAGCAAGCTCGGGTTTC |
| archaea_13 | CTTGTGGTGCTCCCCCGCCAATTCC | sphingo_2_13 | AGCCATGCAGCACCTCGTATAGAGT | alpha_8_13 | TCCACCCGAGCAAGCTCGGGTTTCT |
| archaea_14 | GGTTTCGCGCCTGCTGCGCCCCGTA | sphingo_2_14 | CAGCCATGCAGCACCTCGTATAGAG | alpha_8_14 | CGTCTGCCACTGTCCACCCGAGCAA |
| archaea_15 | AGGTTTCGCGCCTGCTGCGCCCCGT | sphingo_2_15 | ACAGCCATGCAGCACCTCGTATAGA | alpha_8_15 | TGTCCACCCGAGCAAGCTCGGGTTT |
| archaea_16 | CCTGCTGCGCCCCGTAGGGCCTGGA | sphingo_2_16 | CTTACTTGTCAGCCTACGCACCCTT | alpha_8_16 | ACCTCTAGGTAGATACCCACGCGTT |
| archaea_17 | CCTTGTGGTGCTCCCCCGCCAATTC | sphingo_2_17 | ACTTACTTGTCAGCCTACGCACCCT | alpha_8_17 | CACCCGAGCAAGCTCGGGTTTCTCG |
| archaea_18 | CCCCTTGTGGTGCTCCCCCGCCAAT | sphingo_2_18 | CCACTGACTTACTTGTCAGCCTACG | alpha_8_18 | TAAGCCGTCTGCCACTGTCCACCCG |
| archaea_19 | ACCCCTTGTGGTGCTCCCCCGCCAA | sphingo_2_19 | CACTGACTTACTTGTCAGCCTACGC | alpha_8_19 | ACCCGAGCAAGCTCGGGTTTCTCGT |
| archaea_20 | CCCTTGTGGTGCTCCCCCGCCAATT | sphingo_2_20 | GACTTACTTGTCAGCCTACGCACCC | alpha_8_20 | CCGTCTGCCACTGTCCACCCGAGCA |
| archaea_21 | CACCCCTTGTGGTGCTCCCCCGCCA | sphingo_2_21 | TGACTTACTTGTCAGCCTACGCACC | alpha_8_21 | AACCTCTAGGTAGATACCCACGCGT |
| archaea_22 | GTGTGTGCAAGGAGCAGGGACGTAT | sphingo_2_22 | CTGACTTACTTGTCAGCCTACGCAC | alpha_8_22 | GCCGTCTGCCACTGTCCACCCGAGC |
| archaea_23 | TGTGTGCAAGGAGCAGGGACGTATT | sphingo_2_23 | ACTGACTTACTTGTCAGCCTACGCA | alpha_8_23 | TAGATACCCACGCGTTACTAAGCCG |
| archaea_24 | CGGTGTGTGCAAGGAGCAGGGACGT | sphingo_2_24 | CCATGCAGCACCTCGTATAGAGTCC | alpha_8_24 | AAGCCGTCTGCCACTGTCCACCCGA |
| archaea_25 | GGTGTGTGCAAGGAGCAGGGACGTA | sphingo_2_25 | CGCTTTCGCTTAGCCACTTACTGTA | alpha_8_25 | GTAGATACCCACGCGTTACTAAGCC |
| bacteria_1 | CGCTCGTTGCGGGACTTAACCCAAC | sphingo_3_1 | AGTTTCCTCGAGCTATGCCCCAGTT | alpha_9_1 | TCTCCGGCGACCAAACTCCCCATGT |
| bacteria_2 | GCTCGTTGCGGGACTTAACCCAACA | sphingo_3_2 | CGAGTTTCCTCGAGCTATGCCCCAG | alpha_9_2 | CGTCTCCGGCGACCAAACTCCCCAT |
| bacteria_3 | GACTTAACCCAACATCTCACGACAC | sphingo_3_3 | GTTTCCTCGAGCTATGCCCCAGTTA | alpha_9_3 | GTCTCCGGCGACCAAACTCCCCATG |
| bacteria_4 | AACCCAACATCTCACGACACGAGCT | sphingo_3_4 | TTTCCTCGAGCTATGCCCCAGTTAA | alpha_9_4 | CTCCGGCGACCAAACTCCCCATGTC |
| bacteria_5 | ACTTAACCCAACATCTCACGACACG | sphingo_3_5 | GAGTTTCCTCGAGCTATGCCCCAGT | alpha_9_5 | GCCGTCTCCGGCGACCAAACTCCCC |
| bacteria_6 | TAACCCAACATCTCACGACACGAGC | sphingo_3_6 | TCGAGTTTCCTCGAGCTATGCCCCA | alpha_9_6 | TCCGGCGACCAAACTCCCCATGTCA |
| bacteria_7 | GGACTTAACCCAACATCTCACGACA | sphingo_3_7 | TTACCGAAGTAAATGCTGCCCCTCG | alpha_9_7 | CCGTCTCCGGCGACCAAACTCCCCA |
| bacteria_8 | CTTAACCCAACATCTCACGACACGA | sphingo_3_8 | GTTGCTAGCTCTACCCTAAACAGCG | alpha_9_8 | CGCCGTCTCCGGCGACCAAACTCCC |
| bacteria_9 | TTAACCCAACATCTCACGACACGAG | sphingo_3_9 | AGTTGCTAGCTCTACCCTAAACAGC | alpha_9_9 | CCGGCGACCAAACTCCCCATGTCAA |
| bacteria_10 | GGGACTTAACCCAACATCTCACGAC | sphingo_3_10 | CCATTTACCGAAGTAAATGCTGCCC | alpha_9_10 | ACGCCGTCTCCGGCGACCAAACTCC |
| bacteria_11 | ACTGCTGCCTCCCGTAGGAGTCTGG | sphingo_3_11 | CATTTACCGAAGTAAATGCTGCCCC | alpha_9_11 | GAACTGAAGGACGCCGTCTCCGGCG |
| bacteria_12 | CTCGTTGCGGGACTTAACCCAACAT | sphingo_3_12 | CGCCATTTACCGAAGTAAATGCTGC | alpha_9_12 | CGGCGACCAAACTCCCCATGTCAAG |
| bacteria_13 | CGGGACTTAACCCAACATCTCACGA | sphingo_3_13 | TTGCTAGCTCTACCCTAAACAGCGC | alpha_9_13 | GTCGGCAGCCTCCCTTACGGGTCGG |
| bacteria_14 | TCGTTGCGGGACTTAACCCAACATC | sphingo_3_14 | GCCATTTACCGAAGTAAATGCTGCC | alpha_9_14 | GGTCGGCAGCCTCCCTTACGGGTCG |
| bacteria_15 | CGTTGCGGGACTTAACCCAACATCT | sphingo_3_15 | TCCTCGAGCTATGCCCCAGTTAAAG | alpha_9_15 | TGGTCGGCAGCCTCCCTTACGGGTC |
| bacteria_16 | GTTGCGGGACTTAACCCAACATCTC | sphingo_3_16 | TTCCTCGAGCTATGCCCCAGTTAAA | alpha_9_16 | TCGGCAGCCTCCCTTACGGGTCGGC |
| bacteria_17 | TGCGGGACTTAACCCAACATCTCAC | sphingo_3_17 | CAGTTGCTAGCTCTACCCTAAACAG | alpha_9_17 | GTGGTCGGCAGCCTCCCTTACGGGT |
| bacteria_18 | TTGCGGGACTTAACCCAACATCTCA | sphingo_3_18 | TGCTAGCTCTACCCTAAACAGCGCC | alpha_9_18 | CGTGGTCGGCAGCCTCCCTTACGGG |
| bacteria_19 | CCCCACTGCTGCCTCCCGTAGGAGT | sphingo_3_19 | CCGTCAGATCCTCTCGCAAGAGTAT | alpha_9_19 | CGGCAGCCTCCCTTACGGGTCGGCG |
| bacteria_20 | GCGGGACTTAACCCAACATCTCACG | sphingo_3_20 | CTCGAGCTATGCCCCAGTTAAAGGT | alpha_9_20 | CGCACCTCAGCGTCAGATCCGGACC |
| bacteria_21 | GCGCTCGTTGCGGGACTTAACCCAA | sphingo_3_21 | CCTCGAGCTATGCCCCAGTTAAAGG | alpha_9_21 | AATCTTTCCCCCTCAGGGCTTATCC |
| bacteria_22 | TCCCCACTGCTGCCTCCCGTAGGAG | sphingo_3_22 | CCAGTTGCTAGCTCTACCCTAAACA | alpha_9_22 | CGAACTGAAGGACGCCGTCTCCGGC |
| bacteria_23 | ATTCCCCACTGCTGCCTCCCGTAGG | sphingo_3_23 | TCTCTCTGGATGTCACTCGCATTCT | alpha_9_23 | TACCCTCTTCCGATCTCTAGCCTAG |
| bacteria_24 | TTCCCCACTGCTGCCTCCCGTAGGA | sphingo_3_24 | ATCTCTCTGGATGTCACTCGCATTC | alpha_9_24 | GGCAGCCTCCCTTACGGGTCGGCGA |
| bacteria_25 | ACCCAACATCTCACGACACGAGCTG | sphingo_3_25 | CTCTCTGGATGTCACTCGCATTCTA | alpha_9_25 | GGCGACCAAACTCCCCATGTCAAGG |
| rhodobacter_1 | TCCCCAGGCGGAATGCTTAATCCGT | caldithrix_1_1 | ACTCCTCAGAGCTTCATCGCCCACG | alpha_10_1 | CGCACCTGAGCGTCAGATCTAGTCC |
| rhodobacter_2 | CTCCCCAGGCGGAATGCTTAATCCG | caldithrix_1_2 | CTCCTCAGAGCTTCATCGCCCACGC | alpha_10_2 | TCGCACCTGAGCGTCAGATCTAGTC |
| rhodobacter_3 | ACTCCCCAGGCGGAATGCTTAATCC | caldithrix_1_3 | AACAGGGCTTTACACTCCTCAGAGC | alpha_10_3 | CGTGCGCCACTCTCCAGTTCCCGAA |
| rhodobacter_4 | CCCCAGGCGGAATGCTTAATCCGTT | caldithrix_1_4 | CACTCCTCAGAGCTTCATCGCCCAC | alpha_10_4 | CCGTGCGCCACTCTCCAGTTCCCGA |
| rhodobacter_5 | CACCGCGTCATGCTGTTACGCGATT | caldithrix_1_5 | ACAGGGCTTTACACTCCTCAGAGCT | alpha_10_5 | CCCGTGCGCCACTCTCCAGTTCCCG |
| rhodobacter_6 | TCACCGCGTCATGCTGTTACGCGAT | caldithrix_1_6 | ACACTCCTCAGAGCTTCATCGCCCA | alpha_10_6 | CTGAGCGTCAGATCTAGTCCAGGTG |
| rhodobacter_7 | ATTCACCGCGTCATGCTGTTACGCG | caldithrix_1_7 | CAGGGCTTTACACTCCTCAGAGCTT | alpha_10_7 | TTCGCACCTGAGCGTCAGATCTAGT |
| rhodobacter_8 | TAGCCCAACCCGTAAGGGCCATGAG | caldithrix_1_8 | TCCTCAGAGCTTCATCGCCCACGCG | alpha_10_8 | CCAACCGTTATCCCCCACTAAGAGG |
| rhodobacter_9 | TACTCCCCAGGCGGAATGCTTAATC | caldithrix_1_9 | TACACTCCTCAGAGCTTCATCGCCC | alpha_10_9 | TCCAACCGTTATCCCCCACTAAGAG |
| rhodobacter_10 | AGCCCAACCCGTAAGGGCCATGAGG | caldithrix_1_10 | CTTCTGGCACTCCCGACTTTCATGG | alpha_10_10 | GCACCTGAGCGTCAGATCTAGTCCA |
| rhodobacter_11 | GCCCAACCCGTAAGGGCCATGAGGA | caldithrix_1_11 | TTACACTCCTCAGAGCTTCATCGCC | alpha_10_11 | CCTGAGCGTCAGATCTAGTCCAGGT |
| rhodobacter_12 | AACGTATTCACCGCGTCATGCTGTT | caldithrix_1_12 | CCTCAGAGCTTCATCGCCCACGCGG | alpha_10_12 | GTTAGCCCACCGTCTTCGGGTAAAA |
| rhodobacter_13 | TTCACCGCGTCATGCTGTTACGCGA | caldithrix_1_13 | CCTAACAGGGCTTTACACTCCTCAG | alpha_10_13 | CCACTAAGAGGTAGGTCCCCACGCG |
| rhodobacter_14 | ACCGCGTCATGCTGTTACGCGATTA | caldithrix_1_14 | AGGGCTTTACACTCCTCAGAGCTTC | alpha_10_14 | TGAGCGTCAGATCTAGTCCAGGTGG |
| rhodobacter_15 | GCGGAATGCTTAATCCGTTAGGTGT | caldithrix_1_15 | TTCTGGCACTCCCGACTTTCATGGC | alpha_10_15 | ATCCCCCACTAAGAGGTAGGTCCCC |
| rhodobacter_16 | CCAACCCGTAAGGGCCATGAGGACT | caldithrix_1_16 | TCTGGCACTCCCGACTTTCATGGCG | alpha_10_16 | GCTTTCACCCCTGACTGGCAAGACC |
| rhodobacter_17 | CCCAGGCGGAATGCTTAATCCGTTA | caldithrix_1_17 | CTCAGAGCTTCATCGCCCACGCGGC | alpha_10_17 | CAACCGTTATCCCCCACTAAGAGGT |
| rhodobacter_18 | CCCAACCCGTAAGGGCCATGAGGAC | caldithrix_1_18 | GGGCTTTACACTCCTCAGAGCTTCA | alpha_10_18 | GCGTCACCGAAATCGAAATCCCGAC |
| rhodobacter_19 | AATTCCACTCACCTCTCTCGAACTC | caldithrix_1_19 | CTCCTAACAGGGCTTTACACTCCTC | alpha_10_19 | TGCGTCACCGAAATCGAAATCCCGA |
| rhodobacter_20 | GAATTCCACTCACCTCTCTCGAACT | caldithrix_1_20 | CTGGCACTCCCGACTTTCATGGCGT | alpha_10_20 | CGTCACCGAAATCGAAATCCCGACA |
| rhodobacter_21 | TATTCACCGCGTCATGCTGTTACGC | caldithrix_1_21 | TCAGAGCTTCATCGCCCACGCGGCG | alpha_10_21 | CTGCGTCACCGAAATCGAAATCCCG |
| rhodobacter_22 | ACGTATTCACCGCGTCATGCTGTTA | caldithrix_1_22 | ACCTCTACAGCAGTCCCGAAGGAAG | alpha_10_22 | TTTCGCACCTGAGCGTCAGATCTAG |
| rhodobacter_23 | GAACGTATTCACCGCGTCATGCTGT | caldithrix_1_23 | CCCTCCTAACAGGGTTTTACACTCC | alpha_10_23 | CTTTCACCCCTGACTGGCAAGACCG |
| rhodobacter_24 | GGAATTCCACTCACCTCTCTCGAAC | caldithrix_1_24 | GGTCGAAACCTCCAACACCTAGTGC | alpha_10_24 | CTAAAAGGTTAGCCCACCGTCTTCG |
| rhodobacter_25 | GTAGCCCAACCCGTAAGGGCCATGA | caldithrix_1_25 | GTCGAAACCTCCAACACCTAGTGCC | alpha_10_25 | CCCACTAAGAGGTAGGTCCCCACGC |
| margrpA_1 | ACGAAGTTAGCCGGTGCTTTCTTGT | chloroflexi_1_1 | TCTCCGAGGAGTCGTTCCAGTTTCC | alpha_12_1 | CCGTGCGCCACTCTATAAATAGCGT |
| margrpA_2 | CACGAAGTTAGCCGGTGCTTTCTTG | chloroflexi_1_2 | CTCCGAGGAGTCGTTCCAGTTTCCC | alpha_12_2 | CCCGTGCGCCACTCTATAAATAGCG |
| margrpA_3 | GTTACTCACCCGTTCGCCAGTTTAC | chloroflexi_1_3 | ACGAATGGGTTTGACACCACCCACA | alpha_12_3 | CCAACCGTTATCCCGCAGAAAAAGG |
| margrpA_4 | TAAGGGACATACTGACTTGACATCA | chloroflexi_1_4 | CGAATGGGTTTGACACCACCCACAC | alpha_12_4 | CCCGCAGAAAAAGGCAGGTTCCCAC |
| margrpA_5 | ATAAGGGACATACTGACTTGACATC | chloroflexi_1_5 | CTCTCCGAGGAGTCGTTCCAGTTTC | alpha_12_5 | ACCGTTATCCCGCAGAAAAAGGCAG |
| margrpA_6 | AAGGGACATACTGACTTGACATCAT | chloroflexi_1_6 | TCCGAGGAGTCGTTCCAGTTTCCCT | alpha_12_6 | CAACCGTTATCCCGCAGAAAAAGGC |
| margrpA_7 | TTACTCACCCGTTCGCCAGTTTACT | chloroflexi_1_7 | GAATGGGTTTGACACCACCCACACC | alpha_12_7 | CGTTTCCAACCGTTATCCCGCAGAA |
| margrpA_8 | CGTTACTCACCCGTTCGCCAGTTTA | chloroflexi_1_8 | GCTCTCCGAGGAGTCGTTCCAGTTT | alpha_12_8 | CCGCAGAAAAAGGCAGGTTCCCACG |
| margrpA_9 | GCGTTACTCACCCGTTCGCCAGTTT | chloroflexi_1_9 | CCGAGGAGTCGTTCCAGTTTCCCTT | alpha_12_9 | CGCAGAAAAAGGCAGGTTCCCACGC |
| margrpA_10 | CGCGTTACTCACCCGTTCGCCAGTT | chloroflexi_1_10 | CGCTCTCCGAGGAGTCGTTCCAGTT | alpha_12_10 | CCGTTATCCCGCAGAAAAAGGCAGG |
| margrpA_11 | ACATACTGACTTGACATCATCCCCA | chloroflexi_1_11 | AATGGGTTTGACACCACCCACACCT | alpha_12_11 | CGTTATCCCGCAGAAAAAGGCAGGT |
| margrpA_12 | TACTGACTTGACATCATCCCCACCT | chloroflexi_1_12 | CGAGGAGTCGTTCCAGTTTCCCTTC | alpha_12_12 | ACCCGTGCGCCACTCTATAAATAGC |
| margrpA_13 | GGACATACTGACTTGACATCATCCC | chloroflexi_1_13 | AGGAGTCGTTCCAGTTTCCCTTCAC | alpha_12_13 | CACCCGTGCGCCACTCTATAAATAG |
| margrpA_14 | GACATACTGACTTGACATCATCCCC | chloroflexi_1_14 | GAGGAGTCGTTCCAGTTTCCCTTCA | alpha_12_14 | TCCCGCAGAAAAAGGCAGGTTCCCA |
| margrpA_15 | ATACTGACTTGACATCATCCCCACC | chloroflexi_1_15 | CGCTTTGCGACATGAGCGTCAGGTT | alpha_12_15 | GCAGAAAAAGGCAGGTTCCCACGCG |
| margrpA_16 | CATACTGACTTGACATCATCCCCAC | chloroflexi_1_16 | TGAGCGTCAGGTTCAATGCCAGGGT | alpha_12_16 | GGAAACCAAACTCCCCATGTCAAGG |
| margrpA_17 | AGGGACATACTGACTTGACATCATC | chloroflexi_1_17 | ACGCTTTGCGACATGAGCGTCAGGT | alpha_12_17 | CCTCCTGCAAGCAGGTTAGCTCACC |
| margrpA_18 | GGGACATACTGACTTGACATCATCC | chloroflexi_1_18 | TCCCCACGCTTTGCGACATGAGCGT | alpha_12_18 | TTTCGCGCCTCAGCGTCAAAATCGG |
| margrpA_19 | ACGCGTTACTCACCCGTTCGCCAGT | chloroflexi_1_19 | TCAGGTTCAATGCCAGGGTACCGCT | alpha_12_19 | TTCGCGCCTCAGCGTCAAAATCGGA |
| margrpA_20 | GCACGAAGTTAGCCGGTGCTTTCTT | chloroflexi_1_20 | ATCATCTCGGCCTTCACGTTCGACT | alpha_12_20 | ACTCCCCATGTCAAGGACTGGTAAG |
| margrpA_21 | GGCACGAAGTTAGCCGGTGCTTTCT | chloroflexi_1_21 | TGCGACATGAGCGTCAGGTTCAATG | alpha_12_21 | GCCTCCTGCAAGCAGGTTAGCTCAC |
| margrpA_22 | TGGCACGAAGTTAGCCGGTGCTTTC | chloroflexi_1_22 | ATGAGCGTCAGGTTCAATGCCAGGG | alpha_12_22 | CAGAAAAAGGCAGGTTCCCACGCGT |
| margrpA_23 | ACTGACTTGACATCATCCCCACCTT | chloroflexi_1_23 | CACGCTTTGCGACATGAGCGTCAGG | alpha_12_23 | TCCGGCGGACCTTTCCCCCGTAGGG |
| margrpA_24 | CTGGCACGAAGTTAGCCGGTGCTTT | chloroflexi_1_24 | CATGAGCGTCAGGTTCAATGCCAGG | alpha_12_24 | TATCCCGCAGAAAAAGGCAGGTTCC |
| margrpA_25 | ACGATTACTAGCGATTCCTGCTTCA | chloroflexi_1_25 | GTAATCATCTCGGCCTTCACGTTCG | alpha_12_25 | CCCCTCTTTCTCCGGCGGACCTTTC |
| vibrionaceae_1 | TATCCCCCACATCAGGGCAATTTCC | chloroflexi_2_1 | GGTGACTCCCCTTTCAGGTTGCTAC | alpha_13_1 | TCTAACTGTTCAAGCAGCCTGCGAG |
| vibrionaceae_2 | CGACATTACTCGCTGGCAAACAAGG | chloroflexi_2_2 | AGGTGACTCCCCTTTCAGGTTGCTA | alpha_13_2 | CTAACTGTTCAAGCAGCCTGCGAGC |
| vibrionaceae_3 | CCGACATTACTCGCTGGCAAACAAG | chloroflexi_2_3 | CCCTCCCCATTAAGCGGGGAGATTT | alpha_13_3 | TAACTGTTCAAGCAGCCTGCGAGCC |
| vibrionaceae_4 | CCCCACATCAGGGCAATTTCCTAGG | chloroflexi_2_4 | GCAAGCTTGGCTCATCGGTACCGTT | alpha_13_4 | GTCTAACTGTTCAAGCAGCCTGCGA |
| vibrionaceae_5 | CCCCCACATCAGGGCAATTTCCTAG | chloroflexi_2_5 | CTCTCCCGATGTTCCAAGCAAGCTT | alpha_13_5 | CGCTCCTCAGCGTCAGAAAATAGCC |
| vibrionaceae_6 | CCCACATCAGGGCAATTTCCTAGGC | chloroflexi_2_6 | CCCCTCCCCATTAAGCGGGGAGATT | alpha_13_6 | GCTCCTCAGCGTCAGAAAATAGCCA |
| vibrionaceae_7 | CCACATCAGGGCAATTTCCTAGGCA | chloroflexi_2_7 | TTCCAAGCAAGCTTGGCTCATCGGT | alpha_13_7 | TCGCTCCTCAGCGTCAGAAAATAGC |
| vibrionaceae_8 | TCCCCCACATCAGGGCAATTTCCTA | chloroflexi_2_8 | AGCAAGCTTGGCTCATCGGTACCGT | alpha_13_8 | CGTCTAACTGTTCAAGCAGCCTGCG |
| vibrionaceae_9 | CCCGACATTACTCGCTGGCAAACAA | chloroflexi_2_9 | ACTCTCCCGATGTTCCAAGCAAGCT | alpha_13_9 | AACTGTTCAAGCAGCCTGCGAGCCC |
| vibrionaceae_10 | ATCCCCCACATCAGGGCAATTTCCT | chloroflexi_2_10 | ACCCCTCCCCATTAAGCGGGGAGAT | alpha_13_10 | CACGTCTAACTGTTCAAGCAGCCTG |
| vibrionaceae_11 | TGGTTATCCCCCACATCAGGGCAAT | chloroflexi_2_11 | TCTCCCGATGTTCCAAGCAAGCTTG | alpha_13_11 | ACGTCTAACTGTTCAAGCAGCCTGC |
| vibrionaceae_12 | CCCCCACATCAGGGCAATTTCCCAG | chloroflexi_2_12 | CTCCCGATGTTCCAAGCAAGCTTGG | alpha_13_12 | ACTGTTCAAGCAGCCTGCGAGCCCT |
| vibrionaceae_13 | TCCCCCACATCAGGGCAATTTCCCA | chloroflexi_2_13 | AATGACCCCTCCCCATTAAGCGGGG | alpha_13_13 | CCGGGGATTTCACGTCTAACTGTTC |
| vibrionaceae_14 | CCCCACATCAGGGCAATTTCCCAGG | chloroflexi_2_14 | GAATGACCCCTCCCCATTAAGCGGG | alpha_13_14 | CTCCTCAGCGTCAGAAAATAGCCAG |
| vibrionaceae_15 | CCCACATCAGGGCAATTTCCCAGGC | chloroflexi_2_15 | GTTCCAAGCAAGCTTGGCTCATCGG | alpha_13_15 | TTCAAGCAGCCTGCGAGCCCTTTAC |
| vibrionaceae_16 | CACATCAGGGCAATTTCCCAGGCAT | chloroflexi_2_16 | CGAATGACCCCTCCCCATTAAGCGG | alpha_13_16 | TGTTCAAGCAGCCTGCGAGCCCTTT |
| vibrionaceae_17 | CCACATCAGGGCAATTTCCCAGGCA | chloroflexi_2_17 | TGTTCCAAGCAAGCTTGGCTCATCG | alpha_13_17 | CTGTTCAAGCAGCCTGCGAGCCCTT |
| vibrionaceae_18 | ATCCCCCACATCAGGGCAATTTCCC | chloroflexi_2_18 | TCGAATGACCCCTCCCCATTAAGCG | alpha_13_18 | GTTCAAGCAGCCTGCGAGCCCTTTA |
| vibrionaceae_19 | TCCCGACATTACTCGCTGGCAAACA | chloroflexi_2_19 | AAGCAAGCTTGGCTCATCGGTACCG | alpha_13_19 | CGGCATTGCTGGATCAGAGTTGCCT |
| vibrionaceae_20 | GGTTATCCCCCACATCAGGGCAATT | chloroflexi_2_20 | TGACCCCTCCCCATTAAGCGGGGAG | alpha_13_20 | GGCATTGCTGGATCAGAGTTGCCTC |
| vibrionaceae_21 | CGCAAGTTGGCCGCCCTCTGTATGC | chloroflexi_2_21 | CCACTCTCCCGATGTTCCAAGCAAG | alpha_13_21 | CGCGGCATTGCTGGATCAGAGTTGC |
| vibrionaceae_22 | GCAAGTTGGCCGCCCTCTGTATGCG | chloroflexi_2_22 | CCTCCCCATTAAGCGGGGAGATTTC | alpha_13_22 | GCATTGCTGGATCAGAGTTGCCTCC |
| vibrionaceae_23 | ATGGTTATCCCCCACATCAGGGCAA | chloroflexi_2_23 | CAAGCTTGGCTCATCGGTACCGTTC | alpha_13_23 | GCGGCATTGCTGGATCAGAGTTGCC |
| vibrionaceae_24 | ACTCGCTGGCAAACAAGGATAAGGG | chloroflexi_2_24 | CCGATGTTCCAAGCAAGCTTGGCTC | alpha_13_24 | CCCGGGGATTTCACGTCTAACTGTT |
| vibrionaceae_25 | CGCATCTGAGTGTCAGTATCTGTCC | chloroflexi_2_25 | CACTCTCCCGATGTTCCAAGCAAGC | alpha_13_25 | ACGCGGCATTGCTGGATCAGAGTTG |
| alteromonadales_1 | CCCACTTGGGCCAATCTAAAGGCGA | chlorella_pl_1 | CGCCACTCATCGCAATCTGGCAAGC | delta_1_1 | CCGAACTACGAACTGCTTTCTGGGA |
| alteromonadales_2 | ATCCCACTTGGGCCAATCTAAAGGC | chlorella_pl_2 | GCCACTCATCGCAATCTGGCAAGCC | delta_1_2 | TCCGAACTACGAACTGCTTTCTGGG |
| alteromonadales_3 | TCCCACTTGGGCCAATCTAAAGGCG | chlorella_pl_3 | CCACTCATCGCAATCTGGCAAGCCA | delta_1_3 | TTGCTGCGGCACAGCAGGGGTCAAT |
| alteromonadales_4 | CCACTTGGGCCAATCTAAAGGCGAG | chlorella_pl_4 | CACTCATCGCAATCTGGCAAGCCAA | delta_1_4 | GTTTGCTGCGGCACAGCAGGGGTCA |
| alteromonadales_5 | CACTTGGGCCAATCTAAAGGCGAGA | chlorella_pl_5 | GCAAGCCAAATTGCATGCGTACGAC | delta_1_5 | TTTGCTGCGGCACAGCAGGGGTCAA |
| alteromonadales_6 | ACTTGGGCCAATCTAAAGGCGAGAG | chlorella_pl_6 | GCCAAATTGCATGCGTACGACTTGC | delta_1_6 | TTGCCCAACGACTTCTGGTACAACC |
| alteromonadales_7 | CTTGGGCCAATCTAAAGGCGAGAGC | chlorella_pl_7 | TGGCAAGCCAAATTGCATGCGTACG | delta_1_7 | GGTTTGCCCAACGACTTCTGGTACA |
| alteromonadales_8 | CACCTCAAGGCATGTTCCCAAGCAT | chlorella_pl_8 | CTGTGTCCACTCTGGAACTTCCCCT | delta_1_8 | TCCCCGAAGGGTTTGCCCAACGACT |
| alteromonadales_9 | TGAGCGTCAGTGTTGACCCAGGTGG | chlorella_pl_9 | CCGTCCGCCACTCATCGCAATCTGG | delta_1_9 | CCCCGAAGGGTTTGCCCAACGACTT |
| alteromonadales_10 | CGAAGCCCCCTTTGGTCCGTAGACA | chlorella_pl_10 | CCGCCACTCATCGCAATCTGGCAAG | delta_1_10 | CCGAAGGGTTTGCCCAACGACTTCT |
| alteromonadales_11 | ACAGAACCGAGGTTCCGAGCTTCTA | chlorella_pl_11 | CGTCCGCCACTCATCGCAATCTGGC | delta_1_11 | CCCGAAGGGTTTGCCCAACGACTTC |
| alteromonadales_12 | CAGAACCGAGGTTCCGAGCTTCTAG | chlorella_pl_12 | CCTGTGTCCACTCTGGAACTTCCCC | delta_1_12 | CCCGGGCTTTCACACCTGACTTAAA |
| alteromonadales_13 | AGAACCGAGGTTCCGAGCTTCTAGT | chlorella_pl_13 | GTCCGCCACTCATCGCAATCTGGCA | delta_1_13 | GCTTCCTTCAGTGGTACCGTCAACA |
| alteromonadales_14 | GAAAAACAGAACCGAGGTTCCGAGC | chlorella_pl_14 | TCCGCCACTCATCGCAATCTGGCAA | delta_1_14 | AGGCGCCTGCATCCCCGAAGGGTTT |
| alteromonadales_15 | GAACCGAGGTTCCGAGCTTCTAGTA | chlorella_pl_15 | ACCTGTGTCCACTCTGGAACTTCCC | delta_1_15 | GGCGCCTGCATCCCCGAAGGGTTTG |
| alteromonadales_16 | CCGAGGTTCCGAGCTTCTAGTAGAC | chlorella_pl_16 | GGCAAGCCAAATTGCATGCGTACGA | delta_1_16 | GCGCCTGCATCCCCGAAGGGTTTGC |
| alteromonadales_17 | CGAGGTTCCGAGCTTCTAGTAGACA | chlorella_pl_17 | CTGGCAAGCCAAATTGCATGCGTAC | delta_1_17 | GCATCCCCGAAGGGTTTGCCCAACG |
| alteromonadales_18 | AACCGAGGTTCCGAGCTTCTAGTAG | chlorella_pl_18 | CCCGTCCGCCACTCATCGCAATCTG | delta_1_18 | ATCCCCGAAGGGTTTGCCCAACGAC |
| alteromonadales_19 | ACCGAGGTTCCGAGCTTCTAGTAGA | chlorella_pl_19 | CACCTGTGTCCACTCTGGAACTTCC | delta_1_19 | CATCCCCGAAGGGTTTGCCCAACGA |
| alteromonadales_20 | AACAGAACCGAGGTTCCGAGCTTCT | chlorella_pl_20 | ACCCGTCCGCCACTCATCGCAATCT | delta_1_20 | ACCTTAGGCGCCTGCATCCCCGAAG |
| alteromonadales_21 | AAACAGAACCGAGGTTCCGAGCTTC | chlorella_pl_21 | CCACCTGTGTCCACTCTGGAACTTC | delta_1_21 | CCTTAGGCGCCTGCATCCCCGAAGG |
| alteromonadales_22 | CCGAAGCCCCCTTTGGTCCGTAGAC | chlorella_pl_22 | CACCCGTCCGCCACTCATCGCAATC | delta_1_22 | TACCTTAGGCGCCTGCATCCCCGAA |
| alteromonadales_23 | GAAGCCCCCTTTGGTCCGTAGACAT | chlorella_pl_23 | TCACCCGTCCGCCACTCATCGCAAT | delta_1_23 | ATACCTTAGGCGCCTGCATCCCCGA |
| alteromonadales_24 | AAGCCCCCTTTGGTCCGTAGACATT | chlorella_pl_24 | ACCACCTGTGTCCACTCTGGAACTT | delta_1_24 | CTTAGGCGCCTGCATCCCCGAAGGG |
| alteromonadales_25 | CCACCTCAAGGCATGTTCCCAAGCA | chlorella_pl_25 | CACCACCTGTGTCCACTCTGGAACT | delta_1_25 | CATACCTTAGGCGCCTGCATCCCCG |
| polaribacters_1 | GCCAGATGGCTGCTCATTGTCCATA | plastid_1_1 | GGTCTCACGACTTGGCATCTCATTG | delta_2_1 | CTCCAGTCTTTCGATAGGATTCCCG |
| polaribacters_2 | TGCCAGATGGCTGCTCATTGTCCAT | plastid_1_2 | TCTCCCTAGGCAGGTTTTTGACCTG | delta_2_2 | GGCCACCCTTGATCCAAAAACCCGA |
| polaribacters_3 | TTGCCAGATGGCTGCTCATTGTCCA | plastid_1_3 | CCACGTGGATTCGATACACGCAATG | delta_2_3 | AGGCCACCCTTGATCCAAAAACCCG |
| polaribacters_4 | CCAGATGGCTGCTCATTGTCCATAC | plastid_1_4 | ATGCACCACCTGTATGTGTCTGCCG | delta_2_4 | AAGGGCACTCCAGTCTTTCGATAGG |
| polaribacters_5 | GTTGCCAGATGGCTGCTCATTGTCC | plastid_1_5 | CACCACCTGTATGTGTCTGCCGAAG | delta_2_5 | GAGGCCACCCTTGATCCAAAAACCC |
| polaribacters_6 | TCCCTCAGCGTCAGTACATACGTAG | plastid_1_6 | AACACCACGTGGATTCGATACACGC | delta_2_6 | GAAGGGCACTCCAGTCTTTCGATAG |
| polaribacters_7 | CCCTCAGCGTCAGTACATACGTAGT | plastid_1_7 | ACCACCTGTATGTGTCTGCCGAAGC | delta_2_7 | ACCCTAGCAAGCTAGAGTGTTCTCG |
| polaribacters_8 | GTCCCTCAGCGTCAGTACATACGTA | plastid_1_8 | CTTCTCCCTAGGCAGGTTTTTGACC | delta_2_8 | CATGTAGAGGCCACCCTTGATCCAA |
| polaribacters_9 | CAGATGGCTGCTCATTGTCCATACC | plastid_1_9 | TGCACCACCTGTATGTGTCTGCCGA | delta_2_9 | AGAGGCCACCCTTGATCCAAAAACC |
| polaribacters_10 | TTCGCATAGTGGCTGCTCATTGTCC | plastid_1_10 | ACACCACGTGGATTCGATACACGCA | delta_2_10 | ACATGTAGAGGCCACCCTTGATCCA |
| polaribacters_11 | CGTCCCTCAGCGTCAGTACATACGT | plastid_1_11 | CCACCTGTATGTGTCTGCCGAAGCA | delta_2_11 | TACATGTAGAGGCCACCCTTGATCC |
| polaribacters_12 | AGACCCCCTACCTATCGTTGCCATG | plastid_1_12 | GCACCACCTGTATGTGTCTGCCGAA | delta_2_12 | CCCCGAAGGGCACTCCAGTCTTTCG |
| polaribacters_13 | CGCTTAGTCACTGAGCTAATGCCCA | plastid_1_13 | CACCACGTGGATTCGATACACGCAA | delta_2_13 | CCCTAGCAAGCTAGAGTGTTCTCGT |
| polaribacters_14 | TGTTGCCAGATGGCTGCTCATTGTC | plastid_1_14 | CTCACGACTTGGCATCTCATTGTCC | delta_2_14 | GCTTACATGTAGAGGCCACCCTTGA |
| polaribacters_15 | GATTCGCTCCTATTCGCATAGTGGC | plastid_1_15 | CAGGTACACGTCAGAAACTTCCTCC | delta_2_15 | GGGCACTCCAGTCTTTCGATAGGAT |
| polaribacters_16 | TCGTCCCTCAGCGTCAGTACATACG | plastid_1_16 | CTCCCTAGGCAGGTTTTTGACCTGT | delta_2_16 | CCGAAGGGCACTCCAGTCTTTCGAT |
| polaribacters_17 | TCGCTTAGTCACTGAGCTAATGCCC | plastid_1_17 | CGGTCTCACGACTTGGCATCTCATT | delta_2_17 | CGAAGGGCACTCCAGTCTTTCGATA |
| polaribacters_18 | TCGCATAGTGGCTGCTCATTGTCCA | plastid_1_18 | GACCAACTACTGATCGTCACCTTGG | delta_2_18 | AGGGCACTCCAGTCTTTCGATAGGA |
| polaribacters_19 | CAGACCCCCTACCTATCGTTGCCAT | plastid_1_19 | GCTTCTCCCTAGGCAGGTTTTTGAC | delta_2_19 | CCCGAAGGGCACTCCAGTCTTTCGA |
| polaribacters_20 | TTCGTCCCTCAGCGTCAGTACATAC | plastid_1_20 | CACCTGTATGTGTCTGCCGAAGCAC | delta_2_20 | CCAGTCTTTCGATAGGATTCCCGGG |
| polaribacters_21 | CTCTCTGTTGCCAGATGGCTGCTCA | plastid_1_21 | CTGTATGTGTCTGCCGAAGCACTTC | delta_2_21 | TCCAGTCTTTCGATAGGATTCCCGG |
| polaribacters_22 | GCAGATTCTATACGCGTTACGCACC | plastid_1_22 | CATGCACCACCTGTATGTGTCTGCC | delta_2_22 | GTCTTTCGATAGGATTCCCGGGATG |
| polaribacters_23 | GGCAGATTCTATACGCGTTACGCAC | plastid_1_23 | AGGTACACGTCAGAAACTTCCTCCC | delta_2_23 | CTTTCGATAGGATTCCCGGGATGTC |
| polaribacters_24 | CACCTCTGACTTAATTGACCGCCTG | plastid_1_24 | TCGGTCTCACGACTTGGCATCTCAT | delta_2_24 | CAGTCTTTCGATAGGATTCCCGGGA |
| polaribacters_25 | CCTCTGACTTAATTGACCGCCTGCG | plastid_1_25 | CCTTCTACTTCGACTCTACTCGAGC | delta_2_25 | GGGCTCCCCGAAGGGCACTCCAGTC |
| desulfovibrionales_1 | CCCGAGCATGCTGATCTCGAATTAC | plastid_2_1 | CAGGTAACGTCAGAACTTCCTCCCT | delta_3_1 | GGCACAGAAAGGGTCAACACTTCCT |
| desulfovibrionales_2 | CACCCGAGCATGCTGATCTCGAATT | plastid_2_2 | AGGTAACGTCAGAACTTCCTCCCTG | delta_3_2 | TCGGCACAGAAAGGGTCAACACTTC |
| desulfovibrionales_3 | TCACCCGAGCATGCTGATCTCGAAT | plastid_2_3 | GGTAACGTCAGAACTTCCTCCCTGA | delta_3_3 | CGGCACAGAAAGGGTCAACACTTCC |
| desulfovibrionales_4 | TTCACCCGAGCATGCTGATCTCGAA | plastid_2_4 | TCAGGTAACGTCAGAACTTCCTCCC | delta_3_4 | CTTCGGCACAGAAAGGGTCAACACT |
| desulfovibrionales_5 | GCACCCTCTAATTTCCTAGAGGTCC | plastid_2_5 | CGCGTTAGCTATAATACCGCATGGG | delta_3_5 | CACTTTACTCTCCCGACGAATCGGA |
| desulfovibrionales_6 | AGGGCACCCTCTAATTTCCTAGAGG | plastid_2_6 | AATACCGCATGGGTCGATACATGCG | delta_3_6 | CCACTTTACTCTCCCGACGAATCGG |
| desulfovibrionales_7 | GGGCACCCTCTAATTTCCTAGAGGT | plastid_2_7 | CTGTATGTACGTTCCCGAAGGTGGT | delta_3_7 | GCTTCGGCACAGAAAGGGTCAACAC |
| desulfovibrionales_8 | CCCTCTAATTTCCTAGAGGTCCCCT | plastid_2_8 | CCTGTATGTACGTTCCCGAAGGTGG | delta_3_8 | CTCTCCCGACGAATCGGAATTTCTC |
| desulfovibrionales_9 | ACCCTCTAATTTCCTAGAGGTCCCC | plastid_2_9 | TCAGCCGCGAGCTCCTCTCTAGGCA | delta_3_9 | CCGACGAATCGGAATTTCTCGTTCG |
| desulfovibrionales_10 | ATTTCCTAGAGGTCCCCTGGATGTC | plastid_2_10 | ATACCGCATGGGTCGATACATGCGA | delta_3_10 | GCCACTTTACTCTCCCGACGAATCG |
| desulfovibrionales_11 | AGGGTACCGTCAAATGCCTACCCTA | plastid_2_11 | ACCTGTATGTACGTTCCCGAAGGTG | delta_3_11 | AGCTTCGGCACAGAAAGGGTCAACA |
| desulfovibrionales_12 | GAGGGTACCGTCAAATGCCTACCCT | plastid_2_12 | GCCGCGAGCTCCTCTCTAGGCAGAA | delta_3_12 | ACTCTCACGAGTTCGCTACCCTTTG |
| desulfovibrionales_13 | GGGTACCGTCAAATGCCTACCCTAT | plastid_2_13 | GCGCCTTCCTCCAAACGGTTAGAAT | delta_3_13 | TCTCCCGACGAATCGGAATTTCTCG |
| desulfovibrionales_14 | TTTCCTAGAGGTCCCCTGGATGTCA | plastid_2_14 | AGCCGCGAGCTCCTCTCTAGGCAGA | delta_3_14 | TAGCTTCGGCACAGAAAGGGTCAAC |
| desulfovibrionales_15 | TTCCTAGAGGTCCCCTGGATGTCAA | plastid_2_15 | CAGCCGCGAGCTCCTCTCTAGGCAG | delta_3_15 | CTCTCACGAGTTCGCTACCCTTTGT |
| desulfovibrionales_16 | TGAGGGTACCGTCAAATGCCTACCC | plastid_2_16 | CACCTGTATGTACGTTCCCGAAGGT | delta_3_16 | GTGCTGGTTACACCCGAAGGCAATC |
| desulfovibrionales_17 | CTCTAATTTCCTAGAGGTCCCCTGG | plastid_2_17 | AATCAGCCGCGAGCTCCTCTCTAGG | delta_3_17 | CGCCACTTTACTCTCCCGACGAATC |
| desulfovibrionales_18 | CACCCTCTAATTTCCTAGAGGTCCC | plastid_2_18 | TAATCAGCCGCGAGCTCCTCTCTAG | delta_3_18 | CTCCCGACGAATCGGAATTTCTCGT |
| desulfovibrionales_19 | GGCACCCTCTAATTTCCTAGAGGTC | plastid_2_19 | ATCAGCCGCGAGCTCCTCTCTAGGC | delta_3_19 | CTTACTCTCACGAGTTCGCTACCCT |
| desulfovibrionales_20 | CCTCTAATTTCCTAGAGGTCCCCTG | plastid_2_20 | GGCGCCTTCCTCCAAACGGTTAGAA | delta_3_20 | TGTGCTGGTTACACCCGAAGGCAAT |
| desulfovibrionales_21 | CAACCGTTATCCCCGTCTTGAAGGT | plastid_2_21 | CCGCGAGCTCCTCTCTAGGCAGAAA | delta_3_21 | CTCACGAGTTCGCTACCCTTTGTAC |
| desulfovibrionales_22 | ATCAAAGGCTGTTCCACCGTTGAGC | plastid_2_22 | GCATGGGTCGATACATGCGACATCT | delta_3_22 | CTGTGCTGGTTACACCCGAAGGCAA |
| desulfovibrionales_23 | TTGCTCGTTAGCTCGCCGGCTTCGG | plastid_2_23 | CCGCATGGGTCGATACATGCGACAT | delta_3_23 | TCGCCACTTTACTCTCCCGACGAAT |
| desulfovibrionales_24 | ATTGCTCGTTAGCTCGCCGGCTTCG | plastid_2_24 | TACCGCATGGGTCGATACATGCGAC | delta_3_24 | CCTGTGCTGGTTACACCCGAAGGCA |
| desulfovibrionales_25 | CCTAGAGGTCCCCTGGATGTCAAGC | plastid_2_25 | ACCGCATGGGTCGATACATGCGACA | delta_3_25 | GCTTACTCTCACGAGTTCGCTACCC |
| aquaficae_1 | AACCAGACGCTCCACCGGTTGTGCG | plastid_3_1 | CACCGTCGTATATCTGACCGACGAT | altero_1_1 | CCCACTTGGGCCAATCTAAAGGCGA |
| aquaficae_2 | ACCAGACGCTCCACCGGTTGTGCGG | plastid_3_2 | TTCACCGTCGTATATCTGACCGACG | altero_1_2 | ATCCCACTTGGGCCAATCTAAAGGC |
| aquaficae_3 | AAACCAGACGCTCCACCGGTTGTGC | plastid_3_3 | TCACCGTCGTATATCTGACCGACGA | altero_1_3 | TCCCACTTGGGCCAATCTAAAGGCG |
| aquaficae_4 | TGCCACTGTAGCGCCTGTGTAGCCC | plastid_3_4 | GTAGCCGAGTTTCAGGCTACAATCC | altero_1_4 | CCACTTGGGCCAATCTAAAGGCGAG |
| aquaficae_5 | TAAACCAGACGCTCCACCGGTTGTG | plastid_3_5 | TAGCCGAGTTTCAGGCTACAATCCG | altero_1_5 | CACTTGGGCCAATCTAAAGGCGAGA |
| aquaficae_6 | GCCACTGTAGCGCCTGTGTAGCCCA | plastid_3_6 | GACCTCATCCTCACCTTCCTCCAAT | altero_1_6 | ACTTGGGCCAATCTAAAGGCGAGAG |
| aquaficae_7 | CCAGACGCTCCACCGGTTGTGCGGG | plastid_3_7 | AGCCGAGTTTCAGGCTACAATCCGA | altero_1_7 | CTTGGGCCAATCTAAAGGCGAGAGC |
| aquaficae_8 | CCACTGTAGCGCCTGTGTAGCCCAG | plastid_3_8 | GCCGAGTTTCAGGCTACAATCCGAA | altero_1_8 | CTGTCAGTAACGTCACAGCTAGCAG |
| aquaficae_9 | GCATAAAGGGCATACTGACCTGACG | plastid_3_9 | CCGAGTTTCAGGCTACAATCCGAAC | altero_1_9 | ACAGAACCGAGGTTCCGAGCTTCTA |
| aquaficae_10 | TTAAACCAGACGCTCCACCGGTTGT | plastid_3_10 | CTCCCGTAGGAGTCTGTTCCGTTCT | altero_1_10 | CAGAACCGAGGTTCCGAGCTTCTAG |
| aquaficae_11 | CATTGCCCACGATTCCCCACTGCTG | plastid_3_11 | CCTCCCGTAGGAGTCTGTTCCGTTC | altero_1_11 | AGAACCGAGGTTCCGAGCTTCTAGT |
| aquaficae_12 | ATTGCCCACGATTCCCCACTGCTGC | plastid_3_12 | TCCCGTAGGAGTCTGTTCCGTTCTA | altero_1_12 | GAAAAACAGAACCGAGGTTCCGAGC |
| aquaficae_13 | CCATTGCCCACGATTCCCCACTGCT | plastid_3_13 | CCCGTAGGAGTCTGTTCCGTTCTAA | altero_1_13 | GAACCGAGGTTCCGAGCTTCTAGTA |
| aquaficae_14 | GCCCATTGCCCACGATTCCCCACTG | plastid_3_14 | TGACCTCATCCTCACCTTCCTCCAA | altero_1_14 | CCGAGGTTCCGAGCTTCTAGTAGAC |
| aquaficae_15 | CCCATTGCCCACGATTCCCCACTGC | plastid_3_15 | CTAAAGCATTCATCCTCCACGCGGT | altero_1_15 | CGAGGTTCCGAGCTTCTAGTAGACA |
| aquaficae_16 | CGCCCATTGCCCACGATTCCCCACT | plastid_3_16 | CCTAAAGCATTCATCCTCCACGCGG | altero_1_16 | AACCGAGGTTCCGAGCTTCTAGTAG |
| aquaficae_17 | TGCCCACGATTCCCCACTGCTGCCC | plastid_3_17 | CCCTAAAGCATTCATCCTCCACGCG | altero_1_17 | ACCGAGGTTCCGAGCTTCTAGTAGA |
| aquaficae_18 | ATTAAACCAGACGCTCCACCGGTTG | plastid_3_18 | ACCCTAAAGCATTCATCCTCCACGC | altero_1_18 | AACAGAACCGAGGTTCCGAGCTTCT |
| aquaficae_19 | TTGCCCACGATTCCCCACTGCTGCC | plastid_3_19 | ACATAAGGGGCATGCTGACTTGACC | altero_1_19 | AAACAGAACCGAGGTTCCGAGCTTC |
| aquaficae_20 | GCCCACGATTCCCCACTGCTGCCCC | plastid_3_20 | GTTCCGTTCTAAATCCCAGTGTGGC | altero_1_20 | CCAACTGTTGTCCCCCACCTCAAGG |
| aquaficae_21 | CAGACGCTCCACCGGTTGTGCGGGC | plastid_3_21 | CATAAGGGGCATGCTGACTTGACCT | altero_1_21 | CCGGACTACGACGCACTTTAAGTGA |
| aquaficae_22 | GGCATAAAGGGCATACTGACCTGAC | plastid_3_22 | GCGGTATTGCTTGGTCAAGCTTTCG | altero_1_22 | TGGGCCAATCTAAAGGCGAGAGCCG |
| aquaficae_23 | GCAGTTCGGAATGCCTTGCCGAAGT | plastid_3_23 | CGGTATTGCTTGGTCAAGCTTTCGC | altero_1_23 | GGGCCAATCTAAAGGCGAGAGCCGA |
| aquaficae_24 | CAGTTCGGAATGCCTTGCCGAAGTT | plastid_3_24 | CACGCGGTATTGCTTGGTCAAGCTT | altero_1_24 | TTGGGCCAATCTAAAGGCGAGAGCC |
| aquaficae_25 | CGCAGTTCGGAATGCCTTGCCGAAG | plastid_3_25 | CATCCTCCACGCGGTATTGCTTGGT | altero_1_25 | GGTTCCGAGCTTCTAGTAGACATCG |
| bacilli_1 | CACTCTGCTCCCGAAGGAGAAGCCC | plastid_4_1 | CTTAAGCGCCGCCCTCCGAATGGTT | altero_2_1 | TCTCACTTGGGCCTCTCTTTGCGCC |
| bacilli_2 | GTCACTCTGCTCCCGAAGGAGAAGC | plastid_4_2 | CCTTAAGCGCCGCCCTCCGAATGGT | altero_2_2 | CCCCTCGCAAAGGCAAGTTCCCAAG |
| bacilli_3 | CTGCTCCCGAAGGAGAAGCCCTATC | plastid_4_3 | TACCTTAAGCGCCGCCCTCCGAATG | altero_2_3 | CCCTCGCAAAGGCAAGTTCCCAAGC |
| bacilli_4 | TCACTCTGCTCCCGAAGGAGAAGCC | plastid_4_4 | ACCTTAAGCGCCGCCCTCCGAATGG | altero_2_4 | TCACTTGGGCCTCTCTTTGCGCCGG |
| bacilli_5 | TCTGCTCCCGAAGGAGAAGCCCTAT | plastid_4_5 | AGCCCTACCTTAAGCGCCGCCCTCC | altero_2_5 | CTTGGGCCTCTCTTTGCGCCGGAGC |
| bacilli_6 | TGCTCCCGAAGGAGAAGCCCTATCT | plastid_4_6 | TTAAGCGCCGCCCTCCGAATGGTTA | altero_2_6 | CGACATTCTTTAAGGGGTCCGCTCC |
| bacilli_7 | CTCTGCTCCCGAAGGAGAAGCCCTA | plastid_4_7 | TAAGCGCCGCCCTCCGAATGGTTAG | altero_2_7 | CACTTGGGCCTCTCTTTGCGCCGGA |
| bacilli_8 | GCTCCCGAAGGAGAAGCCCTATCTC | plastid_4_8 | TAGCCCTACCTTAAGCGCCGCCCTC | altero_2_8 | CTCACTTGGGCCTCTCTTTGCGCCG |
| bacilli_9 | ACTCTGCTCCCGAAGGAGAAGCCCT | plastid_4_9 | CTACCTTAAGCGCCGCCCTCCGAAT | altero_2_9 | ACTTGGGCCTCTCTTTGCGCCGGAG |
| bacilli_10 | CCGAAGCCGCCTTTCAATTTCGAAC | plastid_4_10 | GCCCTACCTTAAGCGCCGCCCTCCG | altero_2_10 | CTACGACATTCTTTAAGGGGTCCGC |
| bacilli_11 | CGTCCGCCGCTAACTTCATAAGAGC | plastid_4_11 | CCCTACCTTAAGCGCCGCCCTCCGA | altero_2_11 | CCGGACTACGACATTCTTTAAGGGG |
| bacilli_12 | GTCCGCCGCTAACTTCATAAGAGCA | plastid_4_12 | CCTACCTTAAGCGCCGCCCTCCGAA | altero_2_12 | ATCTCACTTGGGCCTCTCTTTGCGC |
| bacilli_13 | CCGCCGCTAACTTCATAAGAGCAAG | plastid_4_13 | CTAGCCCTACCTTAAGCGCCGCCCT | altero_2_13 | CCCCCTCGCAAAGGCAAGTTCCCAA |
| bacilli_14 | AGCCGAAGCCGCCTTTCAATTTCGA | plastid_4_14 | ACTAGCCCTACCTTAAGCGCCGCCC | altero_2_14 | ACATTCTTTAAGGGGTCCGCTCCAC |
| bacilli_15 | CTCCCGAAGGAGAAGCCCTATCTCT | plastid_4_15 | AAGCGCCGCCCTCCGAATGGTTAGG | altero_2_15 | TTGGGCCTCTCTTTGCGCCGGAGCC |
| bacilli_16 | CAGCCGAAGCCGCCTTTCAATTTCG | plastid_4_16 | CACTAGCCCTACCTTAAGCGCCGCC | altero_2_16 | TCCCCCTCGCAAAGGCAAGTTCCCA |
| bacilli_17 | CTGTCACTCTGCTCCCGAAGGAGAA | plastid_4_17 | CGCCGCCCTCCGAATGGTTAGGCTA | altero_2_17 | CCTCGCAAAGGCAAGTTCCCAAGCA |
| bacilli_18 | GCCGAAGCCGCCTTTCAATTTCGAA | plastid_4_18 | GCGCCGCCCTCCGAATGGTTAGGCT | altero_2_18 | GGGTCCGCTCCACATCACTGTCTCG |
| bacilli_19 | CCCGTCCGCCGCTAACTTCATAAGA | plastid_4_19 | GCCGCCCTCCGAATGGTTAGGCTAA | altero_2_19 | ACGACATTCTTTAAGGGGTCCGCTC |
| bacilli_20 | CCGTCCGCCGCTAACTTCATAAGAG | plastid_4_20 | AGCGCCGCCCTCCGAATGGTTAGGC | altero_2_20 | CATTCTTTAAGGGGTCCGCTCCACA |
| bacilli_21 | CGCCGCTAACTTCATAAGAGCAAGC | plastid_4_21 | ACGAGATTAGCTAGCCTTCGCAGGT | altero_2_21 | GACATTCTTTAAGGGGTCCGCTCCA |
| bacilli_22 | CCCGAAGGAGAAGCCCTATCTCTAG | plastid_4_22 | CCGCCCTCCGAATGGTTAGGCTAAC | altero_2_22 | AATCTCACTTGGGCCTCTCTTTGCG |
| bacilli_23 | CGAAGGAGAAGCCCTATCTCTAGGG | plastid_4_23 | CGCCCTCCGAATGGTTAGGCTAACG | altero_2_23 | TAAGGGGTCCGCTCCACATCACTGT |
| bacilli_24 | CCGAAGGAGAAGCCCTATCTCTAGG | plastid_4_24 | GCCCTCCGAATGGTTAGGCTAACGA | altero_2_24 | ATCCCCCTCGCAAAGGCAAGTTCCC |
| bacilli_25 | TGTCACTCTGCTCCCGAAGGAGAAG | plastid_4_25 | TCACTAGCCCTACCTTAAGCGCCGC | altero_2_25 | GGTCCGCTCCACATCACTGTCTCGC |
| crenarch_1_1 | AGCCTGTACGTTGAGCGTACAGATT | plastid_5_1 | CTCTACCCCTACCATACTCAAGCCT | colwel_1_1 | TGCGCCACTCACGGATCAAGTCCAC |
| crenarch_1_2 | CCTGTACGTTGAGCGTACAGATTTA | plastid_5_2 | GACGTCGTCCTCCAAATGGTTAGAC | colwel_1_2 | CTGCGCCACTCACGGATCAAGTCCA |
| crenarch_1_3 | GCCTGTACGTTGAGCGTACAGATTT | plastid_5_3 | CCTTAGACGTCGTCCTCCAAATGGT | colwel_1_3 | GCTGCGCCACTCACGGATCAAGTCC |
| crenarch_1_4 | GAGCGTACAGATTTAACCGAAAACT | plastid_5_4 | ACCTTAGACGTCGTCCTCCAAATGG | colwel_1_4 | TAGCTGCGCCACTCACGGATCAAGT |
| crenarch_1_5 | TGAGCGTACAGATTTAACCGAAAAC | plastid_5_5 | CCTCTACCCCTACCATACTCAAGCC | colwel_1_5 | GTTAGCTGCGCCACTCACGGATCAA |
| crenarch_1_6 | CAGCCTGTACGTTGAGCGTACAGAT | plastid_5_6 | GCTAGTTCTCGCGAATTTGCGACTC | colwel_1_6 | CGTTAGCTGCGCCACTCACGGATCA |
| crenarch_1_7 | CCTTGTCACGAACCTCAAGTTCGAT | plastid_5_7 | CCTCTCGGCATATGGGGATTTAGCT | colwel_1_7 | GTGCGTTAGCTGCGCCACTCACGGA |
| crenarch_1_8 | CTTGTCACGAACCTCAAGTTCGATA | plastid_5_8 | GACTAACGGTGTTGGGTATGACCAG | colwel_1_8 | TGCGTTAGCTGCGCCACTCACGGAT |
| crenarch_1_9 | TTGTCACGAACCTCAAGTTCGATAA | plastid_5_9 | ACTAACGGTGTTGGGTATGACCAGC | colwel_1_9 | TTAGCTGCGCCACTCACGGATCAAG |
| crenarch_1_10 | CTGTACGTTGAGCGTACAGATTTAA | plastid_5_10 | CCAACAGTTATTCCCCTCCTAAGGG | colwel_1_10 | GCGTTAGCTGCGCCACTCACGGATC |
| crenarch_1_11 | GTCACGAACCTCAAGTTCGATAACG | plastid_5_11 | CTCTCGGCATATGGGGATTTAGCTG | colwel_1_11 | AGCTGCGCCACTCACGGATCAAGTC |
| crenarch_1_12 | TTCCCTTGTCACGAACCTCAAGTTC | plastid_5_12 | GCGCGAGCTCATCCTTAGGCAGTGT | colwel_1_12 | GCGGTATTGCTGCCCTCTGTACCTG |
| crenarch_1_13 | TCACGAACCTCAAGTTCGATAACGC | plastid_5_13 | CGCGAGCTCATCCTTAGGCAGTGTA | colwel_1_13 | CGCGGTATTGCTGCCCTCTGTACCT |
| crenarch_1_14 | TGTCACGAACCTCAAGTTCGATAAC | plastid_5_14 | GCGAGCTCATCCTTAGGCAGTGTAA | colwel_1_14 | GGATCAAGTCCACGAACGGCTAGTT |
| crenarch_1_15 | CTGCAGCACTGCATTGGCCACAAGC | plastid_5_15 | CACCTCTCGGCATATGGGGATTTAG | colwel_1_15 | CGGATCAAGTCCACGAACGGCTAGT |
| crenarch_1_16 | GCAGCCTGTACGTTGAGCGTACAGA | plastid_5_16 | ACCTCTCGGCATATGGGGATTTAGC | colwel_1_16 | GCGCCACTCACGGATCAAGTCCACG |
| crenarch_1_17 | CACGAACCTCAAGTTCGATAACGCC | plastid_5_17 | GCAGCCTACAATCCGAACTTGGACA | colwel_1_17 | ACGGATCAAGTCCACGAACGGCTAG |
| crenarch_1_18 | TGTACGTTGAGCGTACAGATTTAAC | plastid_5_18 | GGCGCGAGCTCATCCTTAGGCAGTG | colwel_1_18 | CACGGATCAAGTCCACGAACGGCTA |
| crenarch_1_19 | CGTTGAGCGTACAGATTTAACCGAA | plastid_5_19 | CGGCAGTCTCTCTAGAGATCCCAAT | colwel_1_19 | CGCCACTCACGGATCAAGTCCACGA |
| crenarch_1_20 | GTACGTTGAGCGTACAGATTTAACC | plastid_5_20 | ATCACCGGCAGTCTCTCTAGAGATC | colwel_1_20 | GCCACTCACGGATCAAGTCCACGAA |
| crenarch_1_21 | CCTGCAGCACTGCATTGGCCACAAG | plastid_5_21 | CACCGGCAGTCTCTCTAGAGATCCC | colwel_1_21 | TCACGGATCAAGTCCACGAACGGCT |
| crenarch_1_22 | GGCAGCCTGTACGTTGAGCGTACAG | plastid_5_22 | ACCGGCAGTCTCTCTAGAGATCCCA | colwel_1_22 | GATCAAGTCCACGAACGGCTAGTTG |
| crenarch_1_23 | TACGTTGAGCGTACAGATTTAACCG | plastid_5_23 | CCGGCAGTCTCTCTAGAGATCCCAA | colwel_1_23 | ACTCACGGATCAAGTCCACGAACGG |
| crenarch_1_24 | ACGTTGAGCGTACAGATTTAACCGA | plastid_5_24 | TTCGCCTCTCAGTGTCAGTAATGGC | colwel_1_24 | CACTCACGGATCAAGTCCACGAACG |
| crenarch_1_25 | CCACTCCCTAGCTCTGCAGTATTCC | plastid_5_25 | TCGCCTCTCAGTGTCAGTAATGGCC | colwel_1_25 | CTCACGGATCAAGTCCACGAACGGC |
| acido_1_1 | TGCAGCACCTCTTCTGGAGTCCCCG | margrpA_1_1 | GCTCCGGTACCGAAGGGGTCGAATC | altero_3_1 | CAACTGTTGTCCCCCACGTTTTGGC |
| acido_1_2 | GCCGGCAGTCCCCCCAAAGTCCCCG | margrpA_1_2 | AGCTCCGGTACCGAAGGGGTCGAAT | altero_3_2 | AACTGTTGTCCCCCACGTTTTGGCA |
| acido_1_3 | CCATGCAGCACCTCTTCTGGAGTCC | margrpA_1_3 | CACCCGATTCGGGTACTACTGACTT | altero_3_3 | CCCCACGTTTTGGCATATTCCCAAG |
| acido_1_4 | CATGCAGCACCTCTTCTGGAGTCCC | margrpA_1_4 | ACCCGATTCGGGTACTACTGACTTC | altero_3_4 | CCCACGTTTTGGCATATTCCCAAGC |
| acido_1_5 | GCGCCGGCAGTCCCCCCAAAGTCCC | margrpA_1_5 | CTCCGGTACCGAAGGGGTCGAATCC | altero_3_5 | TCCCCCACGTTTTGGCATATTCCCA |
| acido_1_6 | ATGCAGCACCTCTTCTGGAGTCCCC | margrpA_1_6 | CCACCCGATTCGGGTACTACTGACT | altero_3_6 | CCCCCACGTTTTGGCATATTCCCAA |
| acido_1_7 | CGCCGGCAGTCCCCCCAAAGTCCCC | margrpA_1_7 | GCCACCCGATTCGGGTACTACTGAC | altero_3_7 | CCAACTGTTGTCCCCCACGTTTTGG |
| acido_1_8 | GCAGCACCTCTTCTGGAGTCCCCGA | margrpA_1_8 | GGCCACCCGATTCGGGTACTACTGA | altero_3_8 | GTCCCCCACGTTTTGGCATATTCCC |
| acido_1_9 | CAGCACCTCTTCTGGAGTCCCCGAA | margrpA_1_9 | TAGCTCCGGTACCGAAGGGGTCGAA | altero_3_9 | ACTGTTGTCCCCCACGTTTTGGCAT |
| acido_1_10 | AGCACCTCTTCTGGAGTCCCCGAAG | margrpA_1_10 | TCCGGTACCGAAGGGGTCGAATCCC | altero_3_10 | TCCAACTGTTGTCCCCCACGTTTTG |
| acido_1_11 | CCGGCAGTCCCCCCAAAGTCCCCGG | margrpA_1_11 | GAAGGGGTCGAATCCCCCGACACCA | altero_3_11 | TGTCCCCCACGTTTTGGCATATTCC |
| acido_1_12 | GCAGTCCCCCCAAAGTCCCCGGCAT | margrpA_1_12 | AAGGGGTCGAATCCCCCGACACCAA | altero_3_12 | GCATACCATCGCTGGTTAGCAACCC |
| acido_1_13 | GCACCTCTTCTGGAGTCCCCGAAGG | margrpA_1_13 | CTTCCCTTACGACAGACCTTTACGC | altero_3_13 | CGCATACCATCGCTGGTTAGCAACC |
| acido_1_14 | GCCATGCAGCACCTCTTCTGGAGTC | margrpA_1_14 | CCCGATTCGGGTACTACTGACTTCC | altero_3_14 | TCGCATACCATCGCTGGTTAGCAAC |
| acido_1_15 | ACCTCTTCTGGAGTCCCCGAAGGGA | margrpA_1_15 | ACAACTGTATCCCGAAGGATCCGCT | altero_3_15 | CTGTTGTCCCCCACGTTTTGGCATA |
| acido_1_16 | CACCTCTTCTGGAGTCCCCGAAGGG | margrpA_1_16 | CAACTGTATCCCGAAGGATCCGCTG | altero_3_16 | CTTGGGCTAATCAAAACGCGCAAGG |
| acido_1_17 | CGGCAGTCCCCCCAAAGTCCCCGGC | margrpA_1_17 | AACTGTATCCCGAAGGATCCGCTGC | altero_3_17 | TCCCACTTGGGCTAATCAAAACGCG |
| acido_1_18 | CCCCGAAGGGGCCTTACCGCTCAAC | margrpA_1_18 | AACAACTGTATCCCGAAGGATCCGC | altero_3_18 | TTGGGCTAATCAAAACGCGCAAGGC |
| acido_1_19 | CCTCTTCTGGAGTCCCCGAAGGGAA | margrpA_1_19 | GTTAGCTCCGGTACCGAAGGGGTCG | altero_3_19 | CCCACTTGGGCTAATCAAAACGCGC |
| acido_1_20 | GGCAGTCCCCCCAAAGTCCCCGGCA | margrpA_1_20 | TTAGCTCCGGTACCGAAGGGGTCGA | altero_3_20 | TCACCGGCAGTCTCCCTATAGTTCC |
| acido_1_21 | AGCCATGCAGCACCTCTTCTGGAGT | margrpA_1_21 | GCGTTAGCTCCGGTACCGAAGGGGT | altero_3_21 | TGGGCTAATCAAAACGCGCAAGGCC |
| acido_1_22 | CAGCCATGCAGCACCTCTTCTGGAG | margrpA_1_22 | CGTTAGCTCCGGTACCGAAGGGGTC | altero_3_22 | CCACTTGGGCTAATCAAAACGCGCA |
| acido_1_23 | CCCCCGAAGGGGCCTTACCGCTCAA | margrpA_1_23 | TGCGTTAGCTCCGGTACCGAAGGGG | altero_3_23 | ATAGTTCCCGACATAACTCGCTGGC |
| acido_1_24 | ACAGCCATGCAGCACCTCTTCTGGA | margrpA_1_24 | TCCCTTACGACAGACCTTTACGCTC | altero_3_24 | CCATCGCTGGTTAGCAACCCTTTGT |
| acido_1_25 | CCGAAGGGGCCTTACCGCTCAACTT | margrpA_1_25 | ACTGTATCCCGAAGGATCCGCTGCA | altero_3_25 | GGGCTAATCAAAACGCGCAAGGCCC |
| acido_2_1 | GTCAACTCCCTCCACACCAAGTGTT | margrpA_2_1 | GCTGCCTTCGCATTTGACTTTCCTC | gamma_1_1 | CTAAAAGGTCAAGCCTCCCAACGGC |
| acido_2_2 | GGTCAACTCCCTCCACACCAAGTGT | margrpA_2_2 | GGCTGCCTTCGCATTTGACTTTCCT | gamma_1_2 | ACTAAAAGGTCAAGCCTCCCAACGG |
| acido_2_3 | GGGTCAACTCCCTCCACACCAAGTG | margrpA_2_3 | AGGCTGCCTTCGCATTTGACTTTCC | gamma_1_3 | GAAGAGGCCCTCTTTCCCTCTTAAG |
| acido_2_4 | TCAACTCCCTCCACACCAAGTGTTC | margrpA_2_4 | ACAACTGTGCTCCGAAGAGCCCGCT | gamma_1_4 | CACTAAAAGGTCAAGCCTCCCAACG |
| acido_2_5 | GGGGTCAACTCCCTCCACACCAAGT | margrpA_2_5 | TAACAACTGTGCTCCGAAGAGCCCG | gamma_1_5 | GCATGTATTAGGCCTGCCGCCAACG |
| acido_2_6 | AGGGGTCAACTCCCTCCACACCAAG | margrpA_2_6 | AACAACTGTGCTCCGAAGAGCCCGC | gamma_1_6 | GGCTCCTCCAATAGTGAGAGCTTTC |
| acido_2_7 | CAACTCCCTCCACACCAAGTGTTCA | margrpA_2_7 | GATACCATCTTCGGGTACTGCAGAC | gamma_1_7 | AAGAGGCCCTCTTTCCCTCTTAAGG |
| acido_2_8 | AAGGGGTCAACTCCCTCCACACCAA | margrpA_2_8 | TTAACAACTGTGCTCCGAAGAGCCC | gamma_1_8 | CAAGAAGAGGCCCTCTTTCCCTCTT |
| acido_2_9 | GAAGGGGTCAACTCCCTCCACACCA | margrpA_2_9 | CAACTGTGCTCCGAAGAGCCCGCTG | gamma_1_9 | TCAAGAAGAGGCCCTCTTTCCCTCT |
| acido_2_10 | AACTCCCTCCACACCAAGTGTTCAT | margrpA_2_10 | CAGAAGGCTGCCTTCGCATTTGACT | gamma_1_10 | TAGCTGCGCCACTAAAAGGTCAAGC |
| acido_2_11 | ACTCCCTCCACACCAAGTGTTCATC | margrpA_2_11 | ACCATCTTCGGGTACTGCAGACTTC | gamma_1_11 | CAGGCTCCTCCAATAGTGAGAGCTT |
| acido_2_12 | CTCCCTCCACACCAAGTGTTCATCG | margrpA_2_12 | TTGCGGTTAGGATACCATCTTCGGG | gamma_1_12 | CTCAGCGTCAGTATCAATCCAGGGG |
| acido_2_13 | CAGTCCCCGTAGAGTTCCCGCCATG | margrpA_2_13 | CTTGCGGTTAGGATACCATCTTCGG | gamma_1_13 | AAAGGTCAAGCCTCCCAACGGCTAG |
| acido_2_14 | TCCCCGTAGAGTTCCCGCCATGACG | margrpA_2_14 | CCTTGCGGTTAGGATACCATCTTCG | gamma_1_14 | GCGTTAGCTGCGCCACTAAAAGGTC |
| acido_2_15 | GTCCCCGTAGAGTTCCCGCCATGAC | margrpA_2_15 | CCATCTTCGGGTACTGCAGACTTCC | gamma_1_15 | GAGGCCCTCTTTCCCTCTTAAGGCG |
| acido_2_16 | AGTCCCCGTAGAGTTCCCGCCATGA | margrpA_2_16 | GGATACCATCTTCGGGTACTGCAGA | gamma_1_16 | AGAGGCCCTCTTTCCCTCTTAAGGC |
| acido_2_17 | GCAGTCCCCGTAGAGTTCCCGCCAT | margrpA_2_17 | ACCTGCCTTACCTTAAACAGCTCCC | gamma_1_17 | CCCCCTCTATCGTACTCTAGCCTAT |
| acido_2_18 | GGCAGTCCCCGTAGAGTTCCCGCCA | margrpA_2_18 | CCTGCCTTACCTTAAACAGCTCCCT | gamma_1_18 | CCCCTCTATCGTACTCTAGCCTATC |
| acido_2_19 | CCGGCACGGAAGGGGTCAACTCCCT | margrpA_2_19 | CCAGAAGGCTGCCTTCGCATTTGAC | gamma_1_19 | TTCAAGAAGAGGCCCTCTTTCCCTC |
| acido_2_20 | ACGCGCTGGCAACTACGGGTAAGGG | margrpA_2_20 | TGCGGTTAGGATACCATCTTCGGGT | gamma_1_20 | AGGCCCTCTTTCCCTCTTAAGGCGT |
| acido_2_21 | GACGCGCTGGCAACTACGGGTAAGG | margrpA_2_21 | CGAAGAGCCCGCTGCATTATTTGGT | gamma_1_21 | GCCCTCTTTCCCTCTTAAGGCGTAT |
| acido_2_22 | TGACGCGCTGGCAACTACGGGTAAG | margrpA_2_22 | CCACCATGAATTCTGCGTTCCTCTC | gamma_1_22 | CCCTCTTTCCCTCTTAAGGCGTATG |
| acido_2_23 | AGCTCCGGCACGGAAGGGGTCAACT | margrpA_2_23 | CCTCCTTGCGGTTAGGATACCATCT | gamma_1_23 | CTCTTTCCCTCTTAAGGCGTATGCG |
| acido_2_24 | GCTCCGGCACGGAAGGGGTCAACTC | margrpA_2_24 | CATCTTCGGGTACTGCAGACTTCCA | gamma_1_24 | CCTCTTTCCCTCTTAAGGCGTATGC |
| acido_2_25 | CTCCGGCACGGAAGGGGTCAACTCC | margrpA_2_25 | CGGTTAGGATACCATCTTCGGGTAC | gamma_1_25 | GGCCCTCTTTCCCTCTTAAGGCGTA |
| acido_3_1 | CTCACGGCATTCGTCCCACTCGACA | OP10_1_1 | CCGCTTGCACGGGCAGTTCCGTAAG | gamma_2_1 | TACCTGCTAGCAACCAGGGATAGGG |
| acido_3_2 | CGAGGTCCCCACGGTGTCATGCGGT | OP10_1_2 | CCCGCTTGCACGGGCAGTTCCGTAA | gamma_2_2 | CAGCATTACCTGCTAGCAACCAGGG |
| acido_3_3 | TCACCCTCACGGCATTCGTCCCACT | OP10_1_3 | CGCTTGCACGGGCAGTTCCGTAAGA | gamma_2_3 | TTACCTGCTAGCAACCAGGGATAGG |
| acido_3_4 | AGGTCCCCACGGTGTCATGCGGTAT | OP10_1_4 | TCCCGCTTGCACGGGCAGTTCCGTA | gamma_2_4 | ACCTGCTAGCAACCAGGGATAGGGG |
| acido_3_5 | GGACCGAGGTCCCCACGGTGTCATG | OP10_1_5 | GGGTGCAGACAATTCAGGTGACTTG | gamma_2_5 | TCAGCATTACCTGCTAGCAACCAGG |
| acido_3_6 | CCGAGGTCCCCACGGTGTCATGCGG | OP10_1_6 | CTCCCGCTTGCACGGGCAGTTCCGT | gamma_2_6 | TCTCCCTGGAGTTCTCAGCATTACC |
| acido_3_7 | ACCCTCACGGCATTCGTCCCACTCG | OP10_1_7 | CCTCCCGCTTGCACGGGCAGTTCCG | gamma_2_7 | GTCTCCCTGGAGTTCTCAGCATTAC |
| acido_3_8 | ACCGAGGTCCCCACGGTGTCATGCG | OP10_1_8 | GCTTGCACGGGCAGTTCCGTAAGAG | gamma_2_8 | CAGTCTCCCTGGAGTTCTCAGCATT |
| acido_3_9 | CACCCTCACGGCATTCGTCCCACTC | OP10_1_9 | CGGGTGCAGACAATTCAGGTGACTT | gamma_2_9 | TCCCTGGAGTTCTCAGCATTACCTG |
| acido_3_10 | GACCGAGGTCCCCACGGTGTCATGC | OP10_1_10 | CCGTAAGAGTTCCCGACTTTACGCT | gamma_2_10 | CTCCCTGGAGTTCTCAGCATTACCT |
| acido_3_11 | CCTCACGGCATTCGTCCCACTCGAC | OP10_1_11 | GCAGACAATTCAGGTGACTTGACGG | gamma_2_11 | GCAGTCTCCCTGGAGTTCTCAGCAT |
| acido_3_12 | TTCACCCTCACGGCATTCGTCCCAC | OP10_1_12 | TCGGGTGCAGACAATTCAGGTGACT | gamma_2_12 | GGCAGTCTCCCTGGAGTTCTCAGCA |
| acido_3_13 | GAGGTCCCCACGGTGTCATGCGGTA | OP10_1_13 | CGTAAGAGTTCCCGACTTTACGCTG | gamma_2_13 | CCTGCTAGCAACCAGGGATAGGGGT |
| acido_3_14 | CCCTCACGGCATTCGTCCCACTCGA | OP10_1_14 | TTGCACGGGCAGTTCCGTAAGAGTT | gamma_2_14 | TGCTAGCAACCAGGGATAGGGGTTG |
| acido_3_15 | GGTCCCCACGGTGTCATGCGGTATT | OP10_1_15 | TCCGTAAGAGTTCCCGACTTTACGC | gamma_2_15 | CTGCTAGCAACCAGGGATAGGGGTT |
| acido_3_16 | GTCCCCACGGTGTCATGCGGTATTA | OP10_1_16 | GGCAGTTCCGTAAGAGTTCCCGACT | gamma_2_16 | TAGCAACCAGGGATAGGGGTTGCGC |
| acido_3_17 | GATTGTTCACCCTCACGGCATTCGT | OP10_1_17 | CTTGCACGGGCAGTTCCGTAAGAGT | gamma_2_17 | AGCAACCAGGGATAGGGGTTGCGCT |
| acido_3_18 | AGGACCGAGGTCCCCACGGTGTCAT | OP10_1_18 | CGGGCAGTTCCGTAAGAGTTCCCGA | gamma_2_18 | CTCAGCATTACCTGCTAGCAACCAG |
| acido_3_19 | ATTGTTCACCCTCACGGCATTCGTC | OP10_1_19 | TGCACGGGCAGTTCCGTAAGAGTTC | gamma_2_19 | CTAGCAACCAGGGATAGGGGTTGCG |
| acido_3_20 | TTGTTCACCCTCACGGCATTCGTCC | OP10_1_20 | ACGGGCAGTTCCGTAAGAGTTCCCG | gamma_2_20 | GCTAGCAACCAGGGATAGGGGTTGC |
| acido_3_21 | TGTTCACCCTCACGGCATTCGTCCC | OP10_1_21 | GCACGGGCAGTTCCGTAAGAGTTCC | gamma_2_21 | GCATTACCTGCTAGCAACCAGGGAT |
| acido_3_22 | GGATTGTTCACCCTCACGGCATTCG | OP10_1_22 | CACGGGCAGTTCCGTAAGAGTTCCC | gamma_2_22 | AGCATTACCTGCTAGCAACCAGGGA |
| acido_3_23 | CACGGCATTCGTCCCACTCGACAGG | OP10_1_23 | GCAGTTCCGTAAGAGTTCCCGACTT | gamma_2_23 | TCGCGAGTTGGCAGCCCTCTGTACG |
| acido_3_24 | TCACGGCATTCGTCCCACTCGACAG | OP10_1_24 | GGGCAGTTCCGTAAGAGTTCCCGAC | gamma_2_24 | CTCGCGAGTTGGCAGCCCTCTGTAC |
| acido_3_25 | GCTTTGATCGCAAGGACCGAGGTCC | OP10_1_25 | CCCCCTTACTCCCCACACCTTAGAC | gamma_2_25 | CGCGAGTTGGCAGCCCTCTGTACGC |
| actino_1_1 | AAACCTAGATCCGTCATCCCACACG | OP3_1_1 | ATCCAAGGGTGATAGGTCCTTACGG | gamma_3_1 | TGCGACACCGAAGGGCAACCCCCCC |
| actino_1_2 | CAAACCTAGATCCGTCATCCCACAC | OP3_1_2 | TCCAAGGGTGATAGGTCCTTACGGA | gamma_3_2 | CTGCGACACCGAAGGGCAACCCCCC |
| actino_1_3 | CACCACCTGTATAGGGCGCTAATGC | OP3_1_3 | CCAAGGGTGATAGGTCCTTACGGAT | gamma_3_3 | GACTAGTTCCGAGTATGTCAAGGGC |
| actino_1_4 | ACCACCTGTATAGGGCGCTAATGCA | OP3_1_4 | TGTTCTCCCCTGCTGACAGGAGTTT | gamma_3_4 | GCTGCGACACCGAAGGGCAACCCCC |
| actino_1_5 | CCACCTGTATAGGGCGCTAATGCAC | OP3_1_5 | TTGTTCTCCCCTGCTGACAGGAGTT | gamma_3_5 | AACGCGCTAGCTGCGACACCGAAGG |
| actino_1_6 | CACCTGTATAGGGCGCTAATGCACA | OP3_1_6 | CTTGTTCTCCCCTGCTGACAGGAGT | gamma_3_6 | TAACGCGCTAGCTGCGACACCGAAG |
| actino_1_7 | GCACCACCTGTATAGGGCGCTAATG | OP3_1_7 | GTTCTCCCCTGCTGACAGGAGTTTA | gamma_3_7 | TTACTTAACCGCCAACGCGCGCTTT |
| actino_1_8 | AACCTAGATCCGTCATCCCACACGC | OP3_1_8 | CATCCAAGGGTGATAGGTCCTTACG | gamma_3_8 | ACGCGCTAGCTGCGACACCGAAGGG |
| actino_1_9 | TGCACCACCTGTATAGGGCGCTAAT | OP3_1_9 | TCGACAGGTTATCCCGAACCCTAGG | gamma_3_9 | TTAACGCGCTAGCTGCGACACCGAA |
| actino_1_10 | AGCCCTGAACTTTCACGACCGACTT | OP3_1_10 | TTCGACAGGTTATCCCGAACCCTAG | gamma_3_10 | CGCGCTAGCTGCGACACCGAAGGGC |
| actino_1_11 | GCCCTGAACTTTCACGACCGACTTG | OP3_1_11 | TTCTCCCCTGCTGACAGGAGTTTAC | gamma_3_11 | TACTTAACCGCCAACGCGCGCTTTA |
| actino_1_12 | GAGCCCTGAACTTTCACGACCGACT | OP3_1_12 | CCATCCAAGGGTGATAGGTCCTTAC | gamma_3_12 | AGCTGCGACACCGAAGGGCAACCCC |
| actino_1_13 | AGCGTCGATAGCGGCCCAGTGAGCT | OP3_1_13 | TGATAGGTCCTTACGGATCCCCATC | gamma_3_13 | CTTACTTAACCGCCAACGCGCGCTT |
| actino_1_14 | GCGTCGATAGCGGCCCAGTGAGCTG | OP3_1_14 | TCTCCCCTGCTGACAGGAGTTTACA | gamma_3_14 | ATCCGACTTACTTAACCGCCAACGC |
| actino_1_15 | CGTCGATAGCGGCCCAGTGAGCTGC | OP3_1_15 | CGGATCCCCATCTTTCCCTCATGTT | gamma_3_15 | CGACTTACTTAACCGCCAACGCGCG |
| actino_1_16 | CAGCGTCGATAGCGGCCCAGTGAGC | OP3_1_16 | TCCTTGCCGGTTAGGCAACCTACTT | gamma_3_16 | TCCGACTTACTTAACCGCCAACGCG |
| actino_1_17 | CCCTGAACTTTCACGACCGACTTGT | OP3_1_17 | AGTGCGCACCGACCGAAGTCGGTGT | gamma_3_17 | CTTAACGCGCTAGCTGCGACACCGA |
| actino_1_18 | TGAGCCCTGAACTTTCACGACCGAC | OP3_1_18 | CCAGTAATGCGCCTTCGCGACTGGT | gamma_3_18 | ACTTACTTAACCGCCAACGCGCGCT |
| actino_1_19 | ACCTAGATCCGTCATCCCACACGCG | OP3_1_19 | AGAGTGCGCACCGACCGAAGTCGGT | gamma_3_19 | GCGCTAGCTGCGACACCGAAGGGCA |
| actino_1_20 | CTCGGGCTATCCCAGTAACTAAGGT | OP3_1_20 | TCGAAAAGCACAGGACGTATCCGGT | gamma_3_20 | CCGACTTACTTAACCGCCAACGCGC |
| actino_1_21 | CCTCGGGCTATCCCAGTAACTAAGG | OP3_1_21 | CTGTGCTTCGAAAAGCACAGGACGT | gamma_3_21 | ACTTAACCGCCAACGCGCGCTTTAC |
| actino_1_22 | TCGATAGCGGCCCAGTGAGCTGCCT | OP3_1_22 | CCTTAGAGTGCGCACCGACCGAAGT | gamma_3_22 | CATCCGACTTACTTAACCGCCAACG |
| actino_1_23 | GTCGATAGCGGCCCAGTGAGCTGCC | OP3_1_23 | GCCCTCCTTGCCGGTTAGGCAACCT | gamma_3_23 | TCTTCACACACGCGGCATTGCTAGA |
| actino_1_24 | CGATAGCGGCCCAGTGAGCTGCCTT | OP3_1_24 | CTCCTTGCCGGTTAGGCAACCTACT | gamma_3_24 | AGAACTTAACGCGCTAGCTGCGACA |
| actino_1_25 | TCCTCGGGCTATCCCAGTAACTAAG | OP3_1_25 | CAGTAATGCGCCTTCGCGACTGGTG | gamma_3_25 | ACTTAACGCGCTAGCTGCGACACCG |
| actino_2_1 | CCGGTTTCCCCAAGTGCAAGCACTT | OP9_1_1 | GGGCAAGATAATGTCAAGTCCCGGT | gamma_4_1 | ACACCGAAAGGCAAACCCTCCCGAC |
| actino_2_2 | CAAGCACTTGGTTCGTCCCTCGACT | OP9_1_2 | GCTGGCACATAATTAGCCGGAGCTT | gamma_4_2 | GACACCGAAAGGCAAACCCTCCCGA |
| actino_2_3 | GCCGGTTTCCCCAAGTGCAAGCACT | OP9_1_3 | TGCTGGCACATAATTAGCCGGAGCT | gamma_4_3 | CACCGAAAGGCAAACCCTCCCGACA |
| actino_2_4 | GCTTCGACACGGAAATCGTGAACTG | OP9_1_4 | CCCACTTACAGGGTAGATTACCCAC | gamma_4_4 | ACCGAAAGGCAAACCCTCCCGACAT |
| actino_2_5 | TTCGCCGGTTTCCCCAAGTGCAAGC | OP9_1_5 | CCCCACTTACAGGGTAGATTACCCA | gamma_4_5 | CGACACCGAAAGGCAAACCCTCCCG |
| actino_2_6 | CGACACGGAAATCGTGAACTGATCC | OP9_1_6 | CCCCCACTTACAGGGTAGATTACCC | gamma_4_6 | CCGAAAGGCAAACCCTCCCGACATC |
| actino_2_7 | GACACGGAAATCGTGAACTGATCCC | OP9_1_7 | CTGCTAACCTCATCATCCCGAAGGA | gamma_4_7 | GCGACACCGAAAGGCAAACCCTCCC |
| actino_2_8 | ACACGGAAATCGTGAACTGATCCCC | OP9_1_8 | TCTGCTAACCTCATCATCCCGAAGG | gamma_4_8 | CGAAAGGCAAACCCTCCCGACATCT |
| actino_2_9 | CGCCGGTTTCCCCAAGTGCAAGCAC | OP9_1_9 | CTGCTGGCACATAATTAGCCGGAGC | gamma_4_9 | GCTGCGACACCGAAAGGCAAACCCT |
| actino_2_10 | ACGGAAATCGTGAACTGATCCCCAC | OP9_1_10 | CCACTTACAGGGTAGATTACCCACG | gamma_4_10 | AGCTGCGACACCGAAAGGCAAACCC |
| actino_2_11 | TCGCCGGTTTCCCCAAGTGCAAGCA | OP9_1_11 | GACGGGCAAGATAATGTCAAGTCCC | gamma_4_11 | TTGGCTAGCCATTGCTGGTTTGCAG |
| actino_2_12 | CACGGAAATCGTGAACTGATCCCCA | OP9_1_12 | TCCCCCACTTACAGGGTAGATTACC | gamma_4_12 | TGGCTAGCCATTGCTGGTTTGCAGC |
| actino_2_13 | CGGTTTCCCCAAGTGCAAGCACTTG | OP9_1_13 | GCAGTCTGCCTAGAGTGCACTTGTA | gamma_4_13 | GGATTGGCTAGCCATTGCTGGTTTG |
| actino_2_14 | AAGTGCAAGCACTTGGTTCGTCCCT | OP9_1_14 | GCTGCTGGCACATAATTAGCCGGAG | gamma_4_14 | GATTGGCTAGCCATTGCTGGTTTGC |
| actino_2_15 | GTTCGCCGGTTTCCCCAAGTGCAAG | OP9_1_15 | GGGTACCGTCAGGCTTAAGGGTTTA | gamma_4_15 | GGGATTGGCTAGCCATTGCTGGTTT |
| actino_2_16 | CGGAAATCGTGAACTGATCCCCACA | OP9_1_16 | CACTTACAGGGTAGATTACCCACGC | gamma_4_16 | GGCTAGCCATTGCTGGTTTGCAGCC |
| actino_2_17 | GCAAGCACTTGGTTCGTCCCTCGAC | OP9_1_17 | GGCAGTCTGCCTAGAGTGCACTTGT | gamma_4_17 | GAAAGGCAAACCCTCCCGACATCTA |
| actino_2_18 | CGTTCGCCGGTTTCCCCAAGTGCAA | OP9_1_18 | GGTTATCCCCCACTTACAGGGTAGA | gamma_4_18 | CTGCGACACCGAAAGGCAAACCCTC |
| actino_2_19 | AAGCACTTGGTTCGTCCCTCGACTT | OP9_1_19 | GAGGGTTATCCCCCACTTACAGGGT | gamma_4_19 | TGCGACACCGAAAGGCAAACCCTCC |
| actino_2_20 | GGTTTCCCCAAGTGCAAGCACTTGG | OP9_1_20 | GGGTTATCCCCCACTTACAGGGTAG | gamma_4_20 | AGGGATTGGCTAGCCATTGCTGGTT |
| actino_2_21 | AGTGCAAGCACTTGGTTCGTCCCTC | OP9_1_21 | GTCAGAGATAGACCAGAAAGCCGCC | gamma_4_21 | AAGGGATTGGCTAGCCATTGCTGGT |
| actino_2_22 | CAAGTGCAAGCACTTGGTTCGTCCC | OP9_1_22 | GGGGTACCGTCAGGCTTAAGGGTTT | gamma_4_22 | TAAGGGATTGGCTAGCCATTGCTGG |
| actino_2_23 | CCGTTCGCCGGTTTCCCCAAGTGCA | OP9_1_23 | AGGGTTATCCCCCACTTACAGGGTA | gamma_4_23 | TAGCTGCGACACCGAAAGGCAAACC |
| actino_2_24 | CCGTAGTTATCCCGGTGTACAGGGC | OP9_1_24 | CGGCAGTCTGCCTAGAGTGCACTTG | gamma_4_24 | TTAGCTGCGACACCGAAAGGCAAAC |
| actino_2_25 | CCTCAAGCCTTGCAGTATCGACTGC | OP9_1_25 | CTCCGCATTATCTGCGGCAGTCTGC | gamma_4_25 | GTTAGCTGCGACACCGAAAGGCAAA |
| bacter_1_1 | GTTTCCGCGACTGTCATTCCACGTT | plancto_1_1 | TGCAACACCTGTGCAGGTCACACCC | gamma_5_1 | CCACTAAGGGACAAATTCCCCCAAC |
| bacter_1_2 | TTCCGCGACTGTCATTCCACGTTCG | plancto_1_2 | GCAACACCTGTGCAGGTCACACCCG | gamma_5_2 | CGCCACTAAGGGACAAATTCCCCCA |
| bacter_1_3 | ACGTTTCCGCGACTGTCATTCCACG | plancto_1_3 | ATGCAACACCTGTGCAGGTCACACC | gamma_5_3 | GCCACTAAGGGACAAATTCCCCCAA |
| bacter_1_4 | TTTCCGCGACTGTCATTCCACGTTC | plancto_1_4 | AACACCTGTGCAGGTCACACCCGAA | gamma_5_4 | CACTAAGGGACAAATTCCCCCAACG |
| bacter_1_5 | CACGTTTCCGCGACTGTCATTCCAC | plancto_1_5 | CAACACCTGTGCAGGTCACACCCGA | gamma_5_5 | ACTAAGGGACAAATTCCCCCAACGG |
| bacter_1_6 | TCACGTTTCCGCGACTGTCATTCCA | plancto_1_6 | TGTGCAGGTCACACCCGAAGGTAAT | gamma_5_6 | CTAAGGGACAAATTCCCCCAACGGC |
| bacter_1_7 | CGTTTCCGCGACTGTCATTCCACGT | plancto_1_7 | GTGCAGGTCACACCCGAAGGTAATC | gamma_5_7 | GCGCCACTAAGGGACAAATTCCCCC |
| bacter_1_8 | TGTCATTCCACGTTCGAGCCCAGGT | plancto_1_8 | TGCAGGTCACACCCGAAGGTAATCA | gamma_5_8 | GGTACCGTCAAGACGCGCAGTTATT |
| bacter_1_9 | CTGTCATTCCACGTTCGAGCCCAGG | plancto_1_9 | CTGTGCAGGTCACACCCGAAGGTAA | gamma_5_9 | AGGTACCGTCAAGACGCGCAGTTAT |
| bacter_1_10 | CCGCGACTGTCATTCCACGTTCGAG | plancto_1_10 | CCTGTGCAGGTCACACCCGAAGGTA | gamma_5_10 | TAGGTACCGTCAAGACGCGCAGTTA |
| bacter_1_11 | ACTGTCATTCCACGTTCGAGCCCAG | plancto_1_11 | ACACCTGTGCAGGTCACACCCGAAG | gamma_5_11 | TGCGCCACTAAGGGACAAATTCCCC |
| bacter_1_12 | CGCGACTGTCATTCCACGTTCGAGC | plancto_1_12 | ACAGAGTTAGCCAGTGCTTCCTCTC | gamma_5_12 | TAAGGGACAAATTCCCCCAACGGCT |
| bacter_1_13 | GCGACTGTCATTCCACGTTCGAGCC | plancto_1_13 | ACCTGTGCAGGTCACACCCGAAGGT | gamma_5_13 | CTGTAGGTACCGTCAAGACGCGCAG |
| bacter_1_14 | CGACTGTCATTCCACGTTCGAGCCC | plancto_1_14 | CATGCAACACCTGTGCAGGTCACAC | gamma_5_14 | GTAGGTACCGTCAAGACGCGCAGTT |
| bacter_1_15 | TCCGCGACTGTCATTCCACGTTCGA | plancto_1_15 | CACCTGTGCAGGTCACACCCGAAGG | gamma_5_15 | CTGCGCCACTAAGGGACAAATTCCC |
| bacter_1_16 | GACTGTCATTCCACGTTCGAGCCCA | plancto_1_16 | CACAGAGTTAGCCAGTGCTTCCTCT | gamma_5_16 | TGTAGGTACCGTCAAGACGCGCAGT |
| bacter_1_17 | ATCACGTTTCCGCGACTGTCATTCC | plancto_1_17 | CAGAGTTAGCCAGTGCTTCCTCTCG | gamma_5_17 | TCTGTAGGTACCGTCAAGACGCGCA |
| bacter_1_18 | GTCATTCCACGTTCGAGCCCAGGTA | plancto_1_18 | AGCCAGTGCTTCCTCTCGAGCTTAC | gamma_5_18 | GTCCGCCACTCGACGCCTGAAGAGC |
| bacter_1_19 | ACGGTACCATCAGCACCGATACACG | plancto_1_19 | GCACAGAGTTAGCCAGTGCTTCCTC | gamma_5_19 | GCCACTCGACGCCTGAAGAGCAAGC |
| bacter_1_20 | GTACCATCAGCACCGATACACGACC | plancto_1_20 | GGCCTAGCCCCTGCATGTCAAGCCT | gamma_5_20 | GCTGCGCCACTAAGGGACAAATTCC |
| bacter_1_21 | GGTACCATCAGCACCGATACACGAC | plancto_1_21 | GCAGGTCACACCCGAAGGTAATCAG | gamma_5_21 | CACTCGGTTCCCGAAGGCACCAAAC |
| bacter_1_22 | CGGTACCATCAGCACCGATACACGA | plancto_1_22 | ACCGGCCTAGCCCCTGCATGTCAAG | gamma_5_22 | CTTCTGTAGGTACCGTCAAGACGCG |
| bacter_1_23 | GATCACGTTTCCGCGACTGTCATTC | plancto_1_23 | CAGGTCACACCCGAAGGTAATCAGC | gamma_5_23 | CACTCGACGCCTGAAGAGCAAGCTC |
| bacter_1_24 | TACGGTACCATCAGCACCGATACAC | plancto_1_24 | CCGGCCTAGCCCCTGCATGTCAAGC | gamma_5_24 | CGCCACTCGACGCCTGAAGAGCAAG |
| bacter_1_25 | CACCGATACACGACCGGTGGTTTTT | plancto_1_25 | CGGCCTAGCCCCTGCATGTCAAGCC | gamma_5_25 | GGACAAATTCCCCCAACGGCTAGTT |
| bacter_2_1 | GGATTTCTCCGGGCTACCTTCCGGT | plancto_2_1 | TCTCCGAAGAGCACTCTCCCCTTTC | gamma_6_1 | AGCTGCGCCACCAACCTCTTGAATG |
| bacter_2_2 | CTCCGGGCTACCTTCCGGTAAAGGG | plancto_2_2 | TACGACCGAGAAACTGTGGGAGGTC | gamma_6_2 | CCAACCTCTTGAATGAGGCCGACGG |
| bacter_2_3 | CGGATTTCTCCGGGCTACCTTCCGG | plancto_2_3 | ACCGAGAAACTGTGGGAGGTCCCTC | gamma_6_3 | TGCGCCACCAACCTCTTGAATGAGG |
| bacter_2_4 | TCTCCGGGCTACCTTCCGGTAAAGG | plancto_2_4 | CGACCGAGAAACTGTGGGAGGTCCC | gamma_6_4 | GCCACCAACCTCTTGAATGAGGCCG |
| bacter_2_5 | TTCTCCGGGCTACCTTCCGGTAAAG | plancto_2_5 | CTCCGAAGAGCACTCTCCCCTTTCA | gamma_6_5 | ACCAACCTCTTGAATGAGGCCGACG |
| bacter_2_6 | TTTCTCCGGGCTACCTTCCGGTAAA | plancto_2_6 | GCCCGACCTTCCTCTGAGGTTTGGT | gamma_6_6 | CTGCGCCACCAACCTCTTGAATGAG |
| bacter_2_7 | GATTTCTCCGGGCTACCTTCCGGTA | plancto_2_7 | AAACTGTGGGAGGTCCCTCGATCCA | gamma_6_7 | CAACCTCTTGAATGAGGCCGACGGC |
| bacter_2_8 | ATTTCTCCGGGCTACCTTCCGGTAA | plancto_2_8 | TCCGAAGAGCACTCTCCCCTTTCAG | gamma_6_8 | GCGCCACCAACCTCTTGAATGAGGC |
| bacter_2_9 | CCGGATTTCTCCGGGCTACCTTCCG | plancto_2_9 | GACCGAGAAACTGTGGGAGGTCCCT | gamma_6_9 | CGCCACCAACCTCTTGAATGAGGCC |
| bacter_2_10 | TCCGGATTTCTCCGGGCTACCTTCC | plancto_2_10 | ACGACCGAGAAACTGTGGGAGGTCC | gamma_6_10 | CACCAACCTCTTGAATGAGGCCGAC |
| bacter_2_11 | TCCGGGCTACCTTCCGGTAAAGGGT | plancto_2_11 | GAAACTGTGGGAGGTCCCTCGATCC | gamma_6_11 | GCTGCGCCACCAACCTCTTGAATGA |
| bacter_2_12 | ATCCGGATTTCTCCGGGCTACCTTC | plancto_2_12 | CTCTCCGAAGAGCACTCTCCCCTTT | gamma_6_12 | CCACCAACCTCTTGAATGAGGCCGA |
| bacter_2_13 | CTTTATGGATTAGCTCCCCGTCGCT | plancto_2_13 | GCCTGGAGGTAGGTATCTACCTGTT | gamma_6_13 | TAGCTGCGCCACCAACCTCTTGAAT |
| bacter_2_14 | ACTTTATGGATTAGCTCCCCGTCGC | plancto_2_14 | TCCCGACGCTATTCCCAGCCTGGAG | gamma_6_14 | AACCTCTTGAATGAGGCCGACGGCT |
| bacter_2_15 | CCGGGCTACCTTCCGGTAAAGGGTA | plancto_2_15 | TTGGGCATTACCGCCAGTTTCCCGA | gamma_6_15 | AGAGGTCCACTTTGCCCCGAAGGGC |
| bacter_2_16 | AATCCGGATTTCTCCGGGCTACCTT | plancto_2_16 | CCGAGAAACTGTGGGAGGTCCCTCG | gamma_6_16 | GAGGTCCACTTTGCCCCGAAGGGCG |
| bacter_2_17 | GCTACCTTCCGGTAAAGGGTAGGTT | plancto_2_17 | TGAGCAGACCCATCTCCAGGCGCCG | gamma_6_17 | TCTTCAGGTAACGTCAATACGCGCG |
| bacter_2_18 | GGCTACCTTCCGGTAAAGGGTAGGT | plancto_2_18 | AACTGTGGGAGGTCCCTCGATCCAG | gamma_6_18 | TTAGCTGCGCCACCAACCTCTTGAA |
| bacter_2_19 | GGGCTACCTTCCGGTAAAGGGTAGG | plancto_2_19 | CCCGACCTTCCTCTGAGGTTTGGTC | gamma_6_19 | CAGAGGTCCACTTTGCCCCGAAGGG |
| bacter_2_20 | TAATCCGGATTTCTCCGGGCTACCT | plancto_2_20 | TGGGCATTACCGCCAGTTTCCCGAC | gamma_6_20 | AGGTCCACTTTGCCCCGAAGGGCGT |
| bacter_2_21 | CTACCTTCCGGTAAAGGGTAGGTTG | plancto_2_21 | CGAGAAACTGTGGGAGGTCCCTCGA | gamma_6_21 | ACCTCTTGAATGAGGCCGACGGCTA |
| bacter_2_22 | CGGGCTACCTTCCGGTAAAGGGTAG | plancto_2_22 | GAGAAACTGTGGGAGGTCCCTCGAT | gamma_6_22 | CGCGCGGGTATTAACCGCACGCTTT |
| bacter_2_23 | TTAATCCGGATTTCTCCGGGCTACC | plancto_2_23 | CAGCCTGGAGGTAGGTATCTACCTG | gamma_6_23 | CTTCAGGTAACGTCAATACGCGCGG |
| bacter_2_24 | TTTATGGATTAGCTCCCCGTCGCTG | plancto_2_24 | AGCCCGACCTTCCTCTGAGGTTTGG | gamma_6_24 | TCAGAGGTCCACTTTGCCCCGAAGG |
| bacter_2_25 | TACCTTCCGGTAAAGGGTAGGTTGC | plancto_2_25 | AATAGTGAGCAGACCCATCTCCAGG | gamma_6_25 | ACGCGCGGGTATTAACCGCACGCTT |
| bacter_3_1 | GGCTCCTCGCCGTATCATCGAAATT | plancto_3_1 | CGCAGTGCCTCAGTTAAGCTCAGGC | gamma_7_1 | GTCCTCCGTAGTTAGACTAGCCACT |
| bacter_3_2 | CAACCTTGCCAATCACTCCCCAGGT | plancto_3_2 | GCAGTGCCTCAGTTAAGCTCAGGCA | gamma_7_2 | CGTCCTCCGTAGTTAGACTAGCCAC |
| bacter_3_3 | CTTGCCAATCACTCCCCAGGTGGAT | plancto_3_3 | CAACTCTGAGGGAGTACCCTCAGAG | gamma_7_3 | ACCGTCCTCCGTAGTTAGACTAGCC |
| bacter_3_4 | CAGGTAAGGCTCCTCGCCGTATCAT | plancto_3_4 | GTCAACTCTGAGGGAGTACCCTCAG | gamma_7_4 | CCGTCCTCCGTAGTTAGACTAGCCA |
| bacter_3_5 | AGGCTCCTCGCCGTATCATCGAAAT | plancto_3_5 | TATGTTTTCCTACGCCGTTCGCCGC | gamma_7_5 | GACCGTCCTCCGTAGTTAGACTAGC |
| bacter_3_6 | AACCTTGCCAATCACTCCCCAGGTG | plancto_3_6 | GCAGAAAGAGGAAACCTCCTCCCGC | gamma_7_6 | TGACCGTCCTCCGTAGTTAGACTAG |
| bacter_3_7 | ACCTTGCCAATCACTCCCCAGGTGG | plancto_3_7 | AACTCTGAGGGAGTACCCTCAGAGA | gamma_7_7 | CTGCAGGTAACGTCAAGTACTCACC |
| bacter_3_8 | TCAACCTTGCCAATCACTCCCCAGG | plancto_3_8 | TCAACTCTGAGGGAGTACCCTCAGA | gamma_7_8 | TATTAGGGGTAAGCCTTCCTCCCTG |
| bacter_3_9 | GGTAAGGCTCCTCGCCGTATCATCG | plancto_3_9 | CTATGTTTTCCTACGCCGTTCGCCG | gamma_7_9 | TGCAGGTAACGTCAAGTACTCACCC |
| bacter_3_10 | TCCGCCTACCCCAACTATACTCTAG | plancto_3_10 | TCCTATGTTTTCCTACGCCGTTCGC | gamma_7_10 | GCAGGTAACGTCAAGTACTCACCCG |
| bacter_3_11 | TTCAACCTTGCCAATCACTCCCCAG | plancto_3_11 | CCTATGTTTTCCTACGCCGTTCGCC | gamma_7_11 | TTCCCCGGGTTGTCCCCCACTCATG |
| bacter_3_12 | CCCAGGTAAGGCTCCTCGCCGTATC | plancto_3_12 | ACTCTGAGGGAGTACCCTCAGAGAT | gamma_7_12 | TCCCCGGGTTGTCCCCCACTCATGG |
| bacter_3_13 | AGGTAAGGCTCCTCGCCGTATCATC | plancto_3_13 | ACGCAGTGCCTCAGTTAAGCTCAGG | gamma_7_13 | CCCCGGGTTGTCCCCCACTCATGGG |
| bacter_3_14 | CCAATCACTCCCCAGGTGGATTACC | plancto_3_14 | TGTCAACTCTGAGGGAGTACCCTCA | gamma_7_14 | TTTCCCCGGGTTGTCCCCCACTCAT |
| bacter_3_15 | CCTTGCCAATCACTCCCCAGGTGGA | plancto_3_15 | ATGTTTTCCTACGCCGTTCGCCGCT | gamma_7_15 | CCCGGGTTGTCCCCCACTCATGGGT |
| bacter_3_16 | GTAAGGCTCCTCGCCGTATCATCGA | plancto_3_16 | AACGCAGTGCCTCAGTTAAGCTCAG | gamma_7_16 | CCGGGTTGTCCCCCACTCATGGGTA |
| bacter_3_17 | CCGCCTACCCCAACTATACTCTAGA | plancto_3_17 | CAGTGCCTCAGTTAAGCTCAGGCAT | gamma_7_17 | CTCACCCGTATTAGGGGTAAGCCTT |
| bacter_3_18 | CCAGGTAAGGCTCCTCGCCGTATCA | plancto_3_18 | CTGTCAACTCTGAGGGAGTACCCTC | gamma_7_18 | ACCCGTATTAGGGGTAAGCCTTCCT |
| bacter_3_19 | AAGGCTCCTCGCCGTATCATCGAAA | plancto_3_19 | CTCTGAGGGAGTACCCTCAGAGATT | gamma_7_19 | ACTCACCCGTATTAGGGGTAAGCCT |
| bacter_3_20 | GCCAATCACTCCCCAGGTGGATTAC | plancto_3_20 | TCTGTCAACTCTGAGGGAGTACCCT | gamma_7_20 | GTCAAGTACTCACCCGTATTAGGGG |
| bacter_3_21 | TAAGGCTCCTCGCCGTATCATCGAA | plancto_3_21 | GGAGTACCCTCAGAGATTTCATCCC | gamma_7_21 | TCACCCGTATTAGGGGTAAGCCTTC |
| bacter_3_22 | GCCCAGGTAAGGCTCCTCGCCGTAT | plancto_3_22 | CAAACGCAGTGCCTCAGTTAAGCTC | gamma_7_22 | CCCGTATTAGGGGTAAGCCTTCCTC |
| bacter_3_23 | CATTCCGCCTACCCCAACTATACTC | plancto_3_23 | CTCTGTCAACTCTGAGGGAGTACCC | gamma_7_23 | GTACTCACCCGTATTAGGGGTAAGC |
| bacter_3_24 | CAATCACTCCCCAGGTGGATTACCT | plancto_3_24 | ACAGCAGAAAGAGGAAACCTCCTCC | gamma_7_24 | CACCCGTATTAGGGGTAAGCCTTCC |
| bacter_3_25 | CCGCCGGAACTTTGATCATCAAGAG | plancto_3_25 | CTGAGGGAGTACCCTCAGAGATTTC | gamma_7_25 | TACTCACCCGTATTAGGGGTAAGCC |
| flavo_1_1 | CTCAGACACCAAGGTCCAAACAGCT | plancto_4_1 | ACTACCTAATATCGCATCGGCCGCT | gamma_8_1 | CGCGAGCTCATCCATCAGCACAAGG |
| flavo_1_2 | CAGACACCAAGGTCCAAACAGCTAG | plancto_4_2 | CAACTACCTAATATCGCATCGGCCG | gamma_8_2 | TCATCCATCAGCACAAGGTCCGAAG |
| flavo_1_3 | CACTCAGACACCAAGGTCCAAACAG | plancto_4_3 | AACTACCTAATATCGCATCGGCCGC | gamma_8_3 | CTCATCCATCAGCACAAGGTCCGAA |
| flavo_1_4 | GCTTAGCCACTCAGACACCAAGGTC | plancto_4_4 | CCAACTACCTAATATCGCATCGGCC | gamma_8_4 | GCTCATCCATCAGCACAAGGTCCGA |
| flavo_1_5 | ACTCAGACACCAAGGTCCAAACAGC | plancto_4_5 | ACGTTCCGATGTATTCCTACCCCGT | gamma_8_5 | ACGCGAGCTCATCCATCAGCACAAG |
| flavo_1_6 | CTTAGCCACTCAGACACCAAGGTCC | plancto_4_6 | TACGTTCCGATGTATTCCTACCCCG | gamma_8_6 | CATCCATCAGCACAAGGTCCGAAGA |
| flavo_1_7 | TACCGTCAAGCTTGGTACACGTACC | plancto_4_7 | GTACGTTCCGATGTATTCCTACCCC | gamma_8_7 | GACGCGAGCTCATCCATCAGCACAA |
| flavo_1_8 | GTACCGTCAAGCTTGGTACACGTAC | plancto_4_8 | CTACCTAATATCGCATCGGCCGCTC | gamma_8_8 | GCGAGCTCATCCATCAGCACAAGGT |
| flavo_1_9 | GCCACTCAGACACCAAGGTCCAAAC | plancto_4_9 | CGTTCCGATGTATTCCTACCCCGTT | gamma_8_9 | TCCATCAGCACAAGGTCCGAAGATC |
| flavo_1_10 | TTAGCCACTCAGACACCAAGGTCCA | plancto_4_10 | GTTTCCACCCACTAATCCGTGCATG | gamma_8_10 | CGACGCGAGCTCATCCATCAGCACA |
| flavo_1_11 | ACCGTCAAGCTTGGTACACGTACCA | plancto_4_11 | TTCCACCCACTAATCCGTGCATGTC | gamma_8_11 | CATCAGCACAAGGTCCGAAGATCCC |
| flavo_1_12 | CCACTCAGACACCAAGGTCCAAACA | plancto_4_12 | TCCACCCACTAATCCGTGCATGTCA | gamma_8_12 | CCCTCTAATGGGCAGATTCTCACGT |
| flavo_1_13 | AGCCACTCAGACACCAAGGTCCAAA | plancto_4_13 | CCACCCACTAATCCGTGCATGTCAA | gamma_8_13 | CCGACGCGAGCTCATCCATCAGCAC |
| flavo_1_14 | TAGCCACTCAGACACCAAGGTCCAA | plancto_4_14 | GGCAGTAAACCTTTGGTCTCTCGAC | gamma_8_14 | CCCCTCTAATGGGCAGATTCTCACG |
| flavo_1_15 | CCGTCAAGCTTGGTACACGTACCAA | plancto_4_15 | GGTACGTTCCGATGTATTCCTACCC | gamma_8_15 | CCCCCTCTAATGGGCAGATTCTCAC |
| flavo_1_16 | CGCTTAGCCACTCAGACACCAAGGT | plancto_4_16 | TGCGAGCGTCATGAATGTTTCCACC | gamma_8_16 | CGAGCTCATCCATCAGCACAAGGTC |
| flavo_1_17 | TCGCTTAGCCACTCAGACACCAAGG | plancto_4_17 | GCGAGCGTCATGAATGTTTCCACCC | gamma_8_17 | CCATCAGCACAAGGTCCGAAGATCC |
| flavo_1_18 | CGTCAAGCTTGGTACACGTACCAAG | plancto_4_18 | GAGCGTCATGAATGTTTCCACCCAC | gamma_8_18 | CCTCTAATGGGCAGATTCTCACGTG |
| flavo_1_19 | CAGCTAGTAACCATCGTTTACCGGC | plancto_4_19 | CGAGCGTCATGAATGTTTCCACCCA | gamma_8_19 | CCCAGGTTATCCCCCTCTAATGGGC |
| flavo_1_20 | GCCATAGCTAGAGACTATGGGGGAT | plancto_4_20 | CAGTTATGCCCCAGTGAATCGCCTT | gamma_8_20 | TCCGACGCGAGCTCATCCATCAGCA |
| flavo_1_21 | TGCCATAGCTAGAGACTATGGGGGA | plancto_4_21 | TCAGTTATGCCCCAGTGAATCGCCT | gamma_8_21 | GAGCTCATCCATCAGCACAAGGTCC |
| flavo_1_22 | ATGCCATAGCTAGAGACTATGGGGG | plancto_4_22 | AGTTATGCCCCAGTGAATCGCCTTC | gamma_8_22 | TTCCCCAGGTTATCCCCCTCTAATG |
| flavo_1_23 | TTCGCTTAGCCACTCAGACACCAAG | plancto_4_23 | GTCAGTTATGCCCCAGTGAATCGCC | gamma_8_23 | TCCCCAGGTTATCCCCCTCTAATGG |
| flavo_1_24 | AGCTAGTAACCATCGTTTACCGGCG | plancto_4_24 | GTTATGCCCCAGTGAATCGCCTTCG | gamma_8_24 | CCCCAGGTTATCCCCCTCTAATGGG |
| flavo_1_25 | GTCAAGCTTGGTACACGTACCAAGG | plancto_4_25 | CTCCACTGGATGTTCCATTCACCTC | gamma_8_25 | ATCCCCCTCTAATGGGCAGATTCTC |
| flavo_2_1 | TACAGTACCGTCAGAGCTCTACACG | alpha_1_1 | CCGGCCCCTTGCGGGAAGAAAGCCA | gamma_9_1 | CCTGTCCATCGGTTCCCGAAGGCAC |
| flavo_2_2 | TCTTACAGTACCGTCAGAGCTCTAC | alpha_1_2 | CACCTGTGCACCGGCCCCTTGCGGG | gamma_9_2 | CTGTCCATCGGTTCCCGAAGGCACC |
| flavo_2_3 | TTACAGTACCGTCAGAGCTCTACAC | alpha_1_3 | GCACCTGTGCACCGGCCCCTTGCGG | gamma_9_3 | TGTCCATCGGTTCCCGAAGGCACCA |
| flavo_2_4 | GCATACTCATCTCTTACCGCCGAAG | alpha_1_4 | CTGTGCACCGGCCCCTTGCGGGAAG | gamma_9_4 | CAGCACCTGTCCATCGGTTCCCGAA |
| flavo_2_5 | CATACTCATCTCTTACCGCCGAAGC | alpha_1_5 | ACCTGTGCACCGGCCCCTTGCGGGA | gamma_9_5 | AGCACCTGTCCATCGGTTCCCGAAG |
| flavo_2_6 | ACAGTACCGTCAGAGCTCTACACGT | alpha_1_6 | CCTGTGCACCGGCCCCTTGCGGGAA | gamma_9_6 | ACCTGTCCATCGGTTCCCGAAGGCA |
| flavo_2_7 | CAGTACCGTCAGAGCTCTACACGTA | alpha_1_7 | AGCACCTGTGCACCGGCCCCTTGCG | gamma_9_7 | GTCCATCGGTTCCCGAAGGCACCAA |
| flavo_2_8 | CTTACAGTACCGTCAGAGCTCTACA | alpha_1_8 | CGGCCCCTTGCGGGAAGAAAGCCAT | gamma_9_8 | CACCTGTCCATCGGTTCCCGAAGGC |
| flavo_2_9 | TACTCATCTCTTACCGCCGAAGCTT | alpha_1_9 | GCACCGGCCCCTTGCGGGAAGAAAG | gamma_9_9 | CCTCCCTCTCTCGCACTCTAGCCTT |
| flavo_2_10 | ATACTCATCTCTTACCGCCGAAGCT | alpha_1_10 | CACCGGCCCCTTGCGGGAAGAAAGC | gamma_9_10 | GCACCTGTCCATCGGTTCCCGAAGG |
| flavo_2_11 | CTCATCTCTTACCGCCGAAGCTTTA | alpha_1_11 | ACCGGCCCCTTGCGGGAAGAAAGCC | gamma_9_11 | GCAGCACCTGTCCATCGGTTCCCGA |
| flavo_2_12 | CGCCCAGTGGCTGCTCTCTGTCTAT | alpha_1_12 | TGTGCACCGGCCCCTTGCGGGAAGA | gamma_9_12 | ACCTCCCTCTCTCGCACTCTAGCCT |
| flavo_2_13 | CCAGTGGCTGCTCTCTGTCTATACC | alpha_1_13 | GTGCACCGGCCCCTTGCGGGAAGAA | gamma_9_13 | CTCCCTCTCTCGCACTCTAGCCTTC |
| flavo_2_14 | CCCAGTGGCTGCTCTCTGTCTATAC | alpha_1_14 | TGCACCGGCCCCTTGCGGGAAGAAA | gamma_9_14 | TCTCTCGCACTCTAGCCTTCCAGTA |
| flavo_2_15 | TCGCCCAGTGGCTGCTCTCTGTCTA | alpha_1_15 | CAGCACCTGTGCACCGGCCCCTTGC | gamma_9_15 | TCGCACTCTAGCCTTCCAGTATCGG |
| flavo_2_16 | GCCCAGTGGCTGCTCTCTGTCTATA | alpha_1_16 | TTGCGGGAAGAAAGCCATCTCTGGC | gamma_9_16 | CTCGCACTCTAGCCTTCCAGTATCG |
| flavo_2_17 | GACTCCGATCCGAACTGTGATATAG | alpha_1_17 | GGCCCCTTGCGGGAAGAAAGCCATC | gamma_9_17 | TACCTCCCTCTCTCGCACTCTAGCC |
| flavo_2_18 | AGAACGCATACTCATCTCTTACCGC | alpha_1_18 | CCTTGCGGGAAGAAAGCCATCTCTG | gamma_9_18 | CTCTCGCACTCTAGCCTTCCAGTAT |
| flavo_2_19 | GAACGCATACTCATCTCTTACCGCC | alpha_1_19 | GCAGCACCTGTGCACCGGCCCCTTG | gamma_9_19 | CCCTCTCTCGCACTCTAGCCTTCCA |
| flavo_2_20 | CACGTAGAGCGGTTTCTTCCTGTAT | alpha_1_20 | TGCGGGAAGAAAGCCATCTCTGGCG | gamma_9_20 | TGCAGCACCTGTCCATCGGTTCCCG |
| flavo_2_21 | GTCCTGTCACACTACATTTAAGCCC | alpha_1_21 | AAAGCCATCTCTGGCGATCATACCG | gamma_9_21 | ACTCCGTGGTAATCGCCCTCCCGAA |
| flavo_2_22 | ACTCATCTCTTACCGCCGAAGCTTT | alpha_1_22 | GCCCCTTGCGGGAAGAAAGCCATCT | gamma_9_22 | TCCATCGGTTCCCGAAGGCACCAAT |
| flavo_2_23 | CCCCTATCTATCGTAGCCATGGTGT | alpha_1_23 | AACAGCAAGCTGCCCAACGGCTAGC | gamma_9_23 | TCACTCCGTGGTAATCGCCCTCCCG |
| flavo_2_24 | CCCTATCTATCGTAGCCATGGTGTG | alpha_1_24 | CATGCAGCACCTGTGCACCGGCCCC | gamma_9_24 | TCCCTCTCTCGCACTCTAGCCTTCC |
| flavo_2_25 | CCTATCTATCGTAGCCATGGTGTGC | alpha_1_25 | GCAAGCTGCCCAACGGCTAGCATCC | gamma_9_25 | CCTCTCTCGCACTCTAGCCTTCCAG |
| flavo_3_1 | CTGTCACCTAACATTTAAGCCCTGG | alpha_2_1 | GTGACCCAGAAAGTTGCCTTCGCAT | gamma_10_1 | CGCAGGCACATCCGATAGCGAGAGC |
| flavo_3_2 | CCGTCAAGCTTTCTCACGAGAAAGT | alpha_2_2 | GTATTCACCGCGACGCGCTGATTCG | gamma_10_2 | ACGCAGGCACATCCGATAGCGAGAG |
| flavo_3_3 | ACCGTCAAGCTTTCTCACGAGAAAG | alpha_2_3 | CGTATTCACCGCGACGCGCTGATTC | gamma_10_3 | GCGGCTTCGCGGCCCTCTGTACTTG |
| flavo_3_4 | CTCTGACTTATTTGTCCACCTACGG | alpha_2_4 | TATTCACCGCGACGCGCTGATTCGC | gamma_10_4 | CGGCTTCGCGGCCCTCTGTACTTGC |
| flavo_3_5 | CCTCTGACTTATTTGTCCACCTACG | alpha_2_5 | ACGTATTCACCGCGACGCGCTGATT | gamma_10_5 | GGCTTCGCGGCCCTCTGTACTTGCC |
| flavo_3_6 | GTACCGTCAAGCTTTCTCACGAGAA | alpha_2_6 | GGAACGTATTCACCGCGACGCGCTG | gamma_10_6 | CGCGGCTTCGCGGCCCTCTGTACTT |
| flavo_3_7 | GAGGCAGATTGTATACGCGATACTC | alpha_2_7 | CCGGGAACGTATTCACCGCGACGCG | gamma_10_7 | GCTTCGCGGCCCTCTGTACTTGCCA |
| flavo_3_8 | TCTATCGTAGCCTAGGTGTGCCGTT | alpha_2_8 | CGGGAACGTATTCACCGCGACGCGC | gamma_10_8 | CACTACTGGGTAGTTTCCTACGCGT |
| flavo_3_9 | CCCCTATCTATCGTAGCCTAGGTGT | alpha_2_9 | GGGAACGTATTCACCGCGACGCGCT | gamma_10_9 | CCACTACTGGGTAGTTTCCTACGCG |
| flavo_3_10 | ATCTATCGTAGCCTAGGTGTGCCGT | alpha_2_10 | AACGTATTCACCGCGACGCGCTGAT | gamma_10_10 | CCCCACTACTGGGTAGTTTCCTACG |
| flavo_3_11 | CCCTATCTATCGTAGCCTAGGTGTG | alpha_2_11 | GAACGTATTCACCGCGACGCGCTGA | gamma_10_11 | CCCACTACTGGGTAGTTTCCTACGC |
| flavo_3_12 | TATCTATCGTAGCCTAGGTGTGCCG | alpha_2_12 | CCCGGGAACGTATTCACCGCGACGC | gamma_10_12 | CCCCCACTACTGGGTAGTTTCCTAC |
| flavo_3_13 | CCTATCTATCGTAGCCTAGGTGTGC | alpha_2_13 | ATTCACCGCGACGCGCTGATTCGCG | gamma_10_13 | ACTACCGGGTAGTTTCCTACGCGTT |
| flavo_3_14 | CTATCTATCGTAGCCTAGGTGTGCC | alpha_2_14 | CCGCGACGCGCTGATTCGCGATTAC | gamma_10_14 | CACTACCGGGTAGTTTCCTACGCGT |
| flavo_3_15 | CTATCGTAGCCTAGGTGTGCCGTTA | alpha_2_15 | CACCGCGACGCGCTGATTCGCGATT | gamma_10_15 | ACCGGGTAGTTTCCTACGCGTTACT |
| flavo_3_16 | TATCGTAGCCTAGGTGTGCCGTTAC | alpha_2_16 | CGCGACGCGCTGATTCGCGATTACT | gamma_10_16 | CCACTACCGGGTAGTTTCCTACGCG |
| flavo_3_17 | CTTATTTGTCCACCTACGGACCCTT | alpha_2_17 | TCACCGCGACGCGCTGATTCGCGAT | gamma_10_17 | CCCCACTACCGGGTAGTTTCCTACG |
| flavo_3_18 | ACTTATTTGTCCACCTACGGACCCT | alpha_2_18 | ACCGCGACGCGCTGATTCGCGATTA | gamma_10_18 | CCGGGTAGTTTCCTACGCGTTACTC |
| flavo_3_19 | GACTTATTTGTCCACCTACGGACCC | alpha_2_19 | GCGACGCGCTGATTCGCGATTACTA | gamma_10_19 | CCCACTACCGGGTAGTTTCCTACGC |
| flavo_3_20 | TGACTTATTTGTCCACCTACGGACC | alpha_2_20 | TTCACCGCGACGCGCTGATTCGCGA | gamma_10_20 | TACCGGGTAGTTTCCTACGCGTTAC |
| flavo_3_21 | CTGACTTATTTGTCCACCTACGGAC | alpha_2_21 | TCCTCAGTGTCAGTAGTGACCCAGA | gamma_10_21 | CCCCCACTACCGGGTAGTTTCCTAC |
| flavo_3_22 | AGATTGTATACGCGATACTCACCCG | alpha_2_22 | CCCAGAAAGTTGCCTTCGCATTTGG | gamma_10_22 | CTACCGGGTAGTTTCCTACGCGTTA |
| flavo_3_23 | GATTGTATACGCGATACTCACCCGT | alpha_2_23 | AGTGCGGGCTCATCTTTCGGCGTAT | gamma_10_23 | CTGTTGTCCCCCACTACTGGGTAGT |
| flavo_3_24 | TCTTCGGGCTATTCCCTAGTATGAG | alpha_2_24 | AAGTGCGGGCTCATCTTTCGGCGTA | gamma_10_24 | CTAGCTAATCTCACGCAGGCACATC |
| flavo_3_25 | CTTCGGGCTATTCCCTAGTATGAGG | alpha_2_25 | GTGCGGGCTCATCTTTCGGCGTATA | gamma_10_25 | CAACTAGCTAATCTCACGCAGGCAC |
| flavo_4_1 | CAGGAGATATTCCCATACTATGGGG | alpha_3_1 | CACCTGTATCCAATCCACCCGAAGT | gamma_11_1 | GCTTTCCCCCGTAGGATATATGCGG |
| flavo_4_2 | TCAAACTCCCACACGTGGGAGTGGT | alpha_3_2 | ACCTGTATCCAATCCACCCGAAGTG | gamma_11_2 | CTTTCCCCCGTAGGATATATGCGGT |
| flavo_4_3 | CAAACTCCCACACGTGGGAGTGGTT | alpha_3_3 | CCTGTATCCAATCCACCCGAAGTGA | gamma_11_3 | TGCTTTCCCCCGTAGGATATATGCG |
| flavo_4_4 | GTCAAACTCCCACACGTGGGAGTGG | alpha_3_4 | GCACCTGTATCCAATCCACCCGAAG | gamma_11_4 | CTGCTTTCCCCCGTAGGATATATGC |
| flavo_4_5 | GGAGATATTCCCATACTATGGGGCA | alpha_3_5 | GGCAGTTCCTTCAAAGTTCCCACCA | gamma_11_5 | CCTGCTTTCCCCCGTAGGATATATG |
| flavo_4_6 | AGGAGATATTCCCATACTATGGGGC | alpha_3_6 | AGCACCTGTATCCAATCCACCCGAA | gamma_11_6 | CCCTGCTTTCCCCCGTAGGATATAT |
| flavo_4_7 | CGTCAAACTCCCACACGTGGGAGTG | alpha_3_7 | CGGCAGTTCCTTCAAAGTTCCCACC | gamma_11_7 | CTCACTCAGGCTCATCAAATAGCGC |
| flavo_4_8 | AAACTCCCACACGTGGGAGTGGTTC | alpha_3_8 | CAGCACCTGTATCCAATCCACCCGA | gamma_11_8 | CCCCTGCTTTCCCCCGTAGGATATA |
| flavo_4_9 | CTGGGCTATTCCCCTCCAAAAGGTA | alpha_3_9 | CCGGCAGTTCCTTCAAAGTTCCCAC | gamma_11_9 | GTGTCAGTATCGAGCCAGTCAGTCG |
| flavo_4_10 | CCGTCAAACTCCCACACGTGGGAGT | alpha_3_10 | GCAGCACCTGTATCCAATCCACCCG | gamma_11_10 | TCAGTGTCAGTATCGAGCCAGTCAG |
| flavo_4_11 | CTTAACCACTCAGCCCTTAATCGGG | alpha_3_11 | TGCAGCACCTGTATCCAATCCACCC | gamma_11_11 | AGTGTCAGTATCGAGCCAGTCAGTC |
| flavo_4_12 | GTTTCCCTGGGCTATTCCCCTCCAA | alpha_3_12 | TCACCGGCAGTTCCTTCAAAGTTCC | gamma_11_12 | TGTCAGTATCGAGCCAGTCAGTCGC |
| flavo_4_13 | GCTTAACCACTCAGCCCTTAATCGG | alpha_3_13 | CTTACAAATCCGCCTACGCTCGCTT | gamma_11_13 | CAGTGTCAGTATCGAGCCAGTCAGT |
| flavo_4_14 | AACTCCCACACGTGGGAGTGGTTCT | alpha_3_14 | ATGCAGCACCTGTATCCAATCCACC | gamma_11_14 | CTCAGTGTCAGTATCGAGCCAGTCA |
| flavo_4_15 | ACCGTCAAACTCCCACACGTGGGAG | alpha_3_15 | CGGGCCCATCCAATAGCGCATAAAG | gamma_11_15 | TCCCCTGCTTTCCCCCGTAGGATAT |
| flavo_4_16 | CCACACGTGGGAGTGGTTCTTCCTC | alpha_3_16 | GGGCCCATCCAATAGCGCATAAAGC | gamma_11_16 | CCCCACCAACTAGCTAATCTCACTC |
| flavo_4_17 | AGTTTCCCTGGGCTATTCCCCTCCA | alpha_3_17 | GCGGGCCCATCCAATAGCGCATAAA | gamma_11_17 | CCTCAGTGTCAGTATCGAGCCAGTC |
| flavo_4_18 | TTAACCACTCAGCCCTTAATCGGGC | alpha_3_18 | ACTTACAAATCCGCCTACGCTCGCT | gamma_11_18 | GTCCCCTGCTTTCCCCCGTAGGATA |
| flavo_4_19 | CACGTGGGAGTGGTTCTTCCTCTGT | alpha_3_19 | CGCGGGCCCATCCAATAGCGCATAA | gamma_11_19 | TCAGTATCGAGCCAGTCAGTCGCCT |
| flavo_4_20 | CACACGTGGGAGTGGTTCTTCCTCT | alpha_3_20 | GGCCCATCCAATAGCGCATAAAGCT | gamma_11_20 | GTATCGAGCCAGTCAGTCGCCTTCG |
| flavo_4_21 | ACACGTGGGAGTGGTTCTTCCTCTG | alpha_3_21 | CACCGGCAGTTCCTTCAAAGTTCCC | gamma_11_21 | AGTATCGAGCCAGTCAGTCGCCTTC |
| flavo_4_22 | CGCTTAACCACTCAGCCCTTAATCG | alpha_3_22 | ACCGGCAGTTCCTTCAAAGTTCCCA | gamma_11_22 | TATCGAGCCAGTCAGTCGCCTTCGC |
| flavo_4_23 | ACGTGGGAGTGGTTCTTCCTCTGTA | alpha_3_23 | AACTTACAAATCCGCCTACGCTCGC | gamma_11_23 | ATCGAGCCAGTCAGTCGCCTTCGCC |
| flavo_4_24 | TTTCCCTGGGCTATTCCCCTCCAAA | alpha_3_24 | CGCATAAAGCTTTCTCCCGAAGGAC | gamma_11_24 | GTCAGTATCGAGCCAGTCAGTCGCC |
| flavo_4_25 | TTCCCTGGGCTATTCCCCTCCAAAA | alpha_3_25 | CATGCAGCACCTGTATCCAATCCAC | gamma_11_25 | CAGTATCGAGCCAGTCAGTCGCCTT |
| flavo_5_1 | CGTCAACAGTTCACACGTGAACCTT | roseo_1_1 | CTCTGGAATCCGCGACAAGTATGTC | gamma_12_1 | CACTACCTGGTAGATTCCTACGCGT |
| flavo_5_2 | ACAGTACCGTCAACAGTTCACACGT | roseo_1_2 | TGCCCCTATAAATAGTTGGCGCACC | gamma_12_2 | CCACTACCTGGTAGATTCCTACGCG |
| flavo_5_3 | CCGTCAACAGTTCACACGTGAACCT | roseo_1_3 | CCCTATAAATAGTTGGCGCACCACC | gamma_12_3 | CCCACTACCTGGTAGATTCCTACGC |
| flavo_5_4 | CAGTACCGTCAACAGTTCACACGTG | roseo_1_4 | CCCCTATAAATAGTTGGCGCACCAC | gamma_12_4 | AACTGTTGTCCCCCACTACCTGGTA |
| flavo_5_5 | TACAGTACCGTCAACAGTTCACACG | roseo_1_5 | GCCCCTATAAATAGTTGGCGCACCA | gamma_12_5 | CAACTGTTGTCCCCCACTACCTGGT |
| flavo_5_6 | ACCGTCAACAGTTCACACGTGAACC | roseo_1_6 | CGTGGTTGGCTGCCCCTATAAATAG | gamma_12_6 | CCAACTGTTGTCCCCCACTACCTGG |
| flavo_5_7 | CTACAGTACCGTCAACAGTTCACAC | roseo_1_7 | CTGCCCCTATAAATAGTTGGCGCAC | gamma_12_7 | CCCCACTACCTGGTAGATTCCTACG |
| flavo_5_8 | TACCGTCAACAGTTCACACGTGAAC | roseo_1_8 | CCGTGGTTGGCTGCCCCTATAAATA | gamma_12_8 | CGGTATTGCAACCCTCTGTACGCCC |
| flavo_5_9 | AGTACCGTCAACAGTTCACACGTGA | roseo_1_9 | TGGCTGCCCCTATAAATAGTTGGCG | gamma_12_9 | ACTGTTGTCCCCCACTACCTGGTAG |
| flavo_5_10 | GTACCGTCAACAGTTCACACGTGAA | roseo_1_10 | GGCTGCCCCTATAAATAGTTGGCGC | gamma_12_10 | TCCAACTGTTGTCCCCCACTACCTG |
| flavo_5_11 | CCTACAGTACCGTCAACAGTTCACA | roseo_1_11 | GGAATCCGCGACAAGTATGTCAAGG | gamma_12_11 | CCCCCACTACCTGGTAGATTCCTAC |
| flavo_5_12 | TCCTACAGTACCGTCAACAGTTCAC | roseo_1_12 | GCTGCCCCTATAAATAGTTGGCGCA | gamma_12_12 | GCGGTATTGCAACCCTCTGTACGCC |
| flavo_5_13 | CCGAAGAAAAAGATGTTTCCACCCC | roseo_1_13 | ACCGTGGTTGGCTGCCCCTATAAAT | gamma_12_13 | GCGGTATCGCAACCCTCTGTACGTT |
| flavo_5_14 | CTCAGACCGCAATTAGTCCGAACAG | roseo_1_14 | CCATCTCTGGAATCCGCGACAAGTA | gamma_12_14 | TCTATCAGTTTGGGGTGCAGTTCCC |
| flavo_5_15 | TAGCCACTCAGACCGCAATTAGTCC | roseo_1_15 | ATAGTTGGCGCACCACCTTCGGGTA | gamma_12_15 | GTCTATCAGTTTGGGGTGCAGTTCC |
| flavo_5_16 | TTAGCCACTCAGACCGCAATTAGTC | roseo_1_16 | GGAATCCATCTCTGGAATCCGCGAC | gamma_12_16 | CTGTTGTCCCCCACTACCTGGTAGA |
| flavo_5_17 | ACTCAGACCGCAATTAGTCCGAACA | roseo_1_17 | TACCGTGGTTGGCTGCCCCTATAAA | gamma_12_17 | CTATCAGTTTGGGGTGCAGTTCCCA |
| flavo_5_18 | AGATGTTTCCACCCCTGTCAAACTG | roseo_1_18 | GAATCCGCGACAAGTATGTCAAGGG | gamma_12_18 | CTGTTGCTAACGTCACAGCTAAGGG |
| flavo_5_19 | CAGACCGCAATTAGTCCGAACAGCT | roseo_1_19 | TCCATCTCTGGAATCCGCGACAAGT | gamma_12_19 | CAGTTTGGGGTGCAGTTCCCAGGTT |
| flavo_5_20 | GCCACTCAGACCGCAATTAGTCCGA | roseo_1_20 | ATCCATCTCTGGAATCCGCGACAAG | gamma_12_20 | AGTTTGGGGTGCAGTTCCCAGGTTG |
| flavo_5_21 | CACTCAGACCGCAATTAGTCCGAAC | roseo_1_21 | TAGTTGGCGCACCACCTTCGGGTAG | gamma_12_21 | TTCCAACTGTTGTCCCCCACTACCT |
| flavo_5_22 | CTTAGCCACTCAGACCGCAATTAGT | roseo_1_22 | CCTACCGTGGTTGGCTGCCCCTATA | gamma_12_22 | TATCAGTTTGGGGTGCAGTTCCCAG |
| flavo_5_23 | AGCCACTCAGACCGCAATTAGTCCG | roseo_1_23 | CTACCGTGGTTGGCTGCCCCTATAA | gamma_12_23 | CGGTATCGCAACCCTCTGTACGTTC |
| flavo_5_24 | TCAGACCGCAATTAGTCCGAACAGC | roseo_1_24 | ACGTCGTCCACACCTTCCTCCGGCT | gamma_12_24 | CCCCACCAACTAACTAATCTCACGC |
| flavo_5_25 | ACTTTCGCTTAGCCACTCAGACCGC | roseo_1_25 | GACGTCGTCCACACCTTCCTCCGGC | gamma_12_25 | GTCAGCGACTAGCAAGCTAGTCCTG |
| flavo_6_1 | AGTGCCGGAGTTAAGCCCCTGCATT | roseo_2_1 | GTCACCGGGTCACCGAAGTGAAAAC | gamma_13_1 | CGCCACTGAAAGACATTGTCTCCCA |
| flavo_6_2 | GTGCCGGAGTTAAGCCCCTGCATTT | roseo_2_2 | ACCGGGTCACCGAAGTGAAAACCAG | gamma_13_2 | GCGCCACTGAAAGACATTGTCTCCC |
| flavo_6_3 | CAGTGCCGGAGTTAAGCCCCTGCAT | roseo_2_3 | CACCGGGTCACCGAAGTGAAAACCA | gamma_13_3 | TGCGCCACTGAAAGACATTGTCTCC |
| flavo_6_4 | TGCCGGAGTTAAGCCCCTGCATTTC | roseo_2_4 | TCACCGGGTCACCGAAGTGAAAACC | gamma_13_4 | TGTCAGTACAGATCCAGGAGGCCGC |
| flavo_6_5 | AGTTAAGCCCCTGCATTTCACCACT | roseo_2_5 | TGTCACCGGGTCACCGAAGTGAAAA | gamma_13_5 | GTGTCAGTACAGATCCAGGAGGCCG |
| flavo_6_6 | GCAGTGCCGGAGTTAAGCCCCTGCA | roseo_2_6 | CCGGGTCACCGAAGTGAAAACCAGA | gamma_13_6 | CTGCGCCACTGAAAGACATTGTCTC |
| flavo_6_7 | GTTAAGCCCCTGCATTTCACCACTG | roseo_2_7 | AGATCTCTCTGGCGGTCCCGGGATG | gamma_13_7 | CTTGGCTCCAAAAGGCACACTCTCA |
| flavo_6_8 | GGCAGTGCCGGAGTTAAGCCCCTGC | roseo_2_8 | ACCAGATCTCTCTGGCGGTCCCGGG | gamma_13_8 | GAGAGCTTCAAGAGAGGCCCTCTTT |
| flavo_6_9 | TGGCAGTGCCGGAGTTAAGCCCCTG | roseo_2_9 | AACCAGATCTCTCTGGCGGTCCCGG | gamma_13_9 | CGAGAGCTTCAAGAGAGGCCCTCTT |
| flavo_6_10 | GAGTTAAGCCCCTGCATTTCACCAC | roseo_2_10 | AAACCAGATCTCTCTGGCGGTCCCG | gamma_13_10 | GCGAGAGCTTCAAGAGAGGCCCTCT |
| flavo_6_11 | GCCGGAGTTAAGCCCCTGCATTTCA | roseo_2_11 | TCTCTGGCGGTCCCGGGATGTCAAG | gamma_13_11 | TAGCGAGAGCTTCAAGAGAGGCCCT |
| flavo_6_12 | ATGGCAGTGCCGGAGTTAAGCCCCT | roseo_2_12 | ATCTCTCTGGCGGTCCCGGGATGTC | gamma_13_12 | AGAGCTTCAAGAGAGGCCCTCTTTC |
| flavo_6_13 | TTAAGCCCCTGCATTTCACCACTGA | roseo_2_13 | GATCTCTCTGGCGGTCCCGGGATGT | gamma_13_13 | AGCGAGAGCTTCAAGAGAGGCCCTC |
| flavo_6_14 | GGAGTTAAGCCCCTGCATTTCACCA | roseo_2_14 | CAGATCTCTCTGGCGGTCCCGGGAT | gamma_13_14 | GTCAGTACAGATCCAGGAGGCCGCC |
| flavo_6_15 | CGGAGTTAAGCCCCTGCATTTCACC | roseo_2_15 | TCTGGCGGTCCCGGGATGTCAAGGG | gamma_13_15 | TCAGTACAGATCCAGGAGGCCGCCT |
| flavo_6_16 | CCCTGCATTTCACCACTGACTTATC | roseo_2_16 | CTCTGGCGGTCCCGGGATGTCAAGG | gamma_13_16 | CAGTACAGATCCAGGAGGCCGCCTT |
| flavo_6_17 | CAATGGCAGTGCCGGAGTTAAGCCC | roseo_2_17 | CCAGATCTCTCTGGCGGTCCCGGGA | gamma_13_17 | AGTACAGATCCAGGAGGCCGCCTTC |
| flavo_6_18 | TCAATGGCAGTGCCGGAGTTAAGCC | roseo_2_18 | TCTCTCTGGCGGTCCCGGGATGTCA | gamma_13_18 | GCTGCGCCACTGAAAGACATTGTCT |
| flavo_6_19 | CCTTACGGTCACCGACTTCAGGCAC | roseo_2_19 | CTCTCTGGCGGTCCCGGGATGTCAA | gamma_13_19 | GAGCTTCAAGAGAGGCCCTCTTTCT |
| flavo_6_20 | CCGGAGTTAAGCCCCTGCATTTCAC | roseo_2_20 | CTGGCGGTCCCGGGATGTCAAGGGT | gamma_13_20 | TCTTGGCTCCAAAAGGCACACTCTC |
| flavo_6_21 | AATGGCAGTGCCGGAGTTAAGCCCC | roseo_2_21 | ACCTGTCACCGGGTCACCGAAGTGA | gamma_13_21 | AGTGTCAGTACAGATCCAGGAGGCC |
| flavo_6_22 | TATCAATGGCAGTGCCGGAGTTAAG | roseo_2_22 | CCTGTCACCGGGTCACCGAAGTGAA | gamma_13_22 | GGCCCTCTTTCTCCCTTAGGAGGTA |
| flavo_6_23 | GTATCAATGGCAGTGCCGGAGTTAA | roseo_2_23 | CTGTCACCGGGTCACCGAAGTGAAA | gamma_13_23 | AGCTTCAAGAGAGGCCCTCTTTCTC |
| flavo_6_24 | CCCCTGCATTTCACCACTGACTTAT | roseo_2_24 | CGGGTCACCGAAGTGAAAACCAGAT | gamma_13_24 | AGCTGCGCCACTGAAAGACATTGTC |
| flavo_6_25 | TAAGCCCCTGCATTTCACCACTGAC | roseo_2_25 | AAAACCAGATCTCTCTGGCGGTCCC | gamma_13_25 | CGAGAGCATCAAGAGAGGCCCTCTT |
| flavo_7_1 | TCTTACAGTACCGTCACCAGACTAC | roseo_3_1 | GCCGCTACACCCGAAGGTGCCGCTC | gamma_14_1 | GGCGGTCAACTTACTACGTTAGCTG |
| flavo_7_2 | CTTACAGTACCGTCACCAGACTACA | roseo_3_2 | CTACACCCGAAGGTGCCGCTCGACT | gamma_14_2 | CCAGGCGGTCAACTTACTACGTTAG |
| flavo_7_3 | CGTCACCAGACTACACGTAGTCCTT | roseo_3_3 | GCTACACCCGAAGGTGCCGCTCGAC | gamma_14_3 | GCGGTCAACTTACTACGTTAGCTGC |
| flavo_7_4 | GTACCGTCACCAGACTACACGTAGT | roseo_3_4 | CCGCTACACCCGAAGGTGCCGCTCG | gamma_14_4 | CAGGCGGTCAACTTACTACGTTAGC |
| flavo_7_5 | CCGTCACCAGACTACACGTAGTCCT | roseo_3_5 | CGCTACACCCGAAGGTGCCGCTCGA | gamma_14_5 | CCCAGGCGGTCAACTTACTACGTTA |
| flavo_7_6 | TACCGTCACCAGACTACACGTAGTC | roseo_3_6 | CGCCGCTACACCCGAAGGTGCCGCT | gamma_14_6 | CCGAGGGCACTGCTTCATTACAAAG |
| flavo_7_7 | ACCGTCACCAGACTACACGTAGTCC | roseo_3_7 | CCGCCGCTACACCCGAAGGTGCCGC | gamma_14_7 | CGAGGGCACTGCTTCATTACAAAGC |
| flavo_7_8 | TTACAGTACCGTCACCAGACTACAC | roseo_3_8 | TACACCCGAAGGTGCCGCTCGACTT | gamma_14_8 | TCCCGAGGGCACTGCTTCATTACAA |
| flavo_7_9 | GTCACCAGACTACACGTAGTCCTTA | roseo_3_9 | TCCGCCGCTACACCCGAAGGTGCCG | gamma_14_9 | CCCGAGGGCACTGCTTCATTACAAA |
| flavo_7_10 | TACAGTACCGTCACCAGACTACACG | roseo_3_10 | ACACCCGAAGGTGCCGCTCGACTTG | gamma_14_10 | CCCCAGGCGGTCAACTTACTACGTT |
| flavo_7_11 | ACAGTACCGTCACCAGACTACACGT | roseo_3_11 | GTCCGCCGCTACACCCGAAGGTGCC | gamma_14_11 | TCCCCAGGCGGTCAACTTACTACGT |
| flavo_7_12 | AACTTTCACCCCTGACTTAACAGCC | roseo_3_12 | ACCCGAAGGTGCCGCTCGACTTGCA | gamma_14_12 | CTCCCGAGGGCACTGCTTCATTACA |
| flavo_7_13 | CAGTACCGTCACCAGACTACACGTA | roseo_3_13 | CACCCGAAGGTGCCGCTCGACTTGC | gamma_14_13 | CTCCCCAGGCGGTCAACTTACTACG |
| flavo_7_14 | CCGGTCGTCAGCAAGAGCAAGCTCC | roseo_3_14 | CGTCCGCCGCTACACCCGAAGGTGC | gamma_14_14 | GCTCCCGAGGGCACTGCTTCATTAC |
| flavo_7_15 | ACTTTCACCCCTGACTTAACAGCCC | roseo_3_15 | CACCTGGTCTCTTACGAGAAAACCG | gamma_14_15 | TCTTGGCTCCCGAGGGCACTGCTTC |
| flavo_7_16 | CCCTGACTTAACAGCCCGCCTACGG | roseo_3_16 | CCAGGAGTTTTGGAGGCCGTTCCAG | gamma_14_16 | GGCTCCCGAGGGCACTGCTTCATTA |
| flavo_7_17 | TCGCTTGGCCGCTCAGATCGAAATC | roseo_3_17 | ACCTGGTCTCTTACGAGAAAACCGG | gamma_14_17 | TATCTTGGCTCCCGAGGGCACTGCT |
| flavo_7_18 | CGCTTGGCCGCTCAGATCGAAATCC | roseo_3_18 | CCGGATCTCTCCGGCGGTCCAGGGA | gamma_14_18 | ACTCCCCAGGCGGTCAACTTACTAC |
| flavo_7_19 | TTCGCTTGGCCGCTCAGATCGAAAT | roseo_3_19 | CCCGAAGGTGCCGCTCGACTTGCAT | gamma_14_19 | ATCTTGGCTCCCGAGGGCACTGCTT |
| flavo_7_20 | TTTCGCTTGGCCGCTCAGATCGAAA | roseo_3_20 | ACCAGGAGTTTTGGAGGCCGTTCCA | gamma_14_20 | TACTACGTTAGCTGCGCCACTGAGA |
| flavo_7_21 | GCTTGGCCGCTCAGATCGAAATCCA | roseo_3_21 | CAGGAGTTTTGGAGGCCGTTCCAGG | gamma_14_21 | GTATCTTGGCTCCCGAGGGCACTGC |
| flavo_7_22 | CTTGGCCGCTCAGATCGAAATCCAA | roseo_3_22 | CCGAAGGTGCCGCTCGACTTGCATG | gamma_14_22 | CTTGGCTCCCGAGGGCACTGCTTCA |
| flavo_7_23 | TTGGCCGCTCAGATCGAAATCCAAA | roseo_3_23 | CCGTCCGCCGCTACACCCGAAGGTG | gamma_14_23 | TGGCTCCCGAGGGCACTGCTTCATT |
| flavo_7_24 | GGCTATCCCTTAGTGTAAGGCAGAT | roseo_3_24 | AAACCGGATCTCTCCGGCGGTCCAG | gamma_14_24 | ACTACGTTAGCTGCGCCACTGAGAA |
| flavo_7_25 | GGGCTATCCCTTAGTGTAAGGCAGA | roseo_3_25 | CCTGGTCTCTTACGAGAAAACCGGA | gamma_14_25 | TTGGCTCCCGAGGGCACTGCTTCAT |
| flavo_8_1 | GCCGAAATACGGTACTACGGGGCAT | roseo_4_1 | CGTACCATCTCTGGTAGTAGCACAG | gamma_15_1 | TCCGTAGAAGTCCGGGCCGTGTCTC |
| flavo_8_2 | GATGCCGAAATACGGTACTACGGGG | roseo_4_2 | CCATCTCTGGTAGTAGCACAGGATG | gamma_15_2 | CCGTAGAAGTCCGGGCCGTGTCTCA |
| flavo_8_3 | ATGCCGAAATACGGTACTACGGGGC | roseo_4_3 | GTACCATCTCTGGTAGTAGCACAGG | gamma_15_3 | CGTAGAAGTCCGGGCCGTGTCTCAG |
| flavo_8_4 | TGCCGAAATACGGTACTACGGGGCA | roseo_4_4 | CTGGTAGTAGCACAGGATGTCAAGG | gamma_15_4 | GTAGAAGTCCGGGCCGTGTCTCAGT |
| flavo_8_5 | ACCGTATAACGATGCCGAAATACGG | roseo_4_5 | TGGTAGTAGCACAGGATGTCAAGGG | gamma_15_5 | TTCCGTAGAAGTCCGGGCCGTGTCT |
| flavo_8_6 | CCGTATAACGATGCCGAAATACGGT | roseo_4_6 | GAAGGGAACGTACCATCTCTGGTAG | gamma_15_6 | CTTCCGTAGAAGTCCGGGCCGTGTC |
| flavo_8_7 | CGATGCCGAAATACGGTACTACGGG | roseo_4_7 | CCTTAGAGAAGGGCATATTCCCACG | gamma_15_7 | TAGAAGTCCGGGCCGTGTCTCAGTC |
| flavo_8_8 | CCGAAATACGGTACTACGGGGCATT | roseo_4_8 | GGTAGTAGCACAGGATGTCAAGGGT | gamma_15_8 | ACTGCTGCCTTCCGTAGAAGTCCGG |
| flavo_8_9 | ACGATGCCGAAATACGGTACTACGG | roseo_4_9 | GGGAACGTACCATCTCTGGTAGTAG | gamma_15_9 | CATGCAGTCGAGTTCCAGACTGCAA |
| flavo_8_10 | AACGATGCCGAAATACGGTACTACG | roseo_4_10 | GGAACGTACCATCTCTGGTAGTAGC | gamma_15_10 | CCTCGAGCTATCCCCCTCCATTGGG |
| flavo_8_11 | CGAAGGAAAAGTCATCTCTGACCCT | roseo_4_11 | CGAAGGGAACGTACCATCTCTGGTA | gamma_15_11 | AGAAGTCCGGGCCGTGTCTCAGTCC |
| flavo_8_12 | CGAAATACGGTACTACGGGGCATTA | roseo_4_12 | CCGAAGGGAACGTACCATCTCTGGT | gamma_15_12 | TCCTCGAGCTATCCCCCTCCATTGG |
| flavo_8_13 | CCGAAGGAAAAGTCATCTCTGACCC | roseo_4_13 | CGTCCCCGAAGGGAACGTACCATCT | gamma_15_13 | CTCGAGCTATCCCCCTCCATTGGGT |
| flavo_8_14 | GTCATCTCTGACCCTGTCAATATGC | roseo_4_14 | CCCCGAAGGGAACGTACCATCTCTG | gamma_15_14 | TCATGCAGTCGAGTTCCAGACTGCA |
| flavo_8_15 | CCCGAAGGAAAAGTCATCTCTGACC | roseo_4_15 | GTCCCCGAAGGGAACGTACCATCTC | gamma_15_15 | CCTTCCGTAGAAGTCCGGGCCGTGT |
| flavo_8_16 | TACAAGGCAGGTTCCATACGCGGTG | roseo_4_16 | GCGTCCCCGAAGGGAACGTACCATC | gamma_15_16 | GCGCCACTGGATAAATCCAACGGCT |
| flavo_8_17 | GGCTTTAACCGTATAACGATGCCGA | roseo_4_17 | ACTGCGTCCCCGAAGGGAACGTACC | gamma_15_17 | TGCGCCACTGGATAAATCCAACGGC |
| flavo_8_18 | CTGGGCTATTCCCCTGTACAAGGCA | roseo_4_18 | CTGCGTCCCCGAAGGGAACGTACCA | gamma_15_18 | TTCCTCGAGCTATCCCCCTCCATTG |
| flavo_8_19 | GAAGGAAAAGTCATCTCTGACCCTG | roseo_4_19 | CCCGAAGGGAACGTACCATCTCTGG | gamma_15_19 | GTTCCAGACTGCAATTCGGACTACG |
| flavo_8_20 | GCCCGAAGGAAAAGTCATCTCTGAC | roseo_4_20 | TGCGTCCCCGAAGGGAACGTACCAT | gamma_15_20 | CCAGCTCGCGCTTTGGCAACCGTTT |
| flavo_8_21 | GTACAAGGCAGGTTCCATACGCGGT | roseo_4_21 | CTTAGAGAAGGGCATATTCCCACGC | gamma_15_21 | TCGAGCTATCCCCCTCCATTGGGTA |
| flavo_8_22 | TGTACAAGGCAGGTTCCATACGCGG | roseo_4_22 | GAAGGGCGCGCTCGACTTGCATGTA | gamma_15_22 | GCTGCGCCACTGGATAAATCCAACG |
| flavo_8_23 | CCTGGGCTATTCCCCTGTACAAGGC | roseo_4_23 | CACTGCGTCCCCGAAGGGAACGTAC | gamma_15_23 | CGCCACTGGATAAATCCAACGGCTA |
| flavo_8_24 | ACAAGGCAGGTTCCATACGCGGTGC | roseo_4_24 | TCACTGCGTCCCCGAAGGGAACGTA | gamma_15_24 | CTGCGCCACTGGATAAATCCAACGG |
| flavo_8_25 | GGCAGGTTCCATACGCGGTGCGCAC | roseo_4_25 | TCCCCGAAGGGAACGTACCATCTCT | gamma_15_25 | TTTCCTCGAGCTATCCCCCTCCATT |
| flavo_9_1 | ATTCCGCCTACTTCAATACAACTCA | roseo_5_1 | GTCACTATGTCCCGAAGGAAAGCCT | gamma_16_1 | TTTAAGGGTTTGGCTCCAGCTCGCG |
| flavo_9_2 | TTCCGCCTACTTCAATACAACTCAA | roseo_5_2 | CCGAAGGAAAGCCTGATCTCTCAGG | gamma_16_2 | TTTTAAGGGTTTGGCTCCAGCTCGC |
| flavo_9_3 | TATTCCGCCTACTTCAATACAACTC | roseo_5_3 | TGTCACTATGTCCCGAAGGAAAGCC | gamma_16_3 | TTAAGGGTTTGGCTCCAGCTCGCGC |
| flavo_9_4 | TCCGCCTACTTCAATACAACTCAAG | roseo_5_4 | TCCCGAAGGAAAGCCTGATCTCTCA | gamma_16_4 | GTTTTAAGGGTTTGGCTCCAGCTCG |
| flavo_9_5 | CATATTCCGCCTACTTCAATACAAC | roseo_5_5 | TCACTATGTCCCGAAGGAAAGCCTG | gamma_16_5 | CACGCGGTATACCTGGATCAGGGTT |
| flavo_9_6 | CCGCCTACTTCAATACAACTCAAGA | roseo_5_6 | CCCGAAGGAAAGCCTGATCTCTCAG | gamma_16_6 | ACACGCGGTATACCTGGATCAGGGT |
| flavo_9_7 | CGCCTACTTCAATACAACTCAAGAT | roseo_5_7 | CTGTCACTATGTCCCGAAGGAAAGC | gamma_16_7 | CTTCCTCCGGGTTTCACCCGGCAGT |
| flavo_9_8 | GAACTCAAGGTCCCGAACAGCTAGT | roseo_5_8 | GTCCCGAAGGAAAGCCTGATCTCTC | gamma_16_8 | TCCTCCGGGTTTCACCCGGCAGTCT |
| flavo_9_9 | TCAGAACTCAAGGTCCCGAACAGCT | roseo_5_9 | GCCTGATCTCTCAGGTTGTCATAGG | gamma_16_9 | CTTCACACACGCGGTATACCTGGAT |
| flavo_9_10 | ACTCAAGGTCCCGAACAGCTAGTAT | roseo_5_10 | TGACTGACTAATCCGCCTACGTACG | gamma_16_10 | CACACGCGGTATACCTGGATCAGGG |
| flavo_9_11 | GATGCCTATCAATAATACCATGAGG | roseo_5_11 | CTGACTGACTAATCCGCCTACGTAC | gamma_16_11 | ACACACGCGGTATACCTGGATCAGG |
| flavo_9_12 | AGAACTCAAGGTCCCGAACAGCTAG | roseo_5_12 | CGAAGGAAAGCCTGATCTCTCAGGT | gamma_16_12 | CACACACGCGGTATACCTGGATCAG |
| flavo_9_13 | CTCAAGGTCCCGAACAGCTAGTATC | roseo_5_13 | CACTATGTCCCGAAGGAAAGCCTGA | gamma_16_13 | CCTTCCTCCGGGTTTCACCCGGCAG |
| flavo_9_14 | AACTCAAGGTCCCGAACAGCTAGTA | roseo_5_14 | GCACCTGTCACTATGTCCCGAAGGA | gamma_16_14 | TTCCTCCGGGTTTCACCCGGCAGTC |
| flavo_9_15 | CAGAACTCAAGGTCCCGAACAGCTA | roseo_5_15 | CCTGTCACTATGTCCCGAAGGAAAG | gamma_16_15 | CCTCCGGGTTTCACCCGGCAGTCTC |
| flavo_9_16 | CTCAGAACTCAAGGTCCCGAACAGC | roseo_5_16 | CTATGTCCCGAAGGAAAGCCTGATC | gamma_16_16 | TTCACACACGCGGTATACCTGGATC |
| flavo_9_17 | TCAAGGTCCCGAACAGCTAGTATCC | roseo_5_17 | ATGTCCCGAAGGAAAGCCTGATCTC | gamma_16_17 | CGCCTTCCTCCGGGTTTCACCCGGC |
| flavo_9_18 | GCTCAGAACTCAAGGTCCCGAACAG | roseo_5_18 | AGCACCTGTCACTATGTCCCGAAGG | gamma_16_18 | CTCCGGGTTTCACCCGGCAGTCTCC |
| flavo_9_19 | CTACATATTCCGCCTACTTCAATAC | roseo_5_19 | CAGCACCTGTCACTATGTCCCGAAG | gamma_16_19 | GCGGTATACCTGGATCAGGGTTGCC |
| flavo_9_20 | GCCTACTTCAATACAACTCAAGATG | roseo_5_20 | CCTCCGAAGAGGTTAGCGCACGGCC | gamma_16_20 | CGGTATACCTGGATCAGGGTTGCCC |
| flavo_9_21 | TACACGTAAGGCTTATTCTTCCTGT | roseo_5_21 | TCCGCTGCCTCCTCCGAAGAGGTTA | gamma_16_21 | GGTATACCTGGATCAGGGTTGCCCC |
| flavo_9_22 | CACGTAAGGCTTATTCTTCCTGTAT | roseo_5_22 | CCGCTGCCTCCTCCGAAGAGGTTAG | gamma_16_22 | TCTTCACACACGCGGTATACCTGGA |
| flavo_9_23 | ACACGTAAGGCTTATTCTTCCTGTA | roseo_5_23 | TGTCCCGAAGGAAAGCCTGATCTCT | gamma_16_23 | TCACACACGCGGTATACCTGGATCA |
| flavo_9_24 | CTTAGCCGCTCAGAACTCAAGGTCC | roseo_5_24 | CACCTGTCACTATGTCCCGAAGGAA | gamma_16_24 | GCCTTCCTCCGGGTTTCACCCGGCA |
| flavo_9_25 | CGCTCAGAACTCAAGGTCCCGAACA | roseo_5_25 | GCAGCACCTGTCACTATGTCCCGAA | gamma_16_25 | CGCGGTATACCTGGATCAGGGTTGC |
| flavo_10_1 | CGCTTAGCCACTCATCTAACCAATG | roseo_6_1 | CGATAAAACCTAGTCTCCTAGGCGG | gamma_17_1 | GGCTCCTCCAATAGTGACCGGTCCG |
| flavo_10_2 | CTTTCGCTTAGCCACTCATCTAACC | roseo_6_2 | CCGAGGCTATTCCGAAGCAAAAGGT | gamma_17_2 | AGGCTCCTCCAATAGTGACCGGTCC |
| flavo_10_3 | ACACGTCGGAGTGTTTCTTCCTGTA | roseo_6_3 | CCCGAGGCTATTCCGAAGCAAAAGG | gamma_17_3 | CAGGCTCCTCCAATAGTGACCGGTC |
| flavo_10_4 | CCCGTGCGCCACTCGTCATCTGGTG | roseo_6_4 | AAAACCTAGTCTCCTAGGCGGTCAG | gamma_17_4 | CATGTATTAGGCCTGCCGCCAACGT |
| flavo_10_5 | ACCCGTGCGCCACTCGTCATCTGGT | roseo_6_5 | AAACCTAGTCTCCTAGGCGGTCAGA | gamma_17_5 | GCTCCTCCAATAGTGACCGGTCCGA |
| flavo_10_6 | CACCCGTGCGCCACTCGTCATCTGG | roseo_6_6 | TCCCGAGGCTATTCCGAAGCAAAAG | gamma_17_6 | GCAGGCTCCTCCAATAGTGACCGGT |
| flavo_10_7 | TACAACCCGTAGGGCTTTCATCCTG | roseo_6_7 | CTAGTCTCCTAGGCGGTCAGAGGAT | gamma_17_7 | CGCCTGAGAGCAAGCTCCCATCGTT |
| flavo_10_8 | ACAACCCGTAGGGCTTTCATCCTGC | roseo_6_8 | AACCTAGTCTCCTAGGCGGTCAGAG | gamma_17_8 | ACGCCTGAGAGCAAGCTCCCATCGT |
| flavo_10_9 | AACCCGTAGGGCTTTCATCCTGCAC | roseo_6_9 | CCTAGTCTCCTAGGCGGTCAGAGGA | gamma_17_9 | GCCTGAGAGCAAGCTCCCATCGTTT |
| flavo_10_10 | CAGTTTACAACCCGTAGGGCTTTCA | roseo_6_10 | TAGTCTCCTAGGCGGTCAGAGGATG | gamma_17_10 | GACGCCTGAGAGCAAGCTCCCATCG |
| flavo_10_11 | CAACCCGTAGGGCTTTCATCCTGCA | roseo_6_11 | CCTCTCAAACCAGCTACTGATCGCA | gamma_17_11 | AATCCTACGCAGGCTCCTCCAATAG |
| flavo_10_12 | TTACAACCCGTAGGGCTTTCATCCT | roseo_6_12 | TCCTCTCAAACCAGCTACTGATCGC | gamma_17_12 | GCATGTATTAGGCCTGCCGCCAACG |
| flavo_10_13 | AGCAGTTTACAACCCGTAGGGCTTT | roseo_6_13 | CTCTCAAACCAGCTACTGATCGCAG | gamma_17_13 | CTAATCCTACGCAGGCTCCTCCAAT |
| flavo_10_14 | GCAGTTTACAACCCGTAGGGCTTTC | roseo_6_14 | CTCAAACCAGCTACTGATCGCAGAC | gamma_17_14 | GCTAATCCTACGCAGGCTCCTCCAA |
| flavo_10_15 | AAGCAGTTTACAACCCGTAGGGCTT | roseo_6_15 | CAGCTACTGATCGCAGACTTGGTAG | gamma_17_15 | CGACGCCTGAGAGCAAGCTCCCATC |
| flavo_10_16 | CACGTCGGAGTGTTTCTTCCTGTAT | roseo_6_16 | CCAGCTACTGATCGCAGACTTGGTA | gamma_17_16 | CCTGAGAGCAAGCTCCCATCGTTTC |
| flavo_10_17 | TGCGCCACTCGTCATCTGGTGCAAG | roseo_6_17 | CCATGCAGCACCTGTCACTCTGTAT | gamma_17_17 | CTCCTCCAATAGTGACCGGTCCGAA |
| flavo_10_18 | CCGTGCGCCACTCGTCATCTGGTGC | roseo_6_18 | CATGCAGCACCTGTCACTCTGTATC | gamma_17_18 | ATCCTACGCAGGCTCCTCCAATAGT |
| flavo_10_19 | GCGCCACTCGTCATCTGGTGCAAGC | roseo_6_19 | AACCAGCTACTGATCGCAGACTTGG | gamma_17_19 | CGCAGGCTCCTCCAATAGTGACCGG |
| flavo_10_20 | CGTGCGCCACTCGTCATCTGGTGCA | roseo_6_20 | ACCAGCTACTGATCGCAGACTTGGT | gamma_17_20 | AGCTAATCCTACGCAGGCTCCTCCA |
| flavo_10_21 | GTGCGCCACTCGTCATCTGGTGCAA | roseo_6_21 | GCCATGCAGCACCTGTCACTCTGTA | gamma_17_21 | TCGACGCCTGAGAGCAAGCTCCCAT |
| flavo_10_22 | GTTTACAACCCGTAGGGCTTTCATC | roseo_6_22 | AGTTTCCCGAGGCTATTCCGAAGCA | gamma_17_22 | CTGAGAGCAAGCTCCCATCGTTTCC |
| flavo_10_23 | TTTACAACCCGTAGGGCTTTCATCC | roseo_6_23 | GTTTCCCGAGGCTATTCCGAAGCAA | gamma_17_23 | TGTATTAGGCCTGCCGCCAACGTTC |
| flavo_10_24 | GCACCCGTGCGCCACTCGTCATCTG | roseo_6_24 | GGCGGTCAGAGGATGTCAAGGGTTG | gamma_17_24 | TGCATGTATTAGGCCTGCCGCCAAC |
| flavo_10_25 | GCGAAGTGGCTGCTCTCTGTACCGG | roseo_6_25 | AGGCGGTCAGAGGATGTCAAGGGTT | gamma_17_25 | CGCCACCGGTATTCCTCAGAATATC |
| flavo_11_1 | GTACAAGTACTTTATGCTGCCCCTC | alpha_4_1 | CGACAGGCATGCCTGCCAACAACTA | gamma_19_1 | GAGGTTGCGACCCTTTGTCCTTCCC |
| flavo_11_2 | CCGCCGGAGCTTTTCTTAAAAACTC | alpha_4_2 | CCGACAGGCATGCCTGCCAACAACT | gamma_19_2 | GCGAGGTTGCGACCCTTTGTCCTTC |
| flavo_11_3 | CGGTCGCCATCAAAGTACAAGTACT | alpha_4_3 | ACCGACAGGCATGCCTGCCAACAAC | gamma_19_3 | CGAAACCTTTCAAGAAGAGGGCTCC |
| flavo_11_4 | CCGGTCGCCATCAAAGTACAAGTAC | alpha_4_4 | GACAGGCATGCCTGCCAACAACTAG | gamma_19_4 | AAAGTGGTGAGCGCCCAGATAAGCT |
| flavo_11_5 | CGTCCCTCAGCGTCAGTTAATTGTT | alpha_4_5 | CCGTCTGCCACTATATCGTTCGACT | gamma_19_5 | TGAGCGCCCAGATAAGCTACCCACT |
| flavo_11_6 | TACAAGTACTTTATGCTGCCCCTCG | alpha_4_6 | CACCGACAGGCATGCCTGCCAACAA | gamma_19_6 | CAAAGTGGTGAGCGCCCAGATAAGC |
| flavo_11_7 | CACGCGGCATCGCTGGATCAGAGTT | alpha_4_7 | CCCGTCTGCCACTATATCGTTCGAC | gamma_19_7 | GTGGTGAGCGCCCAGATAAGCTACC |
| flavo_11_8 | TCGTCCCTCAGCGTCAGTTAATTGT | alpha_4_8 | CAGGCATGCCTGCCAACAACTAGCT | gamma_19_8 | AGTGGTGAGCGCCCAGATAAGCTAC |
| flavo_11_9 | TCACGCGGCATCGCTGGATCAGAGT | alpha_4_9 | ACAGGCATGCCTGCCAACAACTAGC | gamma_19_9 | GTGAGCGCCCAGATAAGCTACCCAC |
| flavo_11_10 | TGCCAGTATCAAAGGCAGTTCTACC | alpha_4_10 | TCACCGACAGGCATGCCTGCCAACA | gamma_19_10 | GGTGAGCGCCCAGATAAGCTACCCA |
| flavo_11_11 | ACAAGTACTTTATGCTGCCCCTCGA | alpha_4_11 | GCATGCCTGCCAACAACTAGCTCTC | gamma_19_11 | TGGTGAGCGCCCAGATAAGCTACCC |
| flavo_11_12 | GTACATCGAACAGCTAGTGACCATC | alpha_4_12 | GGCATGCCTGCCAACAACTAGCTCT | gamma_19_12 | AAGTGGTGAGCGCCCAGATAAGCTA |
| flavo_11_13 | GCCAGTATCAAAGGCAGTTCTACCG | alpha_4_13 | CACCCGTCTGCCACTATATCGTTCG | gamma_19_13 | CGCCCAGATAAGCTACCCACTTCTT |
| flavo_11_14 | TTCGTCCCTCAGCGTCAGTTAATTG | alpha_4_14 | ACCCGTCTGCCACTATATCGTTCGA | gamma_19_14 | GCGCCCAGATAAGCTACCCACTTCT |
| flavo_11_15 | CAAGTACTTTATGCTGCCCCTCGAC | alpha_4_15 | GTCACCGACAGGCATGCCTGCCAAC | gamma_19_15 | GCGAAACCTTTCAAGAAGAGGGCTC |
| flavo_11_16 | CGCCGGTCGCCATCAAAGTACAAGT | alpha_4_16 | AGGCATGCCTGCCAACAACTAGCTC | gamma_19_16 | AGCGCCCAGATAAGCTACCCACTTC |
| flavo_11_17 | TCGCCGGTCGCCATCAAAGTACAAG | alpha_4_17 | CTCACCCGTCTGCCACTATATCGTT | gamma_19_17 | ACAAAGTGGTGAGCGCCCAGATAAG |
| flavo_11_18 | GCCGGTCGCCATCAAAGTACAAGTA | alpha_4_18 | TCACCCGTCTGCCACTATATCGTTC | gamma_19_18 | CACAAAGTGGTGAGCGCCCAGATAA |
| flavo_11_19 | TTCGCCGGTCGCCATCAAAGTACAA | alpha_4_19 | CATGCCTGCCAACAACTAGCTCTCA | gamma_19_19 | CGAGGTTGCGACCCTTTGTCCTTCC |
| flavo_11_20 | CGTTCGCCGGTCGCCATCAAAGTAC | alpha_4_20 | CCTGCCAACAACTAGCTCTCATCGT | gamma_19_20 | GAGCGCCCAGATAAGCTACCCACTT |
| flavo_11_21 | GTTCGCCGGTCGCCATCAAAGTACA | alpha_4_21 | CGTCACCGACAGGCATGCCTGCCAA | gamma_19_21 | CGCGAGGTTGCGACCCTTTGTCCTT |
| flavo_11_22 | TACCTATCGGAGCTTAGGTGAGCCG | alpha_4_22 | CTCGGTATTCCGCTAACCTCTCCTG | gamma_19_22 | GACGCCTAAGAGCAAGCTCTTATCG |
| flavo_11_23 | TATCGGAGCTTAGGTGAGCCGTTAC | alpha_4_23 | ACTCACCCGTCTGCCACTATATCGT | gamma_19_23 | TCACAAAGTGGTGAGCGCCCAGATA |
| flavo_11_24 | CCCTGACTTAACAAACAGCCTGCGG | alpha_4_24 | GCGTCACCGACAGGCATGCCTGCCA | gamma_19_24 | GCAGGCTCATCTGATAGCGAAACCT |
| flavo_11_25 | ACCGTTGAGCGGTAGGATTTCACCC | alpha_4_25 | TACTCACCCGTCTGCCACTATATCG | gamma_19_25 | CGACGCCTAAGAGCAAGCTCTTATC |
| flavo_12_1 | CGTCTTCCTGCACGCTGCATGGCTG | wolbach_1_1 | GCCAGGACTTCTTCTGTGAGTACCG | gamma_20_1 | CCACTAAGGGACAAATTCCCCCAAC |
| flavo_12_2 | CCGTCTTCCTGCACGCTGCATGGCT | wolbach_1_2 | AGCCAGGACTTCTTCTGTGAGTACC | gamma_20_2 | CGCCACTAAGGGACAAATTCCCCCA |
| flavo_12_3 | GTCTTCCTGCACGCTGCATGGCTGG | wolbach_1_3 | CCAGGACTTCTTCTGTGAGTACCGT | gamma_20_3 | GCCACTAAGGGACAAATTCCCCCAA |
| flavo_12_4 | CTTCCTGCACGCTGCATGGCTGGAT | wolbach_1_4 | CGGAGTTAGCCAGGACTTCTTCTGT | gamma_20_4 | CACTAAGGGACAAATTCCCCCAACG |
| flavo_12_5 | TTCCTGCACGCTGCATGGCTGGATC | wolbach_1_5 | CCGGCCGAACCGACCCTATCCCTTC | gamma_20_5 | ACTAAGGGACAAATTCCCCCAACGG |
| flavo_12_6 | GCCGTCTTCCTGCACGCTGCATGGC | wolbach_1_6 | ACGGAGTTAGCCAGGACTTCTTCTG | gamma_20_6 | CTAAGGGACAAATTCCCCCAACGGC |
| flavo_12_7 | TCTTCCTGCACGCTGCATGGCTGGA | wolbach_1_7 | GGAGTTAGCCAGGACTTCTTCTGTG | gamma_20_7 | GCGCCACTAAGGGACAAATTCCCCC |
| flavo_12_8 | CACGCTGCATGGCTGGATCAGAGTT | wolbach_1_8 | CAGGACTTCTTCTGTGAGTACCGTC | gamma_20_8 | GGTACCGTCAAGACGCGCAGTTATT |
| flavo_12_9 | GGCCGTCTTCCTGCACGCTGCATGG | wolbach_1_9 | GGCACGGAGTTAGCCAGGACTTCTT | gamma_20_9 | AGGTACCGTCAAGACGCGCAGTTAT |
| flavo_12_10 | TGCCCACCTTTTACCACCGGAGTTT | wolbach_1_10 | CACGGAGTTAGCCAGGACTTCTTCT | gamma_20_10 | TAGGTACCGTCAAGACGCGCAGTTA |
| flavo_12_11 | ATGCCCACCTTTTACCACCGGAGTT | wolbach_1_11 | TGGCACGGAGTTAGCCAGGACTTCT | gamma_20_11 | TGCGCCACTAAGGGACAAATTCCCC |
| flavo_12_12 | CACACGTGGACAGATTTCTTCCTGT | wolbach_1_12 | GCACGGAGTTAGCCAGGACTTCTTC | gamma_20_12 | TAAGGGACAAATTCCCCCAACGGCT |
| flavo_12_13 | GAAGACTCGCTCTTCCTCGCGGAGT | wolbach_1_13 | CGCCTCAGCGTCAGATTTGAACCAG | gamma_20_13 | CTGTAGGTACCGTCAAGACGCGCAG |
| flavo_12_14 | CATGCCCACCTTTTACCACCGGAGT | wolbach_1_14 | GCGCCTCAGCGTCAGATTTGAACCA | gamma_20_14 | GTAGGTACCGTCAAGACGCGCAGTT |
| flavo_12_15 | CCGGCTTTGAAGACTCGCTCTTCCT | wolbach_1_15 | CTGGCACGGAGTTAGCCAGGACTTC | gamma_20_15 | CTGCGCCACTAAGGGACAAATTCCC |
| flavo_12_16 | CCACACGTGGACAGATTTCTTCCTG | wolbach_1_16 | CTGCTGGCACGGAGTTAGCCAGGAC | gamma_20_16 | TGTAGGTACCGTCAAGACGCGCAGT |
| flavo_12_17 | TTTGAAGACTCGCTCTTCCTCGCGG | wolbach_1_17 | GCTGGCACGGAGTTAGCCAGGACTT | gamma_20_17 | TCTGTAGGTACCGTCAAGACGCGCA |
| flavo_12_18 | GGCTTTGAAGACTCGCTCTTCCTCG | wolbach_1_18 | TGCTGGCACGGAGTTAGCCAGGACT | gamma_20_18 | GCTGCGCCACTAAGGGACAAATTCC |
| flavo_12_19 | CTTTGAAGACTCGCTCTTCCTCGCG | wolbach_1_19 | CGCGCCTCAGCGTCAGATTTGAACC | gamma_20_19 | CTTCTGTAGGTACCGTCAAGACGCG |
| flavo_12_20 | TGAAGACTCGCTCTTCCTCGCGGAG | wolbach_1_20 | GCCTTCGCGCCTCAGCGTCAGATTT | gamma_20_20 | TCTTCTGTAGGTACCGTCAAGACGC |
| flavo_12_21 | GACCGGCTTTGAAGACTCGCTCTTC | wolbach_1_21 | GCCTCAGCGTCAGATTTGAACCAGA | gamma_20_21 | GGACAAATTCCCCCAACGGCTAGTT |
| flavo_12_22 | CGGCTTTGAAGACTCGCTCTTCCTC | wolbach_1_22 | TCGCGCCTCAGCGTCAGATTTGAAC | gamma_20_22 | GACAAATTCCCCCAACGGCTAGTTG |
| flavo_12_23 | GCTTTGAAGACTCGCTCTTCCTCGC | wolbach_1_23 | CATGCAACACCTGTGTGAAACCCGG | gamma_20_23 | AGCTGCGCCACTAAGGGACAAATTC |
| flavo_12_24 | ACCGGCTTTGAAGACTCGCTCTTCC | wolbach_1_24 | GACTTTGCAGCCCATTGTAGCCACC | gamma_20_24 | CGTTACGCACCCGTCCGCCACTCGA |
| flavo_12_25 | TCGTACAGTACCGTCAACTACCCAC | wolbach_1_25 | CGACTTTGCAGCCCATTGTAGCCAC | gamma_20_25 | TCGCGTTAGCTGCGCCACTAAGGGA |
| flavo_13_1 | CGCCGGTCGTCAGCATAGCAAGCTA | rickett_1_1 | TCTCTGCGATCCGCGACCACCATGT | gamma_21_1 | TCGTCAGCGCAGAGCAAGCTCCGCC |
| flavo_13_2 | AGGTCGCTCCTCACGGTAACGAACT | rickett_1_2 | ATCTCTGCGATCCGCGACCACCATG | gamma_21_2 | CTCGTCAGCGCAGAGCAAGCTCCGC |
| flavo_13_3 | GGTCGCTCCTCACGGTAACGAACTT | rickett_1_3 | GTCAGTTGTAGCCCAGATGACCGCC | gamma_21_3 | ACTCGTCAGCGCAGAGCAAGCTCCG |
| flavo_13_4 | TAGGTCGCTCCTCACGGTAACGAAC | rickett_1_4 | CAGTTGTAGCCCAGATGACCGCCTT | gamma_21_4 | AGCAAGCTCCGCCTGTTACCGTTCG |
| flavo_13_5 | AGGACGCATAGTCATCTTGTACCCA | rickett_1_5 | TCAGTTGTAGCCCAGATGACCGCCT | gamma_21_5 | GTCAGCGCAGAGCAAGCTCCGCCTG |
| flavo_13_6 | CCTCACGGTAACGAACTTCAGGCAC | rickett_1_6 | CGTCAGTTGTAGCCCAGATGACCGC | gamma_21_6 | GAGCAAGCTCCGCCTGTTACCGTTC |
| flavo_13_7 | TCGCCCAGTGGCTGCTCATTGTCCA | rickett_1_7 | GTTGTAGCCCAGATGACCGCCTTCG | gamma_21_7 | CAAGCTCCGCCTGTTACCGTTCGAC |
| flavo_13_8 | CGTTCGCCGGTCGTCAGCATAGCAA | rickett_1_8 | AGTTGTAGCCCAGATGACCGCCTTC | gamma_21_8 | GCTCCGCCTGTTACCGTTCGACTTG |
| flavo_13_9 | GTCGCTCCTCACGGTAACGAACTTC | rickett_1_9 | CATCTCTGCGATCCGCGACCACCAT | gamma_21_9 | CTGGGCTTTCACATCCGACTGACCG |
| flavo_13_10 | GTCGCCCAGTGGCTGCTCATTGTCC | rickett_1_10 | GCGTCAGTTGTAGCCCAGATGACCG | gamma_21_10 | CTTTTGCAAGCCACTCCCATGGTGT |
| flavo_13_11 | TAGGACGCATAGTCATCTTGTACCC | rickett_1_11 | AGCATCTCTGCGATCCGCGACCACC | gamma_21_11 | TCTTTTGCAAGCCACTCCCATGGTG |
| flavo_13_12 | ACCAGTATCAAAGGCAGTTCCATCG | rickett_1_12 | GCATCTCTGCGATCCGCGACCACCA | gamma_21_12 | CTTCTTTTGCAAGCCACTCCCATGG |
| flavo_13_13 | TCCTCACGGTAACGAACTTCAGGCA | rickett_1_13 | TTGTAGCCCAGATGACCGCCTTCGC | gamma_21_13 | TTTTGCAAGCCACTCCCATGGTGTG |
| flavo_13_14 | CTAGGTCGCTCCTCACGGTAACGAA | rickett_1_14 | AGCGTCAGTTGTAGCCCAGATGACC | gamma_21_14 | TTTGCAAGCCACTCCCATGGTGTGA |
| flavo_13_15 | CTCCTCACGGTAACGAACTTCAGGC | rickett_1_15 | CCACTAACTAATTGGAGCAAGCCCC | gamma_21_15 | CCTCAGCGTCAGTATTGCTCCAGAA |
| flavo_13_16 | CCGTTCGCCGGTCGTCAGCATAGCA | rickett_1_16 | GCCACTAACTAATTGGAGCAAGCCC | gamma_21_16 | GGGCTTTCACATCCGACTGACCGTG |
| flavo_13_17 | GTTCGCCGGTCGTCAGCATAGCAAG | rickett_1_17 | CAAGCCCCAATTAGTCCGTTCGACT | gamma_21_17 | CTTTCACATCCGACTGACCGTGCCG |
| flavo_13_18 | CTCACGGTAACGAACTTCAGGCACT | rickett_1_18 | CCGTCTTGCTTCCCTCTGTAAACAC | gamma_21_18 | GGCTTTCACATCCGACTGACCGTGC |
| flavo_13_19 | TCGCTCCTCACGGTAACGAACTTCA | rickett_1_19 | CCGTCTGCCACTAACTAATTGGAGC | gamma_21_19 | CACTCGTCAGCGCAGAGCAAGCTCC |
| flavo_13_20 | GGTCGCCCAGTGGCTGCTCATTGTC | rickett_1_20 | CTCTGCGATCCGCGACCACCATGTC | gamma_21_20 | GCTTTCACATCCGACTGACCGTGCC |
| flavo_13_21 | CGGCATAGCTGGTTCAGAGTTGCCT | rickett_1_21 | GCAAGCCCCAATTAGTCCGTTCGAC | gamma_21_21 | TCAGCGCAGAGCAAGCTCCGCCTGT |
| flavo_13_22 | GGCATAGCTGGTTCAGAGTTGCCTC | rickett_1_22 | AGCAAGCCCCAATTAGTCCGTTCGA | gamma_21_22 | CGTCAGCGCAGAGCAAGCTCCGCCT |
| flavo_13_23 | CGCGGCATAGCTGGTTCAGAGTTGC | rickett_1_23 | TGTAGCCCAGATGACCGCCTTCGCC | gamma_21_23 | AGAGCAAGCTCCGCCTGTTACCGTT |
| flavo_13_24 | GCGGCATAGCTGGTTCAGAGTTGCC | rickett_1_24 | GAGCAAGCCCCAATTAGTCCGTTCG | gamma_21_24 | AGCTCCGCCTGTTACCGTTCGACTT |
| flavo_13_25 | GCATAGCTGGTTCAGAGTTGCCTCC | rickett_1_25 | GAAGAAAAGCATCTCTGCGATCCGC | gamma_21_25 | CAGAGCAAGCTCCGCCTGTTACCGT |
| flavo_14_1 | GTGCAAGCACTCCTGTTACCCCTCG | alpha_5_1 | ACCAAAGCCCTGTGGGCCCTAGCAG | verru_1_1 | CCCCGAGATTTCACACCTCACACAT |
| flavo_14_2 | AGTGCAAGCACTCCTGTTACCCCTC | alpha_5_2 | CACCAAAGCCCTGTGGGCCCTAGCA | verru_1_2 | CCCGAGATTTCACACCTCACACATC |
| flavo_14_3 | GCAAGCACTCCTGTTACCCCTCGAC | alpha_5_3 | CCAAAGCCCTGTGGGCCCTAGCAGC | verru_1_3 | TCACACCTCACACATCTATCCGCCT |
| flavo_14_4 | TGCAAGCACTCCTGTTACCCCTCGA | alpha_5_4 | ACCCTATGGTAGATCCCCACGCGTT | verru_1_4 | CACCTCACACATCTATCCGCCTACG |
| flavo_14_5 | CAAGCACTCCTGTTACCCCTCGACT | alpha_5_5 | CACCCTATGGTAGATCCCCACGCGT | verru_1_5 | TTCACACCTCACACATCTATCCGCC |
| flavo_14_6 | AAGCACTCCTGTTACCCCTCGACTT | alpha_5_6 | GCACCCTATGGTAGATCCCCACGCG | verru_1_6 | ACACCTCACACATCTATCCGCCTAC |
| flavo_14_7 | AGCACTCCTGTTACCCCTCGACTTG | alpha_5_7 | CCGCACCCTATGGTAGATCCCCACG | verru_1_7 | CACACCTCACACATCTATCCGCCTA |
| flavo_14_8 | GCACTCCTGTTACCCCTCGACTTGC | alpha_5_8 | CGCACCCTATGGTAGATCCCCACGC | verru_1_8 | GCCCCGAGATTTCACACCTCACACA |
| flavo_14_9 | TGCTACACGTAGCAGTGTTTCTTCC | alpha_5_9 | TATTCCGCACCCTATGGTAGATCCC | verru_1_9 | ACCTCACACATCTATCCGCCTACGC |
| flavo_14_10 | CCCGTGCGCCGGTCGTCAGCGAGTG | alpha_5_10 | ATTCCGCACCCTATGGTAGATCCCC | verru_1_10 | AGCCCCGAGATTTCACACCTCACAC |
| flavo_14_11 | TCGTCAGCGAGTGCAAGCACTCCTG | alpha_5_11 | TCCGCACCCTATGGTAGATCCCCAC | verru_1_11 | CTCCCGAAGGATAGCTCACGTACTT |
| flavo_14_12 | TGCGCCGGTCGTCAGCGAGTGCAAG | alpha_5_12 | CGCACCAGCTTCGGGTTGATCCAAC | verru_1_12 | CTGCCTCCCGAAGGATAGCTCACGT |
| flavo_14_13 | CGGTCGTCAGCGAGTGCAAGCACTC | alpha_5_13 | TTCCGCACCCTATGGTAGATCCCCA | verru_1_13 | GGCTATGAACCTCCTTGTTGCTCCT |
| flavo_14_14 | CCGTGCGCCGGTCGTCAGCGAGTGC | alpha_5_14 | CCACCAAAGCCCTGTGGGCCCTAGC | verru_1_14 | CCTCCCGAAGGATAGCTCACGTACT |
| flavo_14_15 | GCGCCGGTCGTCAGCGAGTGCAAGC | alpha_5_15 | CCCTATGGTAGATCCCCACGCGTTA | verru_1_15 | CCCGAAGGATAGCTCACGTACTTCG |
| flavo_14_16 | GGTCGTCAGCGAGTGCAAGCACTCC | alpha_5_16 | CCTATGGTAGATCCCCACGCGTTAC | verru_1_16 | TCCCGAAGGATAGCTCACGTACTTC |
| flavo_14_17 | GCCGGTCGTCAGCGAGTGCAAGCAC | alpha_5_17 | GCGCACCAGCTTCGGGTTGATCCAA | verru_1_17 | GAGGCTATGAACCTCCTTGTTGCTC |
| flavo_14_18 | GTCAGCGAGTGCAAGCACTCCTGTT | alpha_5_18 | GCACCAGCTTCGGGTTGATCCAACT | verru_1_18 | GACGCTGCCTCCCGAAGGATAGCTC |
| flavo_14_19 | CCGGTCGTCAGCGAGTGCAAGCACT | alpha_5_19 | AGCGCACCAGCTTCGGGTTGATCCA | verru_1_19 | AGGCTATGAACCTCCTTGTTGCTCC |
| flavo_14_20 | TCAGCGAGTGCAAGCACTCCTGTTA | alpha_5_20 | CTATGGTAGATCCCCACGCGTTACG | verru_1_20 | GCCTCCCGAAGGATAGCTCACGTAC |
| flavo_14_21 | CGTGCGCCGGTCGTCAGCGAGTGCA | alpha_5_21 | GCCACCAAAGCCCTGTGGGCCCTAG | verru_1_21 | CGCTGCCTCCCGAAGGATAGCTCAC |
| flavo_14_22 | CGCCGGTCGTCAGCGAGTGCAAGCA | alpha_5_22 | CACCAGCTTCGGGTTGATCCAACTC | verru_1_22 | TGCCTCCCGAAGGATAGCTCACGTA |
| flavo_14_23 | GTGCGCCGGTCGTCAGCGAGTGCAA | alpha_5_23 | TAGCGCACCAGCTTCGGGTTGATCC | verru_1_23 | ACGCTGCCTCCCGAAGGATAGCTCA |
| flavo_14_24 | CGTCAGCGAGTGCAAGCACTCCTGT | alpha_5_24 | CAAAGCCCTGTGGGCCCTAGCAGCT | verru_1_24 | GCTGCCTCCCGAAGGATAGCTCACG |
| flavo_14_25 | GTCGTCAGCGAGTGCAAGCACTCCT | alpha_5_25 | CGCCACCAAAGCCCTGTGGGCCCTA | verru_1_25 | AGGACGCTGCCTCCCGAAGGATAGC |
| flavo_15_1 | GGCGTACTCCCCAGGTGCATCACTT | alpha_6_1 | GCGCCACTAACCCCGAAGCTTCGTT | verru_2_1 | CGTCGCATGTTCACACTTTCGTGCA |
| flavo_15_2 | CTCCCCAGGTGCATCACTTAATACT | alpha_6_2 | CTTCTTGCGAGTAGCTGCCCACTGT | verru_2_2 | CTACCCTAACTTTCGTCCATGAGCG |
| flavo_15_3 | GCGTACTCCCCAGGTGCATCACTTA | alpha_6_3 | CCCAGCTTGTTGGGCCATGAGGACT | verru_2_3 | ACCCTAACTTTCGTCCATGAGCGTC |
| flavo_15_4 | CGGCGTACTCCCCAGGTGCATCACT | alpha_6_4 | ATCTTCTTGCGAGTAGCTGCCCACT | verru_2_4 | GCGTCGCATGTTCACACTTTCGTGC |
| flavo_15_5 | ACTCCCCAGGTGCATCACTTAATAC | alpha_6_5 | TCTTCTTGCGAGTAGCTGCCCACTG | verru_2_5 | CAAGTGTTCCCTTCTCCCCTCCAGT |
| flavo_15_6 | CGTACTCCCCAGGTGCATCACTTAA | alpha_6_6 | TAGCCCAGCTTGTTGGGCCATGAGG | verru_2_6 | TACACCAAGTGTTCCCTTCTCCCCT |
| flavo_15_7 | CCGGCGTACTCCCCAGGTGCATCAC | alpha_6_7 | GCCACTAACCCCGAAGCTTCGTTCG | verru_2_7 | CCAAGTGTTCCCTTCTCCCCTCCAG |
| flavo_15_8 | GTACTCCCCAGGTGCATCACTTAAT | alpha_6_8 | GTAGCCCAGCTTGTTGGGCCATGAG | verru_2_8 | ACACCAAGTGTTCCCTTCTCCCCTC |
| flavo_15_9 | GCCGGCGTACTCCCCAGGTGCATCA | alpha_6_9 | CGCCACTAACCCCGAAGCTTCGTTC | verru_2_9 | CGCTACACCAAGTGTTCCCTTCTCC |
| flavo_15_10 | GAAGAGAAGGCCTGTTTCCAAGCCG | alpha_6_10 | TTCTTGCGAGTAGCTGCCCACTGTC | verru_2_10 | CACCAAGTGTTCCCTTCTCCCCTCC |
| flavo_15_11 | CAACAGCGAGTGATGATCGTTTACG | alpha_6_11 | TAGCATCTTCTTGCGAGTAGCTGCC | verru_2_11 | GCTACACCAAGTGTTCCCTTCTCCC |
| flavo_15_12 | GCATGCCCATCTCATACCGAAAAAC | alpha_6_12 | AGCATCTTCTTGCGAGTAGCTGCCC | verru_2_12 | CTACACCAAGTGTTCCCTTCTCCCC |
| flavo_15_13 | TTGTAATCTGCTCCGAAGAGAAGGC | alpha_6_13 | GCCCAGCTTGTTGGGCCATGAGGAC | verru_2_13 | AGTGTTCCCTTCTCCCCTCCAGTAC |
| flavo_15_14 | CGCCGGTCGTCAGCAAAAGCAAGCT | alpha_6_14 | CACTAACCCCGAAGCTTCGTTCGAC | verru_2_14 | AAGTGTTCCCTTCTCCCCTCCAGTA |
| flavo_15_15 | AAGAGAAGGCCTGTTTCCAAGCCGG | alpha_6_15 | CATCTTCTTGCGAGTAGCTGCCCAC | verru_2_15 | ACCAAGTGTTCCCTTCTCCCCTCCA |
| flavo_15_16 | GCCGGTCGTCAGCAAAAGCAAGCTT | alpha_6_16 | TGTAGCCCAGCTTGTTGGGCCATGA | verru_2_16 | GCTACCCTAACTTTCGTCCATGAGC |
| flavo_15_17 | TGCCGGCGTACTCCCCAGGTGCATC | alpha_6_17 | AGCCCAGCTTGTTGGGCCATGAGGA | verru_2_17 | GTTCCCTTCTCCCCTCCAGTACTCT |
| flavo_15_18 | GCGCCGGTCGTCAGCAAAAGCAAGC | alpha_6_18 | CCACTAACCCCGAAGCTTCGTTCGA | verru_2_18 | GTGTTCCCTTCTCCCCTCCAGTACT |
| flavo_15_19 | CGAAGAGAAGGCCTGTTTCCAAGCC | alpha_6_19 | GCATCTTCTTGCGAGTAGCTGCCCA | verru_2_19 | TGTTCCCTTCTCCCCTCCAGTACTC |
| flavo_15_20 | CCAACAGCGAGTGATGATCGTTTAC | alpha_6_20 | GTGTAGCCCAGCTTGTTGGGCCATG | verru_2_20 | CCGCTACACCAAGTGTTCCCTTCTC |
| flavo_15_21 | GGAGTATTAATCCCCGTTTCCAGGG | alpha_6_21 | TGCGCCACTAACCCCGAAGCTTCGT | verru_2_21 | TTCCCTTCTCCCCTCCAGTACTCTA |
| flavo_15_22 | TGGAGTATTAATCCCCGTTTCCAGG | alpha_6_22 | CTCAAGCACCAAGTGCCCGAACAGC | verru_2_22 | GGCGTCGCATGTTCACACTTTCGTG |
| flavo_15_23 | TCCCCGTTTCCAGGGGCTATCCTCC | alpha_6_23 | CCAGCTTGTTGGGCCATGAGGACTT | verru_2_23 | CGCTACCCTAACTTTCGTCCATGAG |
| flavo_15_24 | TGCGCCGGTCGTCAGCAAAAGCAAG | alpha_6_24 | ACTAACCCCGAAGCTTCGTTCGACT | verru_2_24 | CCCTAACTTTCGTCCATGAGCGTCA |
| flavo_15_25 | AACAGCGAGTGATGATCGTTTACGG | alpha_6_25 | TCTTGCGAGTAGCTGCCCACTGTCA | verru_2_25 | ACCGCTACACCAAGTGTTCCCTTCT |
